# MutexaGPT: an intuition-to-design translator for physics-based enzyme engineering

**DOI:** 10.1038/s43588-026-01049-y

**Published:** 2026-09-10

**Authors:** Qianzhen Shao, Yinjie Zhong, Sebastian Stull, Xinchun Ran, Ning Ding, Kieran Nehil-Puleo, Ruizhe Yao, Han Xu, Zhongyue J. Yang

**Affiliations:** 1 Department of Chemistry, Vanderbilt University, Nashville, TN USA; 2 Department of Computer Science, Vanderbilt University, Nashville, TN USA; 3 Interdisciplinary Materials Science Program, Vanderbilt University, Nashville, TN USA; 4 Center for Structural Biology, Vanderbilt University, Nashville, TN USA; 5 Vanderbilt Institute of Chemical Biology, Vanderbilt University, Nashville, TN USA; 6 Data Science Institute, Vanderbilt University, Nashville, TN USA; 7 Department of Chemical and Biomolecular Engineering, Vanderbilt University, Nashville, TN USA

**Keywords:** Biocatalysis, Molecular dynamics, Computational biophysics

## Abstract

Physical intuition about how enzyme structure and dynamics shape function has guided successful engineering efforts, yet a systematic approach is still lacking for translating these qualitative and abstract ‘thoughts’ into quantitative, actionable principles for enzyme design. Here we introduce MutexaGPT, an open-access, multi-agent large language model platform that translates enzyme engineering intuition to physics-based simulations and thus variant designs. Through a web-interface, MutexaGPT takes plain-English, intuition-driven requests as input and leverages large language model agents to elicit missing information, construct physics-based models, configure and execute high-throughput molecular modeling workflows, and convert the results into actionable design proposals, such as smart mutation libraries. We demonstrate the utility of MutexaGPT in two protein engineering tasks: (1) engineering halide methyltransferase toward bulkier substrates and (2) engineering bidomain amylase for enhanced activity at lower temperature. These results establish MutexaGPT as an intuition-to-design translator that integrates human creativity with high-throughput molecular modeling to democratize physics-guided, intuition-driven enzyme engineering.

## Abstract (web summary)

MutexaGPT turns plain-language enzyme-engineering intuitions into lead mutation designs through AI agent-orchestrated high-throughput molecular modeling, yielding experimentally validated improvements in enzyme specificity and cold activity.

## Main

Physical intuition^1,2^ has been a cornerstone of enzyme engineering. It arises from accumulated knowledge and experience in rationalizing how enzymes realize their catalytic functions through three-dimensional structural topology (for example, active-site cavities and tunnels^3–11^), conformational dynamics (for example, flexibility and domain motion^12–19^), electronic structure (for example, electrostatics and electric fields^20–25^) and interactions with the surrounding environment (for example, hydration shell). Such intuition helps protein engineers infer or anticipate the impact of mutations on enzyme activity^26–29^, temperature adaptation^30^, selectivity^31–35^, promiscuity^36–41^ and stability of enzymes^42,43^. For instance, steric intuition in amine transaminases has led to a design rule for tuning stereoselectivity: by adjusting the volumes of the large and small binding pockets within the active site^3–8^ through site-directed mutagenesis to better match the sizes of the corresponding ketone substituents, one can invert selectivity or expand substrate scope^33,44,45^. Such intuition also strengthens the screening-based, directed evolution approaches for effective function-enhancing mutant discovery. Reetz and coworkers exemplified this through developing the combinatorial active-site saturation test (CASTing) method, which applies targeted saturation mutagenesis to small residue sets and recombines beneficial variants to capture cooperative effects for enhancing activity and selectivity toward non-native substrates^32,34,35,37,46–48^. In essence, physical intuition guides the construction of ‘smart’ mutational libraries and facilitates effective enzyme engineering when high-throughput screening assays are not available.

Despite its importance, translating physical intuition into successful enzyme engineering outcomes remains difficult. In practice, most intuitive catalytic principles in enzymology are qualitative rather than quantitative. Gordon Hammes’ famous notion that “For catalysis (of an efficient enzyme), flexible but not too flexible, as well as rigid but not too rigid, is essential” captures a conceptual truth yet provides no guidance for identifying the optimal degree of flexibility^49^. Even when an intuition has a quantitative foundation, such as the correlation between electric field strength and catalytic rate enhancement^20–25^, it remains unknown how specific mutations should be introduced to achieve the desired electrostatic environment. As a result, the unique intuition or a stroke-of-genius inspiration from protein engineers always fails to get translated into a ’smart’ mutational library or a concrete protein engineering strategy. This makes it inevitable to adopt a hypothesis-agonistic approach by screening against a larger library for mutant discovery, leading to greater labor/resource cost and longer enzyme development cycle in most enzyme engineering scenarios.

The pressing challenge facing the entire field of enzyme engineering is how to establish an enzyme engineering paradigm that translates physical intuitions into quantitative, actionable design principles. One promising approach to address this challenge requires high-throughput molecular simulations (htMS), which quantify how mutations alter enzyme structure, dynamics, and energetics to predict their effects on catalytic activity and selectivity. For example, SubTuner leveraged htMS (specifically quantum mechanics and molecular dynamics (MD) simulations) to translate physical hypotheses (such as optimal transition state binding and electric field stabilization) into quantitative metrics in the in silico screening workflow and eventually yield smart libraries (approximately ten mutants for each task) that constantly achieved ~30% hit rate across three transferase engineering tasks. Similarly, CASCO^50^ leveraged high-throughput MD simulations to quantify substrate positioning dynamics as near-attack-conformation frequencies in the engineering of the enantioselectivity of a limonene epoxide hydrolase; QzymeDesigner^44^ used semi-empirical quantum mechanics to quantify the substrate binding as free energies in the engineering of the substrate scope of the ω-transaminase from *Chromobacterium violaceum*; and AI.zymes^51^ integrated MD screening into a multi-objective design workflow, using metrics such as electric field strength, ligand flexibility and so on to prioritize variants for improving the promiscuous Kemp eliminase activity in a ketosteroid isomerase. Yet, despite its demonstrated success, htMS by itself cannot fully bridge the gap between intuition and enzyme engineering practice; its complexity and technical demands often limit accessibility beyond specialized computational experts. As a result, numerous potentially transformative approaches for intuition-guided, molecular modeling-based enzyme engineering have stalled as one-off proof-of-concepts rather than scalable solutions.

**Fig. 1:**
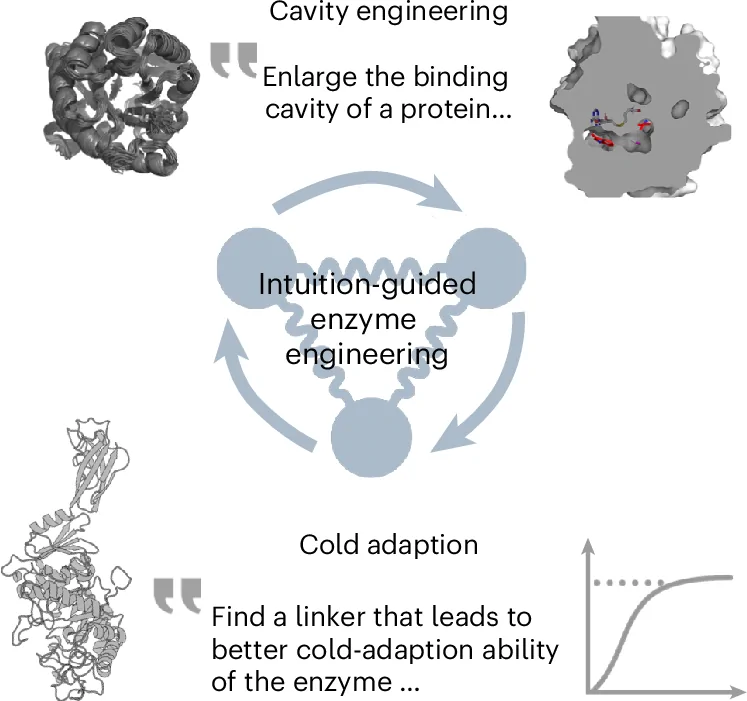
Physical intuition-guided enzyme engineering. Physical intuitions about enzyme structure and dynamics can motivate design ideas such as enlarging active-site cavities or tuning linker-mediated domain organization, and MutexaGPT translates these qualitative ideas into physics-based modeling tasks and thus actionable designs.

To fully unlock the potential of intuition-guided enzyme engineering, we propose a computational platform that unites large language models (LLMs) with htMS. Our solution satisfies two critical needs simultaneously: (1) translating qualitative and descriptive intuition into explicit, executable instructions and (2) converting those instructions into automated computational workflows that deliver quantitative, physics-based outputs. To this end, we developed MutexaGPT, an intuition-to-design translator of molecular simulation for enzyme engineering. MutexaGPT is built based on LLM agents and works as an open-access web application (https://enzyhtp.app.vanderbilt.edu) that interacts with users in plain English, converts intuition-based requests from users into computational modeling plans, performs high-throughput MD simulations, analyzes the results and summarizes them into actionable insights for enzyme engineering (such as a ‘smart’ library pinpointing a few mutants). Note that although MutexaGPT ranks and filters enzyme variants based on computed metrics, it is not a predictive artificial intelligence (AI) tool but instead an integrated system for automation and intuition-translation. Designed for protein engineers and designers with little or no molecular modeling expertise, MutexaGPT is distinct from existing computational chemists-oriented LLM-agentic molecular simulation tools (for example, MDCrow^52^, NAMD-agents^53^, DynaMate^54^ and ProtAgents^55^) that require users’ domain knowledge to choose theoretical models, design simulation protocols and interpret results. We achieve so by equipping MutexaGPT’s LLM agents with necessary domain knowledge to guide users through the entire life cycle of high-throughput physics-guided enzyme engineering^56,57^, from creating the scientific question to interpreting the results. We demonstrated the effectiveness of MutexaGPT through two cases that showcase how empirical intuition of cavity engineering and statistics-based intuition of cold-adaptation engineering can be converted to physics-based models and guide function-gaining mutant discovery (Fig. 1b). Complementary to screening-dominated approaches, MutexaGPT provides a scalable route to operationalize intuition-driven, physics-guided enzyme engineering within the scope of currently implemented metrics and simulations.

## Results

**Fig. 2:**
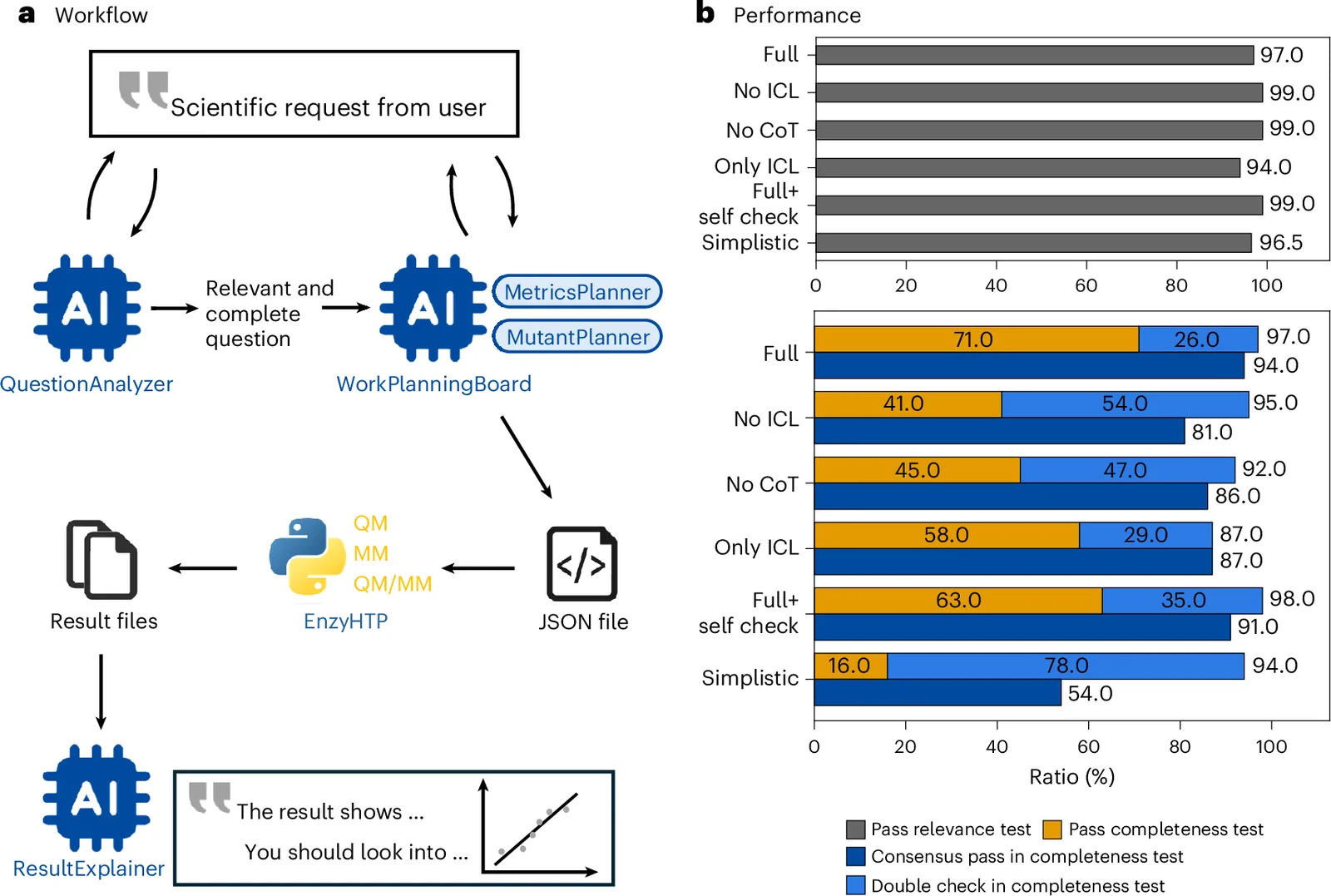
The design of MutexaGPT. **a**, The architecture and workflow of the multi-agent system in MutexaGPT. With a user posting their scientific request, QuestionAnalyzer checks the request for the missing details, provides suggestions to better define the scientific question, helps the user articulate their assumptions and, eventually, co-creates a scientifically sound and concrete request. The request is then sent to WorkPlanningBoard (consisting of MetricsPlanner and MutantPlanner) to come up with a molecular modeling plan and seek approval from the user. The user-confirmed plan is then saved as a JSON file, which is sent to the EnzyHTP in the backend for performing the high-throughput simulations. ResultExplainer dissects the result files from the simulation and summarizes them into a concise report for the user. QM, quantum mechanics; MM, molecular mechanics. **b**, Benchmark of system prompt engineering strategies in the QuestionAnalyzer for relevance (top) and completeness (bottom). The full evaluation results for all agents are summarized in SI.zip/agent_evaluation. Full, full prompt; No ICL, full prompt with ICL part removed; No CoT, full prompt with the chain of thought part removed; Only ICL, a prompt containing only ICL examples; Full + self-check, augmenting the ‘Full’ prompt with a one-line self-reflection instruction; Simplistic, only contains a sentence of role description and the output format in the system prompt. Source data

At the heart of MutexaGPT is a multi-LLM-agent system that consists of QuestionAnalyzer, WorkPlanningBoard and ResultExplainer (Fig. 2a; see ‘Implementation of MutexaGPT, the workflow and the multi-agent system’ in the Methods). In brief, QuestionAnalyzer first clarifies missing or ambiguous scientific information from user’s intuition-based request; WorkPlanningBoard then translates the refined request into computable metrics and mutation patterns, which are passed to EnzyHTP for execution of the corresponding htMS in the backend; and ResultExplainer converts the simulation outputs into ranked variants, interpretation boundaries and follow-up recommendations. We further evaluated these agents using systematically curated test cases and automated judging systems, comparing different prompt designs and confirming that the final designs reliably support their respective tasks in the workflow (Fig. 2b; see ‘Implementation of MutexaGPT, the performance of agents’ in the Methods). Full details of the implementation, agent design and systematic agent evaluation are provided in the Methods.

The following sections present two MutexaGPT-enabled enzyme-engineering campaigns that translate intuition (written in plain English) into physics-based models, molecular simulation workflows and, eventually, actionable design recommendations. First, a cavity-engineering task uses high-throughput MD ensemble analysis to pinpoint mutations that enlarge an active site, improving accommodation of bulkier, non-native substrates; the resulting mutants were then tested retrospectively against previously reported experimental screening results. Second, a cold-adaptation engineering task uses high-throughput MD to design linker variants that enhance enzyme activity at low temperatures; the resulting design were subsequently evaluated by our experimental activity assays. In both cases, a shared obstacle that often deters enzyme engineers from translating their intuition into design is the absence of quantitative and actionable guidance and the steep expertise required for high-throughput physics-based modeling. Below, we demonstrate how MutexaGPT addresses these challenges and leads to scientific discoveries and understandings.

### MutexaGPT for cavity engineering to modify substrate specificity

In the first case, we asked MutexaGPT to engineer the cavity of a halide methyltransferase toward catalyzing bulkier substrates for group transfer reactions. Cavity engineering targets the shape and volume of the interior voids of an enzyme, and designs or screens mutations accordingly to modulate the enzyme’s functions and properties. An empirical intuition in cavity engineering is ‘larger cavity allows the enzyme to accommodate larger substrates with enhanced promiscuity’. Although static active-site structures have enabled successful cavity-guided engineering in several systems^32,33,44,58,59^, such intuition is difficult to apply broadly. Simple mutations that appear to enlarge a cavity often fail because conformational dynamics can drastically reshape the pocket. As a result, most cavity engineering efforts still rely on screening of a large mutational library, consuming substantial experimental resources.

**Fig. 3:**
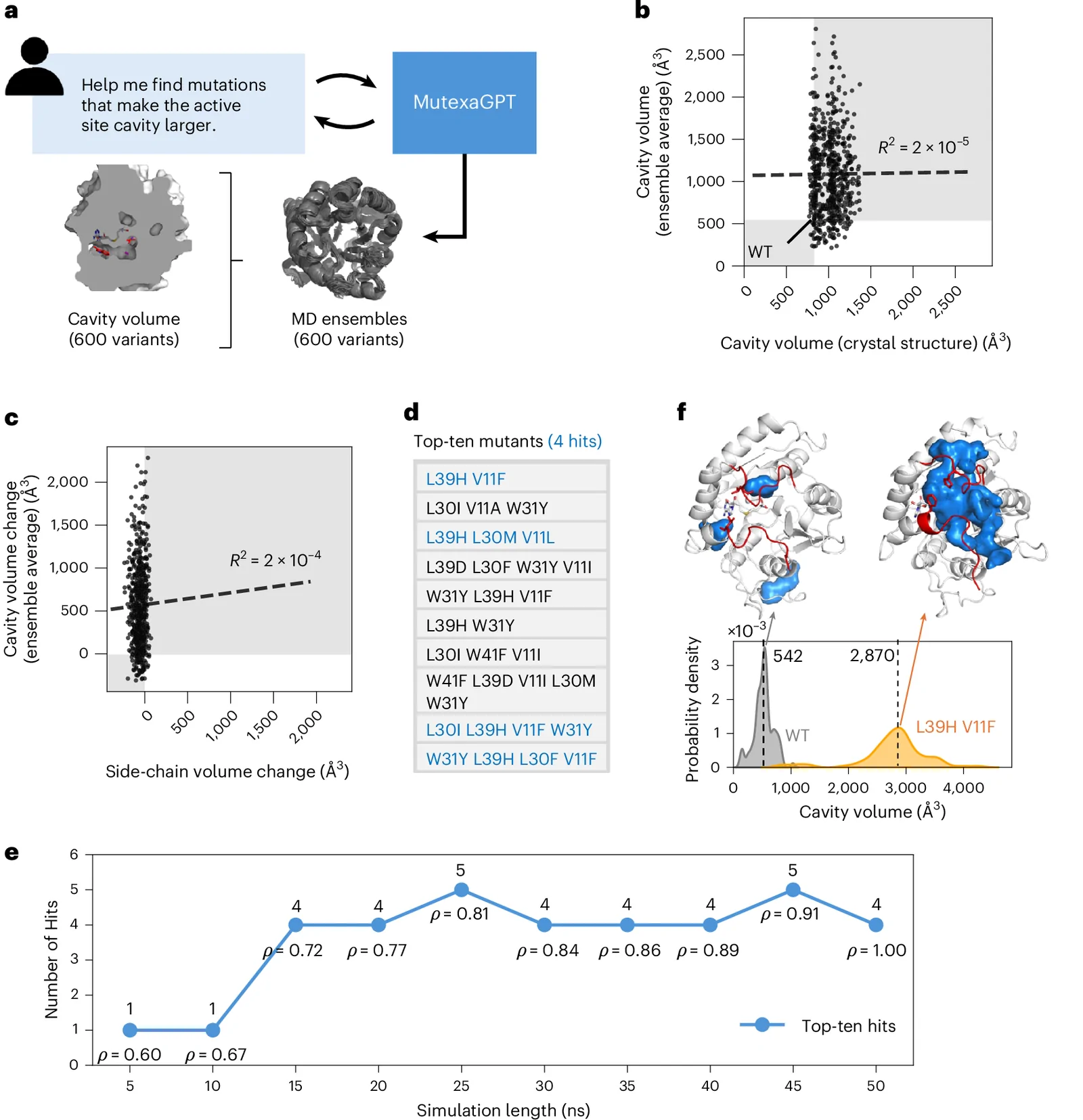
An overview of MutexaGPT-guided cavity engineering. **a**, Workflow overview: the user sent a plain-English request, MutexaGPT asked for more information and discussed with the user about the modeling plan and MutexaGPT performed high-throughput MD simulations and calculated cavity volumes for the library of 600 mutants. **b**, Correlation between cavity volumes calculated from direct geometric perturbation of static crystal structures (Methods) and those calculated from MD ensemble averages; both for the library of 600 mutants. **c**, Correlation between the change in side-chain volume versus the change in ensemble average cavity volume for the library of 600 mutants. **d**, MutexaGPT’s final list of ten mutants with the largest predicted cavity volumes. Experimentally confirmed hits are highlighted in blue; these mutants show activity with bulkier substrates^60^. **e**, The top-ten hit-rate plot against the MD simulation length of each mutant. The Spearman correlation coefficient (*ρ*) is noted under each data point. **f**, Cavity volume distribution of a top-ranked mutant, L39H V11F against that of the WT. The probability distribution of the mutant’s cavity volume (L39H V11F, shown as orange) is shifted to a much larger value (~2,870 Å^3^) compared with the WT (shown as gray) enzyme (~542 Å^3^). Representative structures from the distribution center (dotted line, labeled) show how mutations (stick, red) induce a conformational change in an active site loop (cartoon, red), leading to a substantially larger and more open active site (surface, blue). The kernel density distribution is fitted using the gaussian_kde function from the scipy package (data are obtained from 100 snapshots sampled from the MD trajectory). Source data

MutexaGPT allows the user to translate such intuition into concrete physics models and testable designs through high-throughput MD simulations. The user can start a cavity engineering task by sending a plain-English request in the chat. In our demo, the user sent ‘help me find mutations that make the active site larger’ (Fig. 3a). MutexaGPT evaluated the request and found missing scientific details that were essential to the subsequent modeling (that is, a target enzyme structure and a list of desired mutations). As such, it asked the user to provide a Protein Data Bank (PDB) file of the target enzyme and a description of mutations that the user wants to include (see full conversation log in Supplementary Section 1). To address this request, the user uploaded the PDB structure of the wild-type (WT) halide methyltransferase and supplied details of a mutation library (that is, 600 combinatorial mutations from five sites; Supplementary Section 1) previously constructed for this enzyme by Schülke et al^60^. The experimental sequence–activity data derived from the library screening against five substrates of diverse sizes allow us to investigate how changes in cavity volume influence enzyme activity later on. In real-world applications, users can initiate their protein engineering campaigns with MutexaGPT by simply describing their desired mutation library, such as ‘1,000 random mutations across the enzyme’, ‘single-point saturation mutations within 5 Å of the heme cofactor’ and so on.

After verifying that the user’s input contained all necessary information to proceed with the modeling, MutexaGPT planned a relevant molecular simulation task: calculating the cavity volume of the active site of each mutant from MD-sampled conformational ensemble of the holo-enzyme (see the Methods for full computational details). With the user’s approval, the backend executed the corresponding computational workflow. For each of the 600 multi-point mutants in the library, MutexaGPT generated a 50-ns MD trajectory and analyzed the distribution of active site volumes across the trajectory. The length of each MD simulation is an option that the user can specify in the frontend before submitting the job. We used 50 ns here to mimic a realistic scenario where most enzyme engineers do not have access to extensive computational resources and need to balance computational cost and sampling adequacy. Even with this moderate MD simulation length, it would still demand 30-μs sampling (that is, 600 mutants × 50 ns) to cover the entire mutation library.

Eventually, MutexaGPT addressed the user’s initial request ‘help me find mutations that make the active site larger’, by ranking all 600 variants according to their ensemble-averaged cavity volumes and identifying the ten candidates showing the greatest increases in volume (Fig. 3d; see screenshot of the summary page in Supplementary Fig. 3), thereby turning the original empirical intuition into testable mutation designs. To validate these designs, we compared MutexaGPT’s top-ranked mutations with experimentally characterized variants reported by Schülke et al^60^. Among the ten top-ranked variants, four were confirmed experimental hits based on the prior study by Schülke et al., yielding a 40% hit rate and around fourfold improvement over the baseline (see Supplementary Section 12 for details about the baseline). To investigate whether 50-ns MD simulations are sufficient for cavity-based smart library construction, we systematically examined how simulation length influences hit enrichment and ranking fidelity (Fig. 3e). Very short simulations (5–10 ns) fail to enrich hits beyond the baseline expectation (that is, approximately one hit), whereas simulations of 15 ns or longer consistently identify at least four hits, corresponding to about a fourfold enrichment. Meanwhile, the Spearman rank correlation with the 50 ns reference increased monotonically with simulation length, indicating progressively more stable cavity-volume estimates. Together, these results show that moderate-length (that is, 15 ns or longer) MD simulations provide reliable quantitative guidance for mutation prioritization in cavity-engineering campaigns.

A case study of the top mutant, L39H V11F, illustrates how mutations induce a drastic upshift in the active site’s cavity volume distribution compared with the WT enzyme (Fig. 3f), thereby reshaping its substrate preference. The cavity volume for the most probable WT conformation is approximately 542 Å^3^, corresponding to a closed and compact active site. By contrast, the L39H V11F mutant’s cavity volume distribution is centered at a much larger value of ~2,870 Å^3^, reflecting a substantially larger and more accessible cavity. This transition from a closed to an open cavity state is primarily driven by the altered conformational dynamics of two key active site loops (residue 1–11 and residue 36–46; Fig. 3f, red). This cavity expansion in the active site directly translates to improved substrate promiscuity toward a variety of substrates, especially the sterically bulkier ones. While the WT is evolved by the nature to catalyze the methyl transfer from iodomethane to *S*-adenosyl-l-homocysteine (SAH), L39H V11F showed an substantially increased specific activity (relative to the WT) for bulkier substrates^60^ such as iodoethane (10-fold activity improvement), 1-iodopropane (49-fold), 1-iodobutane (50-fold), (iodomethyl)cyclopropane (50-fold) and (2-iodoethyl)benzene (29-fold). These results demonstrate that MutexaGPT can effectively convert intuition into physics models and design through high-throughput molecular modeling, identifying function-enhancing mutations that alter substrate preference.

Given the computational expense of MD simulations, an important question arises: is MD truly necessary for quantitative cavity engineering, or can the ensemble-averaged cavity be reliably inferred from a single static structure as a shortcut? Using the MD trajectories generated by MutexaGPT for 600 variants, we first examined the correlation between the ensemble-averaged cavity volume from MD and that calculated from a single static PDB structure (with optimized side-chain rotamers), assessing whether static structures can adequately serve as surrogates for dynamic ensembles in computational enzyme engineering. The results show no correlation (*R*^2^ ≈ 2 × 10^−^^5^) between the static and MD-averaged volumes, where the cavity volumes derived from MD trajectories distribute substantially broader than from static PDB structures (standard deviation of 488 Å^3^ versus 145 Å^3^, Fig. 3b). This lack of correlation originates from the presence of cryptic cavities that emerge only in the MD ensemble through conformational fluctuations. These transient pockets remain closed or buried in the crystalline state represented by a single static PDB structure. As a second test, we evaluated another common cavity engineering shortcut that estimates the magnitude of cavity expansion in the mutant from the net decrease in side-chain volume due to point mutations. Specifically, we tested the correlation between the change in side-chain volume and the change in ensemble-averaged cavity volume derived from MD trajectories. This approximation also fails to show any meaningful correlation (*R*^2^ ≈ 2 × 10^−^^4^; Fig. 3c), indicating that cavity change originates from collective and dynamic rearrangements of the protein active-site structure rather than from simple steric subtraction, which explains why traditional qualitative intuition-based designs often fail. Together, these two tests demonstrate that simple approximations cannot capture the intrinsic conformational plasticity of enzyme cavities, underscoring the necessity of physics-based modeling, specifically, MD simulations for predictive cavity engineering.

To further test whether MutexaGPT remains useful when prior information about mutational hotspot is minimal, we designed a test where the mutation library is selected solely based on a distance cutoff from the substrate. In this new case, the user starts by asking ‘help me find mutations that make the enzyme faster for an non-native substrate ethyl iodide’, and during the user’s conversation with the QuestionAnalyzer, the mutation library was defined by user as ‘I want to explore all single point mutations with in 4 Å of ETI’, where ETI is the three-letter name of the target substrate (that is, ethyl iodide) in user’s PDB files. After the conversation, MutexaGPT plans a workflow that calculates the folding free energy and the electric field strength projected to the breaking C–I bond for each enzyme mutant. With the simulation data, MutexaGPT summarizes the results by filtering candidates with folding free energy and ranking by electric field, yielding a top-ten mutant list, of which three mutants are experimentally active in a previous screening reported by Tang et al.^61^, corresponding to a hit rate of 30%. Notably, this 30% value serves a lower bound because four mutants on this list were not experimentally characterized (not labeled in our test) and could be a hit. Overall, this low-prior-knowledge setup results in a similar prediction performance to the cavity engineering example, supporting MutexaGPT’s ability to convert broadly-defined, less-informed design intent into actionable screening priorities.

### MutexaGPT for linker engineering to enhance cold adaptation of enzymes

**Fig. 4:**
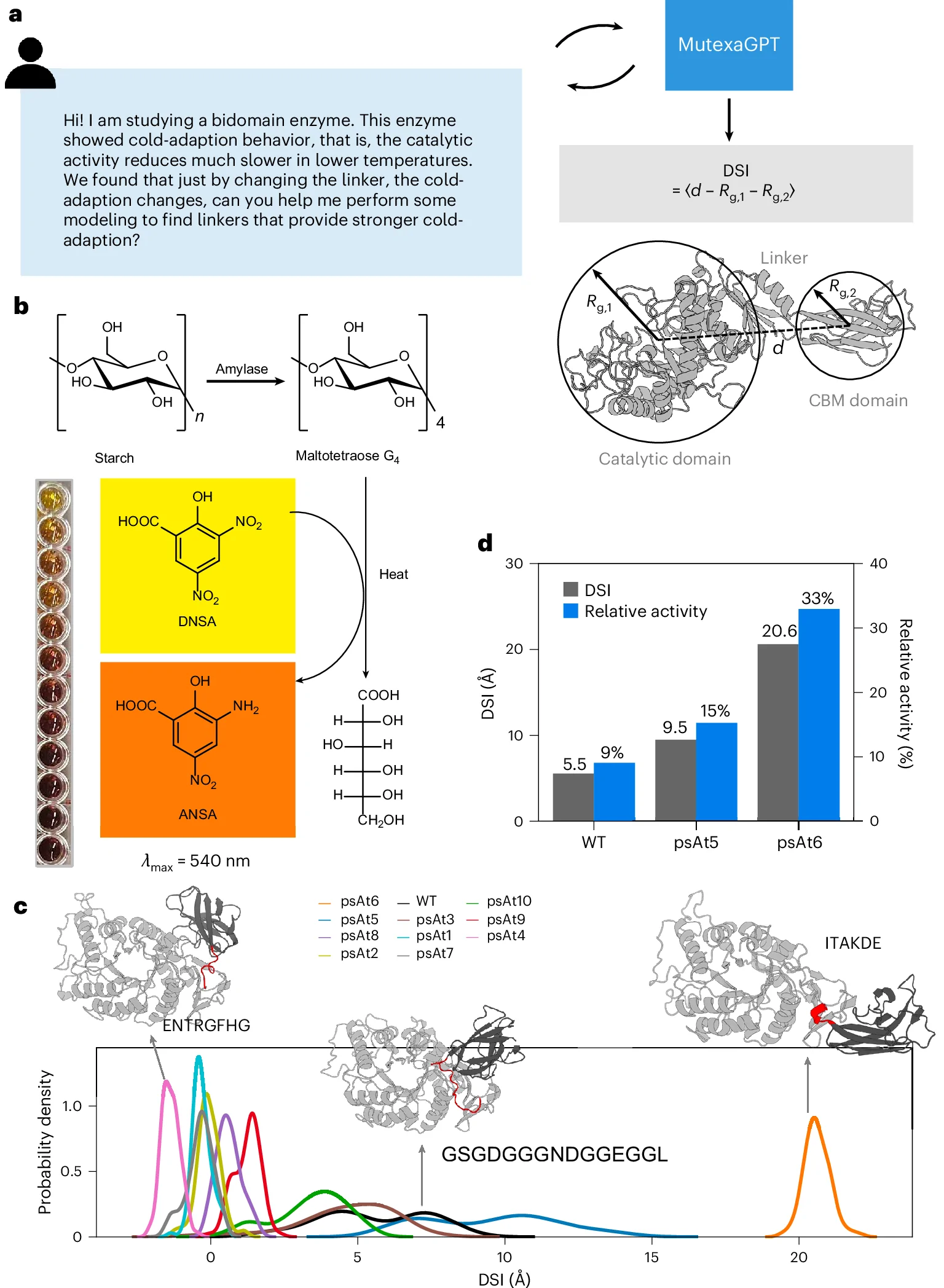
MutexaGPT-guided linker design for cold-adapted bidomain amylases. **a**, Workflow overview: the user sent a request in plain text; MutexaGPT collected missing information from user, discussed and planned the modeling, performed MD for WT and ten linker-replaced variants, and calculated the DSI. DSI is defined as 〈*d* − *R*_g,1_ − *R*_g,2_〉 over the MD ensemble, where *d* is the distance between the two domain centroids, and *R*_g,1_/*R*_g,2_ are the radii of gyration of the two domains, respectively (units are displayed in ångströms). **b**, The experimental assay for the amylase activity: specific activity is measured via the DNSA reducing‑sugar assay; cold‑adaptation is evaluated as the ‘relative activity’, which is the activity at 0 °C divided by activity at 45 °C. ANSA, 3-amino-5-nitrosalicylic acid. **c**, The DSI distribution of the psA WT and its ten linker variants. For variants with the largest (psAt6) and smallest (psAt4) average DSI and the WT, a representative structure of the distribution peak is attached (gray, catalytic domain; red, linker; dark gray, carbohydrate-binding module (CBM) domain). The linker sequences are noted next to each structure. Note that the WT shows two equally distributing peaks (see Supplementary Fig. 10 for representative structures of both peaks). The kernel density distribution is fitted using the gaussian_kde function from the scipy package (data were obtained from 100 snapshots sampled from the MD trajectory of each variant). **d**, The experimental results of MutexaGPT suggested two variants (psAt5 and psAt6) that were predicted to have higher DSI than WT also showed increased relative activity in experiments (see full experimental and computational detail in the Methods). Source data

In the second case, we integrated MutexaGPT with experimental assays to engineer cold-adapted variants of a bidomain amylase by redesigning its interdomain linker, aiming to enhance enzymatic activity at low temperatures. Cold-adapted enzyme engineering, the process of identifying enzyme variants that maintain high activity at colder conditions (for example, 0–15 °C), is crucial for low-temperature laundry^62^, food processing^63,64^, bioremediation^65,66^ and single-cell sequencing^67^, serving as a cornerstone for modern biomanufacturing and biotechnology. From MD modeling, we uncovered a ‘statistical intuition’ that suggests the correlation between increased interdomain separation and enhanced cold adaptation in bidomain amylases and cellulases^30,68^. This separation is quantified by the spatial proximity between the carbohydrate-binding module and the catalytic domain, defined as the domain separation index (DSI) (Fig. 4a). Guided by this statistical intuition, MutexaGPT can help user establish a closed-loop computational–experimental workflow. In this workflow, MutexaGPT identified linker variants of a maltotetraose-forming amylase (Fig. 4b) predicted to exhibit increased DSI, and these variants were subsequently tested for enhanced cold adaptation through enzyme activity assays.

The user initiated the task by sending the following request to MutexaGPT: “I am studying a bidomain enzyme. This enzyme showed cold-adaptation behavior, that is, the catalytic activity reduces much slower at lower temperatures. We found that just by changing the linker, the cold-adaption changes, can you help me perform some modeling to find linkers that provide stronger cold-adaption?”. Similar to the first case, MutexaGPT asked for clarification of the target enzyme by requesting PDB files and the linker sequences of interest. The user provided the AlphaFold2-predicted structure of the mesophilic *Pseudomonas saccharophila* amylase (psA), as well as ten psA variants with different linker sequences selected from natural peptide linkers in LinkDB^69^ and SynLinker^70^ databases based on the linker topology from our previous study (see Supplementary Table 2 for linker sequences and Supplementary Section 13 for the selection workflow). With this information, MutexaGPT devised a plan to identify linker variants predicted to exhibit higher DSI values than the WT by performing 50-ns MD simulations for each candidate. Upon the user’s confirmation, MutexaGPT executed the simulations, analyzed the trajectories and summarized the DSI results for all variants (see Supplementary Section 2 for the full dialog and Supplementary Table 2 for the list of DSI values).

The DSI distributions for all ten linker variants (psAt1–psAt10) display a distinct pattern (Fig. 4c). We observe that variants with extreme DSI values tend to adopt less fluctuating interdomain conformations. psAt6, for example, shows the highest average DSI (20.6 Å) with a very narrow distribution (variance of 0.2 Å), while psAt4, psAt7, psAt1, psAt2, psAt8 and psAt9 cluster near 0 Å show similarly tight distributions (variance of 0.1–0.2 Å). By contrast, the WT and variants with moderate DSI values, including psAt3, psAt10 and psAt5, exhibit much broader DSI distributions (variance of 1.4–4.8 Å). These patterns arise from distinct linker scaffolds. In high-DSI variants, the two domains are well separated and connected by a linker that predominantly adopts an α-helical conformation (Fig. 4c, right), restricting relative domain motion. In low-DSI variants, the two domains pack closely and form extensive interdomain contacts, likewise limiting flexibility (Fig. 4c, left). By contrast, variants with intermediate DSI values, as well as the WT, feature a more disordered linker that samples diverse orientations between the domains, leading to broader DSI distributions.

Two variants, psAt5 (blue) and psAt6 (orange), center around 9.5 Å and 20.6 Å, respectively, substantially exceeding the DSI range of the WT (around 5.5 Å). These two variants were therefore suggested by MutexaGPT for further experimental characterization (Fig. 4b). We then experimentally assessed the specific activity of these two variants at different temperatures by measuring the amount of reducing sugar produced from starch using a 3,5-dinitrosalicylic acid (DNSA) assay^71^ (Fig. 4b). The magnitude of cold adaptation was assessed by calculating the relative activity, defined as the ratio of the enzyme’s activity at 0 °C to its activity at 45 °C. The experimental results (Fig. 4c) show that psAt5 and psAt6 exhibit 1.7-fold and 3.7-fold increases in relative activity, respectively, compared with the WT enzyme, indicating that both variants involve enhanced cold adaptation (see Supplementary Table 3 for activities under each temperature). Together, these results validate MutexaGPT’s capability to seamlessly convert statistical intuition into physics-based models, integrate with experimental workflows and conduct physics-guided engineering of cold-adapted enzymes.

## Discussion

We developed MutexaGPT as an intuition-to-design translator for enzyme engineering. By integrating a multi-LLM-agent system with EnzyHTP-based high-throughput molecular modeling, MutexaGPT converts plain-English engineering requests into actionable designs. Importantly, MutexaGPT is not a predictive AI tool but an automation and intuition-translation system that prioritizes variants using computed molecular metrics. We demonstrated MutexaGPT in two case studies. For halide methyltransferase cavity engineering, MutexaGPT achieved a 40% hit rate and ~4-fold enrichment over the baseline. For bidomain amylase linker engineering, MutexaGPT identified variants with up to 3.7-fold improved cold adaptation at 0 °C. Complementary to existing screening-heavy approaches, we believe MutexaGPT will enable a paradigm for enzyme engineering where human creativity is seamlessly integrated with computational simulation to make discoveries and designs.

How does MutexaGPT work beyond the two case studies? Currently, MutexaGPT supports high-throughput calculations of a list of enzyme function-related metrics: electric field strength, substrate positioning index, active site RMSD, MM-PB/GBSA ligand binding energy, relative folding free energy, cavity volume and DSI. Beyond the non-native substrate accommodation (metric: cavity volume) and cold adaptation (metric: DSI) as demonstrated in the current study, this full metrics list can be applied to a large variety of enzyme engineering tasks. For example, the cavity volume metric guides the engineering of enantioselectivity^33,44,45^. Electric field strength can guide the engineering of rate enhancement for enzymes that catalyze reactions involving a substantial change of charge and polarity state from the reactant state to the transition state^20–25^. MMPB/GBSA ligand binding energy can guide the engineering of enzyme with enhanced binding affinity to the substrate or reduced product inhibition^72^. Substrate positioning index can guide the engineering of catalytic efficiency for enzymes where precise substrate positioning is critical for catalysis^73^. Relative folding free energy can be used to guide the engineering of enzyme stability. Combining these metrics is expected to address more complex challenges, such as studying drug resistance mutations. Note that a practical limitation of MutexaGPT also comes from this list of computable metrics. If a property of interest from the user cannot be mapped to a currently supported EnzyHTP-computable metric, MutexaGPT cannot produce a physically grounded simulation answer and should instead return an explicit unsupported-property notice. Nevertheless, MutexaGPT is designed to be extensible, where new metrics can be easily (by changing just two files if they are supported by EnzyHTP) added in the future to support more enzyme engineering tasks (one can refer to Supplementary Table 1 for currently supported metrics; we should note that the underlying EnzyHTP engine is developed with interface modules to common software such as Amber^74^ for trajectory analysis, Gaussian^75^/xTB^76^ for quantum mechanics, Multiwfn^77^ for wavefunction analysis, Rosetta^78^ for biophysics and so on, and thus inherits the metrics support boundary of these software). Overall, we envision MutexaGPT can be applied to a wide range of enzyme engineering tasks by leveraging the existing metrics and adding new metrics in the future.

Finally, we discuss MutexaGPT’s level of autonomy as an AI agent. Gao et al.^79^ summarized the three levels of autonomy in AI agents: assistant level, which works with restricted range of tools (for example, web search, docking and so on) and relies on domain experts to define scientific hypotheses, propose theoretical models and design specific tasks; collaborator level, which collaboratively refine hypotheses with human, design computational tasks and interpret results; and scientist level, which develops and extrapolates scientific hypotheses beyond the scope of prior research, autonomously designs and performs experiments, synthesize concepts beyond summarizing the results and eventually make scientific discoveries all by itself. Existing LLM-agentic molecular simulation tools mostly operate at the assistant level, designed to streamline the molecular modeling process for domain experts^79^. By contrast, MutexaGPT is featured by its functionalities that one would typically expect from a collaborator, such as refining intuition into physics-based hypotheses, executing the htMS and generating design recommendations. For protein engineers with little-to-no experience in computational modeling, MutexaGPT, while remaining an assistant-level system, takes a step toward becoming a collaborator-level agent for enzyme engineering.

However, the level of hypothesis refinement is still limited to correcting nonscientific statements, retrieving missing information for a vague request and mapping general properties of interest to specific computable metrics. Presumably, a complete collaborator level agent should also be able to proactively generate alternative mechanistic hypotheses rather than merely refining user-proposed ones, design and adapt multi-step computational and experimental workflows to discriminate among these hypotheses, integrate heterogeneous evidence across modalities to revise or reject initial ideas and autonomously propose new lines of inquiry when the accumulated data reveal gaps or inconsistencies. Such limitation comes from the limited reliability and robustness of current foundation models for complex, open-ended scientific reasoning, the lack of mature methods for uncertainty quantification and self-evaluation, and the scarcity of standardized, high-quality multimodal datasets and workflows needed to safely couple in silico molecular modeling with real-world experimentation. With the unprecedented evolving speed in AI technology and foundation models, we expect these limitations to be progressively alleviated and MutexaGPT will move closer to a genuinely collaborator-level agent.

## Methods

### Computational method

#### Protein structure preparation

In the cavity engineering use case of MutexaGPT, the uploaded WT structure of *Aspergillus clavatus* methyltransferase (acl-MT) is obtained from the AlphaFold Protein Structure Database (UNIPROT ID: A1CIS5). The SAH cofactor is placed in the structure referencing the binding position in the crystal structure of a homologous anion methyltransferase from *Arabidopsis thaliana* (PDB 3LCC). In the cold-adaptation engineering use case of MutexaGPT, the uploaded WT structure of psA is obtained from our previous study^30^, which used AlphaFold2^80^ via the ColabFold^81^ server to predict the protein structure from its sequence. The sequence and parameters used for prediction are listed in Supplementary Tables 3 and 4. The rest of the structure preparation procedures, including determining the protonation states of titratable residues, generating structures of enzyme mutants and so on, are planned and performed automatically by MutexaGPT, powered by EnzyHTP^56,57^ in the backend (via the protonate_stru(), mutate_stru(), assign_mutant() and so on) application programming interfaces (APIs) in EnzyHTP (see full API parameters and underlying algorithms in refs. ^82,83^).

#### MD

All MD simulations in this study are performed autonomously on the MutexaGPT server. In the server backend, EnzyHTP is used to perform the MD simulations via the equi_md_sampling() API. This API uses a default force field combination of AMBER ff14SB^84^ for the protein, TIP3P^85^ for water and the generalized AMBER force field^86,87^ for ligands with the AM1-BCC^88^ charge model. This API, by default, solvated the enzyme in a periodic truncated octahedron box with a 10 Å buffer of water and were neutralized with Na^+^ or Cl^−^ counterions. The production time was set to 50 ns via the web GUI in the frontend of MutexaGPT. In the equi_md_sampling() API, via the underlying interface with AmberMD^89^, each 50-ns MD production run is preceded by (1) 20,000 steps energy minimization of the entire simulation system using the steepest descent method for the first half and the conjugate gradient method for the other half; (2) 50-ps heating from 0 to 300 K with constant volume and the backbone C_α_, C and N of the amide group restrained with a weight of 2 kcal mol^−1^ Å^−2^; (3) 500-ps equilibration at 300 K and 1 atm with the same backbone restraint; (4) 500-ps equilibration at 300 K and 1 atm without the backbone restraint; and (5) 50-ns production at 300 K and 1 atm. Chodera automated equilibration detection scheme^90^ is used to analyze the equilibration statistics in the backend and the results are reported by ResultExplainer in the final Summary page. All MD simulations were performed with a time step of 2 fs with the SHAKE^91^ algorithm applied. The API by default uses the Langevin thermostat^92^ and Berendsen barostat^93^ to control the temperature and pressure, respectively (source code in ref. ^83^). Notably, MutexaGPT does not redistribute proprietary third-party software; backend execution of license-restricted software is available only to users who provide evidence of appropriate authorization, whereas otherwise MutexaGPT outputs scripts and input files for execution in the user’s own licensed environment.

#### Cavity volume calculation

All ensemble cavity volume calculations in this study are performed autonomously on the MutexaGPT server. In the server backend, MutexaGPT calls the ensemble_cavity_volumes() API from EnzyHTP when the cavity volume calculation is needed. The ensemble_cavity_volumes() API allows the upstream calls to specify the target cavity using a list of composing residues, the containing ligand or an existing Cavity() object. For acl-MT, the active site cavity is defined as composed of residues 7, 11, 27, 40, 41, 43, 165, 168, 169, 199, 210, 211 and 289 (SAH). The API then tracks this cavity along the MD trajectory and obtain the cavity mesh as a pyvista.PolyData object^94^, for each frame, using the default, Voronoi-diagram-based, MOLE2 algorithm via the corresponding MOLE2^95^ interface in EnzyHTP. The final cavity volume is obtained through the .volume attribute of the mesh object. For the static structures cavity volumes, we used the same protocol in EnzyHTP but with the static mutant structure as input. These structures are generated by replacing the mutating side chains in the WT structure and search through a rotamer library to find the optimal side-chain position (using the mutate_stru() function in EnzyHTP).

#### DSI calculation

All DSI calculations in this study is performed autonomously on the MutexaGPT server. In the server backend, MutexaGPT calls the dsi() API from EnzyHTP when needed. For each MD trajectory, the dsi() API calls cpptraj to calculate, for each frame, the distance between the center of mass of the designated domains and the radius of gyration of each domain (*R*_g_). Then, DSI = domain distance – (*R*_g,1_ + *R*_g,2_).

#### Configurations of the LLM

All LLM agents used in this study is powered by the OpenAI Python API, specifically, the ‘Responses’ API for the native agentic tool call support from OpenAI. The LLM version is set to gpt-4o-2024-11-20 for the consistent performance besides for ResultExplainer which is set to gpt-5.4 for large context window (temperature 1.00; top *P*: 1.00). Note that users can also choose the LLM model they want to use on the MutexaGPT website (current options include: GPT-4o, GPT-5.4, Qwen3.6 Plus, MiniMax-M2.5, GLM-5, Kimi K2.5, GLM-4.7, Deepseek v3.2, Gemma 4 26B A4B IT and Gemma 4 31B IT). When using the OpenAI models, users need to provide their own OpenAI API keys. Each LLM agent is equipped with tools that allows them to interact with system in the backend and the user experience in the frontend (see design details in Supplementary Section 7 and system prompts in Supplementary Table 1).

### Experimental method

#### Bacterial strains, plasmids and cloning

The DNA sequences for psA, psAt5 and psAt6 were ordered as synthetic genes from Integrated DNA Technologies. Each gene fragment was amplified by polymerase chain reaction with Q5 High-Fidelity Master Mix from New England Biolabs, and the resulting products were inserted into the pET-22b expression vector using Gibson Assembly Master Mix from New England Biolabs. The assembled plasmids were introduced into *Escherichia coli* JM109 for plasmid propagation and into *E. coli* BL21 for subsequent protein expression.

#### Protein expression

Transformed *E. coli* BL21 single colonies were first used to start 5 ml overnight cultures in Luria–Bertani medium containing 50 µg ml^−1^ ampicillin at 37 °C and 220 rpm. A 2-ml portion of each overnight culture was transferred into 200 ml Terrific Broth medium supplemented with the same concentration of ampicillin. The cultures were incubated for 72 h at 30 °C with shaking at 160 rpm. Cells were then pelleted by centrifugation at 10,000*g* for 30 min at 4 °C, and the clarified supernatants were retained as crude enzyme preparations for thermal adaptation assays.

#### Thermal adaptation characterization

Amylase activity was assayed by quantifying reducing sugars generated during starch hydrolysis with the DNSA colorimetric method^71^. For each reaction, 0.2 ml enzyme was mixed with 1.8 ml of 1% (w/v) potato starch (Fisher Scientific) prepared in 15 mM PBS buffer at pH 7.0. Reactions were carried out separately at 0 °C and 45 °C for 15 min, followed by addition of 2 ml DNSA reagent to terminate the reaction. Samples were boiled for 5 min, and absorbance was recorded at 540 nm. One activity unit corresponds to the amount of enzyme producing reducing sugar equivalent to 1 µmol glucose per minute under the assay conditions. Relative activity was calculated as the activity at 0 °C divided by the activity at 45 °C (the optimal temperature of psA), expressed as a percentage. Mean values and standard deviations were calculated from three biologically independent replicates.

### Implementation of MutexaGPT, the workflow and the multi-agent system

At the heart of MutexaGPT is a multi-LLM-agent system that consists of QuestionAnalyzer, WorkPlanningBoard and ResultExplainer (Fig. 2a). They run, in the web-application backend, to co-create intuition-driven requests, supply necessary domain knowledge, convert the request into physical models, configure computational protocols and execute the simulations. The users may begin by providing an intuition-driven request (for example, ‘help me enlarge the active site’) into the system and expect MutexaGPT to execute relevant computational modeling to guide their enzyme engineering tasks. In its well-versed version, these initial, intuition-driven requests should convey three elements: (1) enzymes of interest, (2) an optional, initial mutation library of interest and (3) engineering objectives such as enzyme efficiency, stability and so on. Real requests from users, however, are often vague (for example, the user may just say ‘mutations’ instead of having a specific mutation library in mind), physically inconsistent (for example, ‘improve the temperature of the backbone’ make no sense) or completely off-topic (for example, user may misunderstand the specialization of the webapp and ask it to recommend a protein shake brand).

The QuestionAnalyzer agent steps in and starts a conversation with the user to address these problems: for those irrelevant inquiries, it explains the specialization of the webapp and asks the user to resubmit a new inquiry. For the relevant ones, it actively checks for the missing details, helps the user to articulate implicit assumptions, provides suggestions to reframe scientific terms for clarity and eventually co-creates a scientifically sound and concrete request (see Supplementary Section 1 for an example dialog). The revised question that satisfies QuestionAnalyzer will then be passed to the WorkPlanningBoard, which is composed of two agents, MetricsPlanner and MutantPlanner. The MetricsPlanner maps the engineering objectives of users to a list of physics-based, EnzyHTP^56,57^-computable metrics. For example, a complex target such as ‘drug resistance of mutants’ will be decomposed into a combination of enzyme activity, binding affinity and thermostability, which are then mapped to internal electric field strength, binding free energy and folding energy. MetricsPlanner functions through a conversation with the user, where it will first suggest combinations of metrics, as well as educate the user with necessary domain knowledge explaining the combination. If the user accepts a suggestion, the MetricsPlanner checks whether all mandatory arguments are present, including: selection masks, restraint definitions, protonation states and so on. Any omissions are surfaced as explicit queries, each accompanied by a plausible default. This dialog repeats until every parameter is well defined, at which point the agent compiles a JSON file as the final output in the backend (see Supplementary Section 1 for an example dialog). In parallel to MetricsPlanner, MutantPlanner executes silently in the background and maps the user’s mutation plan into the EnzyHTP mutation pattern, a powerful syntax defined in EnzyHTP that describes a list of mutants each composed of a list of mutations. Combining the output of MetricsPlanner and MutantPlanner gives the final payload JSON object that is ready to be executed by EnzyHTP autonomously. Notably, MutexaGPT allows users to leverage resources from Advanced Computing Center for Research and Education (ACCRE) at Vanderbilt University for the requested computations. The platform also provides users with the option to download a complete folder of prepared input files for all required simulations, together with the exact EnzyHTP Python script and a lightweight configuration file, md_cli_config.json, for adapting the workflow to the syntax of their local HPC environment. Through this configuration file, users can specify site-specific job-management commands, including job submission, status checking, cancellation and the job-script template required by their local queueing system. MutexaGPT then uses this information to generate plain-text submission scripts for each simulation job, which users can inspect and modify before submission if their local queue syntax, module system or software environment requires additional customization. The resulting simulations can be executed using the users’ local computing resources, and the outputs can be uploaded back to MutexaGPT for downstream analysis if needed. To further support reproducibility, particularly when users wish to publish data generated through MutexaGPT, the workflow review page allows users to export the exact EnzyHTP Python script executed by the platform. This script records the full simulation setup, parameters and workflow logic and can, therefore, be directly rerun, archived or cited here or in the Supplementary Information.

Once the simulations are complete, their outputs are collected into a single JSON object that contains (1) the user’s original scientific question, (2) the mean ± standard deviation values for every requested metric, (3) URLs for any downloadable files (trajectory pickles, CSV tables for full results and so on) and (4) metadata of the simulation (force field, length of the MD, equilibration assessment and so on). This JSON becomes the sole input to the terminal agent, ResultExplainer. Acting under the prompt shown in Supplementary Table 2, ResultExplainer handles five top-level goals: (1) translate raw modeling results into concise, domain-relevant takeaways; (2) anchor every explanation to the user’s scientific question rather than to the data in isolation; (3) explain, for each reported metric, what it may indicate biologically or structurally and, equally importantly, what it does not directly measure or prove, thereby reducing the risk of over-interpretation by noncomputational users; (4) communicate the confidence and limitations of the analysis in light of the available simulation context, such as simulation length and equilibration information; and (5) prioritize the most relevant variants and recommend follow-up experiments, additional simulations or data checks (for example, ResultExplainer can rank/filter enzyme variants based on computed metrics; see Supplementary Section 10 for examples of ResultExplainer output). By this final message, MutexaGPT closes the loop from a vague, qualitative intuition to experiment-ready design recommendations with molecular level insights (see Supplementary Section 7 for the design of each individual LLM agent involved in this workflow).

### Implementation of MutexaGPT, the performance of agents

To assess the performance of the three core agents in MutexaGPT (QuestionAnalyzer, WorkPlanningBoard and ResultExplainer), we designed an automated evaluation framework for MutexaGPT (Supplementary Section 8 and Supplementary Fig. 2). This framework leverages LLMs for both generating diverse natural-language test cases (that is, test-set curator agents) and evaluating agent outputs (that is, answer classifier agents). Specifically, a group of test-set curator agents was deployed to create test cases tailored to each core (‘testee’) agent in MutexaGPT, and all generated cases were manually reviewed for accuracy. For evaluation, a set of classifier agents was used to categorize each testee agent’s natural-language output into predefined labels, which were then compared with manually verified ground-truth labels to determine pass/fail outcomes (see Supplementary Section 8 for details).

Using this evaluation framework, we benchmarked the performance of the core agents in MutexaGPT. We focus on QuestionAnalyzer here as a representative example (Fig. 2b) because it is the most sophisticated agent and most directly tied to MutexaGPT’s mission of converting intuition into design. Note that, an LLM agent in MutexaGPT is a LLM (for example, GPT-4o^96^ and so on) configured with a system prompt that instruct and regulate its behavior. In this section, ‘prompt design’ refers specifically to the design of the system prompt that governs the agent’s behavior, rather than the natural-language inquiry/input provided by the user. We therefore benchmarked how different prompt-engineering choices affect agent performance under the same task definition and curated test set. The performance was evaluated against six different prompt designs (Fig. 2b) on the same curated test set of 200 inquiries (100 irrelevant and 100 relevant, with the latter split evenly between fully specified and underspecified cases). The inquiries were generated by the test-set curator and manually reviewed for correctness. Each prompt design was tested across three independent runs, and a design was marked as failing a test if any of the three runs failed.

Evaluation is based on two criteria: relevance (Fig. 2b, top) and completeness (Fig. 2b, bottom). The relevance metric tests whether QuestionAnalyzer correctly classifies user inquiries as relevant or irrelevant. A design is counted as passing only if all three independent runs correctly reject every irrelevant question and accept every relevant one; the reported performance reflects the proportion of designs meeting this standard. The completeness metric assesses whether the agent correctly identifies missing information in underspecified inquiries. We report this metric in three forms: (1) the straight pass ratio, representing designs for which all three runs behave correctly (that is, the agent asks for exactly the information categories that are actually missing in the inquiry: protein, mutation and/or property; orange bars); (2) the double-checking pass ratio, a more permissive measure that also counts cases where the agent correctly identifies missing information but unnecessarily re-asks for existing information—behavior that is overcautious yet harmless to downstream automation (light-blue bars, counted together with orange to form the effective pass rate); and (3) the consensus pass ratio, a relaxed criterion that deems a completeness test successful if at least two of the three runs pass, reflecting tolerance toward stochastic errors (dark-blue bars).

The benchmark results show that the ‘Full’ prompt (Supplementary Table 1), which contains chain-of-thought^97–99^ (CoT; that is, the system prompt contains a list of instructions directing the agent to follow a logic chain) scaffold and seven carefully selected in-context learning (ICL)^100^ examples (that is, examples, as input and output pairs, are provided in the system prompt, also known as few-shot learning), emerges as the best performing design. It accepts or rejects queries correctly in 97.0% of cases, achieves an 71.0% strict completeness pass rate and climbs to 97.0% once double-checks are counted; its consensus robustness stands at 94.0%. Removing the ICL snippets (‘No ICL’) leaves the relevance pass rate almost unchanged (99.0%) but cuts the strict completeness pass rate to 41.0%, because the model becomes uncertain and compensates with many redundant queries (54.0% in double-check rate). Suppressing the explicit reasoning scaffold instead (‘No CoT’) has a similar although slightly less severe effect: strict completeness pass falls to 45.0%. The ‘Only ICL’ prompt, which only contains the seven examples and a single-sentence role description, recovers some ground (58.0% strict completeness pass rate) yet remains less stable. Augmenting the ‘Full’ prompt with a one-line self-reflection instruction (‘Full + self-check’) did not translate into a net win. Although the effective pass rate remains high (98.0%), the strict completeness pass rate falls by roughly eight percentage points and the proportion of redundant double-checks grows, as well as pulling the consensus robustness down from 94.0% to 91.0%. In other words, the extra self-check mostly prompts the agent to question information that is already complete, offering little practical benefit while adding conversational noise. At the opposite extreme, a minimalist specification (‘Simplistic’), which only contains a sentence of role description and the output format in the system prompt, still rejects irrelevant questions (96.5%) but collapses on strict completeness (16.0%), succeeding in barely half the cases even though the looser consensus pass is measured (54.0%). Error analysis of the result of the Full QuestionAnalyzer shows that relevance failures are exclusively false negatives, primarily arising from overly vague inquiry (for example, ‘What effect do ligands have on proteins?’), while effective-pass completeness failures are rare (3/100) and mostly associated with marginally ambiguous property definitions (for example: ‘flexibility of …’) rather than systematic inability to identify incomplete inquires.

Three design lessons follow. First, CoT and ICL contribute independently and additively to the agent’s ability to solicit precisely the missing information, removing either one reduces the performance by 25–30 percentage points. Second, a cheap self-check clause provides an easy safety net against occasional lapses. Third, a blanket ‘self-check before replying’ clause is not an automatic upgrade. In our hands, it lowered strict completeness and inflated redundant follow-ups. Third, minimal prompts are insufficient for complex reasoning. The Simplistic design, removing CoT and examples, collapsed to one-third completeness pass rate and showed the highest rate of redundant clarifications, underscoring the necessity of structured guidance for complex tasks (for example, CoT, ICL and so on). Without them, the model defaults to hesitation and repetitive questioning instead of reliable information gathering. Note that, while the ‘Full’ prompt design performs better in our context, a richer system prompt carries a potential downside: it not only improves in-domain precision by constraining the agent but also reduces flexibility on atypical or out-of-distribution requests by anchoring behavior to the included examples and interaction schema^101,102^.

Besides QuestionAnalyzer, every agent in the MutexaGPT workflow is subjected to its own benchmark similar to the one described above, albeit with a single, production-ready prompt rather than the full suite of design variants explored above (Supplementary Section 8). For MetricsPlanner, the test-set curator generates inputs (that match the format of the QuestionAnalyzer’s final output, with 100 cases asking about properties that can map to one of our supported metrics and 100 that are not) from ground-truth metric/MD-model labels, and a case passes only when the agent’s recommended candidates contain the labeled metric-model pair or inform user when none of the supported metrics can correspond to the inquiry. For MutantPlanner, curator-agent generated inputs contain a balanced set of unrestricted, pocket, protein–protein interface, distal (relative to the pocket) mutational libraries and designated site mutations (10 for each, 50 in total). The correctness is judged by whether the generated EnzyHTP mutation pattern is semantically equivalent to the labeled library (that is, they are parsed by EnzyHTP and directly compared for equivalence). For ResultExplainer, a set of JSON payloads are curated by a curator agent (containing ‘scientific question’, ‘metrics’, ‘results’, ‘downloadables’ and ‘metadata’; 100 in total). The outputs of ResultExplainer are evaluated by two judge agents, one assessing how well the output aligns to the scientific question from the input and the other assessing interpretation-boundary discipline (that is, avoidance of overclaiming beyond the provided data), and overall pass requires satisfying both criteria. Full benchmark inputs, pass definitions and per-run outputs are provided in Supplementary Section 8 and SI.zip/agent_evaluation.

The results show that the prompt design for each agent is highly effective in their respective roles: for WorkPlanningBoard, MetricsPlanner on average passes 91% of its test cases, MutantPlanner achieves a 95% success rate; ResultExplainer met all evaluation criteria in 99% of the test cases. While not directly comparable, these performance levels are strong in the broader context of LLM benchmarks for scientific tasks (for reference, recent scientific-discovery benchmarks^103^ typically report only 0.60–0.75 question-level accuracy across domains, while the best model in a question-clarification benchmark^104^ reaches a maximum success rate of 60.1%). One can find details in Supplementary Section 9 for error analysis of each agent, and SI.zip for spreadsheets that contain the test input, ground-truth label, agent’s output, the judge agent’s output and the pass/failure of every test case of each agent in MutexaGPT.

### Reporting summary

Further information on research design is available in the Nature Portfolio Reporting Summary linked to this article.

## Supplementary information

Supplementary Information Supplementary Sections 1–13, Figs. 1–10, Tables 1–4 and references.

Reporting Summary

Peer Review File

Supplementary Table 1 System prompts of all LLM agents involved in this study.

Supplementary Table 2 Full sequences of psA variants.

## Source data

Source Data Fig. 2 Raw data used to plot Fig. 2.

Source Data Fig. 3 Raw data used to plot Fig. 3.

Source Data Fig. 4 Raw data used to plot Fig. 4.

## Data Availability

Source data for all figures are available with this paper. The following source data are provided in the Supplementary information: Example dialog between user and MutexaGPT for setting up the cavity engineering study; Example dialog between user and MutexaGPT for setting up the cold-adaption study; Example dialog between user and MutexaGPT for setting up the non-native activity engineering study; Example dialog between user and MutexaGPT for setting up the cavity engineering study using Kimi K2.5; Example dialog between user and MutexaGPT for setting up the cold-adaption study using Kimi K2.5; Example dialog between user and MutexaGPT for setting up the non-native activity engineering study using Kimi K2.5; The design of each individual LLM-agent; The design logics of LLM-agents in MutexaGPT; The evaluation system of LLM-agents; The evaluation system of LLM-agents; Failure mode analysis in the agent performance evaluation; Example ResultExplainer output and Summary page screenshot for each use case.; Screenshots of the Summary page for use case 1: Cavity Engineering; Screenshots of the Summary page for use case 2: Cold Adaptation Engineering; Screenshots of the Summary page for use case 3: Non-native Enzyme Activity Engineering; Example ResultExplainer output and Summary page screenshot for each use case. (model = Kimi K2.5); Screenshots of the Summary page for use case 1: Cavity Engineering using Kimi K2.5; Screenshots of the Summary page for use case 2: Cold Adaptation Engineering using Kimi K2.5; Screenshots of the Summary page for use case 3: Non-native Enzyme Activity Engineering using Kimi K2.5; The hit rate baseline of the *A. clavatus* methyltransferase library used in this study; The cumulative hit curve for use case 1: Cavity Engineering; Metrics MutexaGPT currently supports; Topology-based selection of psA linkers; Linker sequences used in cold-adaption engineering and their calculated DSI; The DSI distribution of the psA WT type and its ten linker variants; Amylase activity of psA WT, psAt5, and psAt6 under 0 °C and 45 °C; Parameters used for structure prediction using ColabFold server. The original input file for all involved MD simulations and full LLM-agent evaluation results are available via Zenodo at https://doi.org/10.5281/zenodo.21197440 (ref. ^105^).

## Code availability

The MutexaGPT webapp is available at https://enzyhtp.app.vanderbilt.edu/. The source code of MutexaGPT and the evaluation system of LLM agents in MutexaGPT is available via GitHub at https://github.com/ChemBioHTP/EnzyHTP-GPT/ and via Zendodo at https://doi.org/10.5281/zenodo.21197663 (ref. ^106^). OpenAI Codex was used as an AI coding assistant during the development of MutexaGPT, and all final code was reviewed and curated by the authors. EnzyHTP is available via GitHub at https://github.com/ChemBioHTP/EnzyHTP. AMBER is available from http://ambermd.org/.
