# Supplementary material for: MutexaGPT: an intuition-to-design translator for physics-based enzyme engineering
Source: Nat Comput Sci. 2026 Sep 10;6(10):1133–46. doi: 10.1038/s43588-026-01049-y (PMC13686939; doi:10.1038/s43588-026-01049-y)
Supplement: Supplementary file 1 [file 43588_2026_1049_MOESM1_ESM.pdf]

nature computational science

Supplementary information

https://doi.org/10.1038/s43588-026-01049-y

MutexaGPT: an intuition-to-design translator for physics-based enzyme engineering

In the format provided by the authors and unedited

Contents

Supplementary Section 1. Example dialog between user and MutexaGPT for setting up the cavity engineering study Page S3
Supplementary Section 2. Example dialog between user and MutexaGPT for setting up the cold-adaption study Page S5
Supplementary Section 3. Example dialog between user and MutexaGPT for setting up the non-native activity engineering study Page S7
Supplementary Section 4. Example dialog between user and MutexaGPT for setting up the cavity engineering study using Kimi K2.5 Page S10
Supplementary Section 5. Example dialog between user and MutexaGPT for setting up the cold-adaption study using Kimi K2.5 Page S12
Supplementary Section 6. Example dialog between user and MutexaGPT for setting up the non-native activity engineering study using Kimi K2.5 Page S15
Supplementary Section 7. The design of each individual LLM-agent Page S17
Supplementary Figure 1. The design logics of LLM agents in MutexaGPT Page S18
Supplementary Section 8. The evaluation system of LLM-agents Page S19
Supplementary Figure 2. The evaluation system of LLM-agents Page S19
Supplementary Section 9. Failure mode analysis in the agent performance evaluation Page S20
Supplementary Section 10. Example ResultExplainer output and Summary page screenshot for each use case. Page S22
Supplementary Figure 3. Screenshots of the Summary page for use case 1: Cavity Engineering Page S25
Supplementary Figure 4. Screenshots of the Summary page for use case 2: Cold Adaptation Engineering Page S28
Supplementary Figure 5. Screenshots of the Summary page for use case 3: Non-native Enzyme Activity Engineering Page S31
Supplementary Section 11. Example ResultExplainer output and Summary page screenshot for each use case. (model = Kimi K2.5) Page S31
Supplementary Figure 6. Screenshots of the Summary page for use case 1: Cavity Engineering using Kimi K2.5 Page S34
Supplementary Figure 7. Screenshots of the Summary page for use case 2: Cold Adaptation Engineering using Kimi K2.5 Page S37
Supplementary Figure 8. Screenshots of the Summary page for use case 3: Non-native Enzyme Activity Engineering using Kimi K2.5 Page S40
Supplementary Section 12. The hit rate baseline of the *Aspergillus clavatus* methyltransferase library used in this study Page S41
Supplementary Figure 9. The cumulative hit curve for use case 1: Cavity Engineering Page S41
Supplementary Table 1. Metrics MutexaGPT currently supports Page S41
Supplementary Section 13. Topology-based selection of psA linkers Page S42
Supplementary Table 2. Linker sequences used in cold-adaption engineering and their calculated Domain Separation Index (DSI) Page S42

Supplementary Figure 10. The DSI distribution of the *Pseudomonas saccharophila* amylase wild type and its ten linker variants Page S43
Supplementary Table 3. Amylase activity of psA wild-type, psAt5, and psAt6 under 0 °C and 45 °C Page S43
Supplementary Table 4. Parameters used for structure prediction using ColabFold server Page S43
Reference Page S44

Supplementary Section 1. Example dialog between user and MutexaGPT for setting up the cavity engineering study.

MutexaGPT:

Hi, Qianzhen Shao how can I help you today?

User:

Help me find mutations that make the active site cavity larger.

MutexaGPT:

- **Target Protein**: Could you specify which protein you are interested in studying? (Suggestion: Please upload a PDB file of the wild-type protein structure.)
- **Mutations**: Could you specify the exact mutation library you are interested in? (Suggestion: Do you want to study 'site-saturation single-point mutations for residue sites within 4 angstroms of the substrate'?)
- **Property of Interest**: Could you confirm what you mean by "make the active site cavity larger"? Are you referring to the cavity size or another related property? (Suggestion: Do you want to study 'the cavity size of the active site pocket'?)

User:

I want to study a halide methyltransferase acl-MT. For the mutations, I want to study a combinatorial mutation library that mutates residue 11 to A, F, I, or L; residue 30 to C, F, I, or M; residue 31 to W; residue 39 to H, D, I, E, or T; residue 41 to F. Each point can either be mutated or not mutated.

MutexaGPT:

Thank you for providing the details.

- **Target Protein**: You mentioned halide methyltransferase acl-MT. Could you upload a PDB file of the wild-type structure for acl-MT? (Suggestion: Please upload the wild-type PDB file of acl-MT.)
- **Mutations**: The combinatorial mutation library is clear. Thank you.
- **Property of Interest**: Just to confirm, by "make the active site cavity larger," you are specifically referring to studying the cavity size of the active site pocket. Is that correct?

User:

I have uploaded the wild-type acl-MT. Studying the cavity size sounds good!

MutexaGPT:

Target property: cavity size of the active site pocket.

Here are the relevant computational metrics we currently support:

1. Cavity Volume (modeled as the apo-protein without constraint)

Please confirm which one you want to use.

Suggestion: #1

Reason: The "Cavity Volume" metric aligned with the apo-protein will directly determine the cavity size of the active site pocket, which matches your property of interest effectively.

User:

Sounds good!

MutexaGPT:

I found the following missing information for these metrics:

1. Cavity Volume (modeled as the "apo-protein" without constraint)
   pocket_compositing_residue_pattern : the PyMol style selection pattern defining

the residues composing the active site pocket.
(Suggestion: You may define the residues of the pocket using residue IDs based on your understanding of the pocket structure in aclMT-SAH.pdb. For instance, selecting residues within 5 angstroms around a center substrate atom could be a useful approximation for active-site pockets.)

These pieces of information define the question you want to study. Please be careful and let me know your choice. You can directly identify them following the EnzyHTP syntax or describe them using natural language.

User:

The cavity is composed by residues: "289", "7", "11", "27", "40", "41", "43", "165", "168", "169", "199", "210", "211"

MutexaGPT:

Great, here is the compiled information for the target computational metrics:


```
{
    "metrics": [
        {
            "name": "cavity",
            "arguments": {
                "pocket_compositing_residue_pattern": "resi 289+7+11+27+40+41+43+165+168+169+199+210+211"
            }
        }
    ],
    "constraints": []
}
```


MutexaGPT:

Please confirm the mutations you want:

a:[resi 11:ALA or PHE or ILE or LEU, resi 30:CYS or PHE or ILE or MET, resi 31:TRP, resi 39:HIS or ASP or ILE or GLU or THR, resi 41:PHE]

This mutation pattern defines a combinatorial mutation library where each residue can have up to one mutation at a time. The positions included in this library are residues 11, 30, 31, 39, and 41. At residue 11, the allowed substitutions are alanine (ALA), phenylalanine (PHE), isoleucine (ILE), or leucine (LEU). At residue 30, the options are cysteine (CYS), phenylalanine (PHE), isoleucine (ILE), or methionine (MET). For residue 31, the only substitution is tryptophan (TRP). At residue 39, the substitutions include histidine (HIS), aspartate (ASP), isoleucine (ILE), glutamate (GLU), or threonine (THR). Finally, at residue 41, the substitution allowed is phenylalanine (PHE). The library will include all possible single-site substitution combinations from these specified residues and options.

User:

Looks good.

MutexaGPT:

Great, you may now proceed with the next step in the workflow!

Supplementary Section 2. Example dialog between user and MutexaGPT for setting up the cold-adaption study.

MutexaGPT:

Hi, Qianzhen Shao how can I help you today?

User:

Hi! I am studying a bidomain enzyme. This enzyme showed cold-adaption behavior, that is, the catalytic activity reduces much slower in lower temperatures. We found that just by changing the linker, the cold-adaption changes, can you help me perform some modeling to find linkers that provide stronger cold-adaption?

MutexaGPT:

- **Target Protein**: Could you specify which bidomain enzyme you are studying? Please upload the PDB file of the enzyme, particularly one in the cold-adaptive state or wild-type structure. (Suggestion: Use the uploaded PDB file if available.)
- **Mutations**: Could you clarify if you want to study mutations in the linker region? If yes, please specify if it's random mutations, specific substitutions, or insertion/deletion modifications in the linker. (Suggestion: Provide how you would like to study linker variations. For instance, testing random mutations or specific linker sequences.)
- **Property of Interest**: Finding stronger cold-adaption is a bit broad. Do you want to focus on specific properties such as aligned thermostability or catalytic efficiency at low temperatures? (Suggestion: Specify if you want to model "catalytic efficiency at low temperatures" for defining cold-adaption.)

User:

I have uploaded a zip file that contains psA wild-type and 10 psA variants with different linker sequences.

MutexaGPT:

- **Target Protein**: The uploaded zip file "initial_stru.zip" contains the psA wild-type and 10 psA variants with different linker sequences.
- **Mutations**: Do you want to study all 10 variants to model the effect of their unique linker sequences? If not, please specify whether you have a different selection of linker variations to explore. Additionally, clarify if experimenting with new linker designs is also a part of your goal. (Suggestion: Confirm if all existing variants will be included. If interested in new designs, specify details like numerical sampling or sequence selection.)
- **Property of Interest**: Could you confirm whether the property of interest is "catalytic efficiency at low temperatures" specifically? This clarification will guide better modeling toward stronger cold-adaption. (Suggestion: Confirm or clarify whether "improving catalytic efficiency at lower temperatures" is the studied property of cold-adaption.)

User:

Yes, I want to study all 10 variants to model the effect of their unique linker sequences.

MutexaGPT:

- **Target Protein**: The uploaded zip file "initial_stru.zip" contains the psA wild-type and 10 psA variants with different linker sequences.

- **Mutations**: All 10 psA variants with unique linker sequences from the uploaded file will be studied.
- **Property of Interest**: Could you confirm whether the property of interest is "catalytic efficiency at low temperatures" specifically? This will precisely define the cold-adaptation target for the modeling. (Suggestion: Confirm or clarify whether "improving catalytic efficiency at lower temperatures" is the studied property of cold-adaption.)

User:

The cold-adaption is defined as the relative activity at 0°C relative to that at 45°C

MutexaGPT:

Target property: Cold-adaptation

Here are the relevant computational metrics we currently support:

1. Domain Separation Index (DSI) (modeled as the apo-protein without constraint)

Please confirm whether you want to use the Domain Separation Index (DSI) metric.

Suggestion: #1

Reason: The Domain Separation Index (DSI) metric is shown to correlate with the cold-adaptation ability of bi-domain enzymes, making it directly relevant to your property of interest.

User:

Sounds good!

MutexaGPT:

I found the following missing information for these metrics:

1. Domain Separation Index (DSI) (modeled as the apo-protein without constraint)
    - domain1_sequence: The sequence of the first domain. The backend will locate this exact sequence in the chain to determine the sequence range of the first domain. (Suggestion: Provide the sequence of one of the psA protein domains in FASTA format.)
    - domain2_sequence: The sequence of the second domain. The backend will locate this exact sequence in the chain to determine the sequence range of the second domain. (Suggestion: Provide the sequence of the other domain of the psA protein in FASTA format.)

These pieces of information define the domains you wish to study for their separation index. Please be careful and let me know your input. You can directly identify them following the EnzyHTP syntax or describe them using natural language.

User:

domain 1:
DQAGKSPAGVRYHGGDEIILQGFHWNVVREAPNDWYNILRQQASTIAADGFSAI
WMPVPWRDFSSWTDGGKSGGGEGYFWHDFNKNGRYGSDAQLRQAAGALGGA
GVKVLYDVVPNHMNRGYPDKEINLPAGQGFWRNDCADPGNYPNDCDDGDRFI
GGESDLNTGHPQIYGMFRDELANLRSGYGAGGFRFDFVRGYAPERVDSWMSDS
ADSSFCVGELWKGPSEYPSWDWRNTASWQQIIKDWSDRAKCPVFDFALKERMQ
NGSVADWKHGLNGNPDPRWREVAVTFVDNHDTGYSPGQNGGQHHWALQDGL
IRQAYAYILTSPGTPVVYWSHMYDWGYGDFIRQLIQVRRTAGVRADSAISFHSG
YSGLVATVSGSQQTLVVALNSDLANPGQVASGSFSEAVNASNGQVRVWRS

domain 2:

VNVNFRCDNGVTQMGDSVYAVGNVSQLGNWSPASAVRLTDTSSYPTWKGSIA
LPDGQNVEWKCLIRNEADATLVRQWQSGGNNQVQAAAGASTSGSF

MutexaGPT:

Great, here is the compiled information for the target computational metrics:


```
{
    "metrics": [
        {
            "name": "dsi",
            "arguments": {
                "domain1_sequence":
"DQAGKSPAGVRYHGGDEIILQGFHWNVVREAPNDWYNILRQQASTIAADGFSA
IWMPVPWRDFSSWTDGGKSGGGEGYFWHDFNKNGRYGSDAQLRQAAGALGG
AGVKVLYDVVPNHMNRGYPDKEINLPAGQGFWRNDCADPGNYPNDCDDGDRF
IGGESDLNTGHPQIYGMFRDELANLRSGYGAGGFRFDFVRGYAPERVDSWMSD
SADSSFCVGELWKGPSEYPSWDWRNTASWQQIIKDWSDRAKCPVFDFALKERM
QNGSVADWKHGLNGNPDPRWREVAVTFVDNHDTGYSPGQNGGQHHWALQDG
LIRQAYAYILTSPGTPVVYWSHMYDWGYGDFIRQLIQVRRTAGVRADSAISFHS
GYSGLVATVSGSQQTLVVALNSDLANPGQVASGSFSEAVNASNGQVRVWRS",
                "domain2_sequence":
"VNVNFRCDNGVTQMGDSVYAVGNVSQLGNWSPASAVRLTDTSSYPTWKGSIA
LPDGQNVEWKCLIRNEADATLVRQWQSGGNNQVQAAAGASTSGSF"
            }
        }
    ],
    "constraints": []
}
```


MutexaGPT:

Please confirm the mutations you want:

WT

This mutation pattern corresponds to the wild-type, meaning no mutations have been introduced. It represents the natural, unaltered sequence of the protein.

User:

Looks good

MutexaGPT:

Great, you may now proceed with the next step in the workflow!

Supplementary Section 3. Example dialog between user and MutexaGPT for setting up the non-native activity engineering study.

User:

Help me find mutations that make my enzyme faster for an non-native substrate ethyl iodide.

MutexaGPT:

- **Target Protein**: Could you specify which enzyme you are studying by uploading its structure or providing additional information? Is the uploaded file in the wild-type state? (Suggestion: Please upload a PDB file of your enzyme's wild-type structure.)
- **Mutations**: Could you specify what type of mutations you are interested in? (Suggestion: Do you want to explore random mutations, site-directed mutations, or site-saturation across specific sites?)
- **Property of Interest**: Could you clarify what "faster" means in this context? Are you referring to catalytic efficiency, specifically changes in turnover number (kcat) or catalytic efficiency (kcat/KM), for the non-native substrate ethyl iodide? (Suggestion: Do you want to investigate "kcat/KM for ethyl iodide"?)

User:

I have uploaded the wild-type atHMT in complex with the non-native substrate ethyl iodide (ETI). For mutations, I want to explore all single point mutations with in 4A of ETI. For property, it will be the catalytic efficiency of ETI.

MutexaGPT:

- **Target Protein**: the uploaded wild-type atHMT enzyme in complex with the non-native substrate ethyl iodide (ETI) at athmt-eti.pdb.
- **Mutations**: all single-point mutations within 4 angstroms of ETI.
- **Property of Interest**: catalytic efficiency for ethyl iodide.

MutexaGPT:

Target property: catalytic efficiency for ethyl iodide

Catalytic efficiency is a combination of enzyme activity and substrate binding. Upon decomposition:

- **Enzyme activity** can be broken down further into the activation barrier (related to electric field and substrate positioning dynamics) and thermostability (when mutations are involved). Since mutations are in proximity to the active site, thermostability may play a role.
- **Substrate binding** is directly associated with ligand binding metrics.

Here are the relevant computational metrics we currently support:

1. **Electric Field Strength** (modeled as the "pre-reaction complex," with a constraint applied to the forming bond).
2. **Substrate Positioning Index (SPI)** (modeled as the "pre-reaction complex," with a constraint applied to the forming bond).
3. **MMPB/GBSA Ligand Binding Energy** (modeled as the protein-ligand complex without constraint).
4. **Relative Folding Free Energy** (not dependent on any MD model).

Please confirm which one(s) you want to use.

Suggestion: #1 and #4 in combination.

Reason: These metrics together address catalytic efficiency as they measure the electric field's role in reducing the activation barrier for catalysis and thermostability. In the meantime, this combination is most cost effective.

User:

The combination of 1 and 4 sounds good.

MutexaGPT:

I found the following missing information for these metrics:

1. **Electric Field Strength** (modeled as the "pre-reaction complex," with a constraint applied to the forming bond):
    - **atom_1**: the first atom of the forming bond involved in the catalysis (Suggestion: Specify the atom name, residue index, and chain for the substrate ETI and the enzyme active site).
    - **atom_2**: the second atom of the forming bond involved in the catalysis (Suggestion: Specify the atom name, residue index, and chain for the substrate ETI and the enzyme active site).
2. **Relative Folding Free Energy**:
    - No missing information, as no arguments are required for this metric.

These details are critical for accurately defining the system of study. Please provide the required information. You can directly use the EnzyHTP syntax or describe it in natural language.

User:

They are C.218.C1 and C.218.I1

MutexaGPT:

Great, here is the compiled information for the target computational metrics:


```
{
    "metrics": [
        {
            "name": "electric_field",
            "arguments": {
                "atom_1": "C.218.C1",
                "atom_2": "C.218.I1"
            }
        },
        {
            "name": "ddg_fold",
            "arguments": {}
        }
    ],
    "constraints": []
}
```


MutexaGPT:

Please confirm the mutations you want:

{a:1[resn ETI around 4:all not self]}

This mutation pattern describes a combinatorial mutant library where every mutant contains exactly one point mutation. The possible mutation sites are any residue within 4 Å of residues named ETI, and at each of these sites, the residue is mutated to any of the 20 canonical amino acids except the original one.

User:

Looks good

Supplementary Section 4. Example dialog between user and MutexaGPT for setting up the cavity engineering study using Kimi K2.5.

User:

Help me find mutations that make the active site cavity larger.

MutexaGPT:

I can help you find mutations that affect the active site cavity size! Let me analyze your question:

- **Target Protein**: Could you specify which protein you are interested in studying? You'll need to upload the PDB file in the box above. (Suggestion: Please upload a PDB file of the wild-type protein structure)
- **Mutations**: Could you specify the exact library of mutations you are interested in exploring? (Suggestion: Do you want to study 'site-saturation single-point mutations for residue sites that form the active site'? Or perhaps 'random mutations in the active site region'?)
- **Property of Interest**: You want to study how mutations affect the active site cavity. Please confirm that you're interested in the cavity volume/size? (Suggestion: Do you want to study 'the cavity size/volume of the active site binding pocket'? Also, does your uploaded structure contain a ligand/substrate in the active site that defines the region of interest?)

User:

I want to study a halide methyltransferase acl-MT. (uploaded as wild-type & SAH holo-complex pdb file) For the mutations, I want to study a combinatorial mutation library that mutates residue 11 to A, F, I, or L; residue 30 to C, F, I, or M; reisude 31 to W; residue 39 to H, D, I, E, or T; residue 41 to F. Each point can either be mutated or not mutated.

MutexaGPT:

Final Output:

- **Target Protein**: the uploaded wild-type & SAH holo-complex acl-MT PDB file at aclHMT-SAH_rmW_rmH_aH.pdb
- **Mutations**: combinatorial mutation library: residue 11 (A, F, I, L or WT), residue 30 (C, F, I, M or WT), residue 31 (W or WT), residue 39 (H, D, I, E, T or WT), residue 41 (F or WT)
- **Property of Interest**: the cavity size of the active site binding pocket

---

Does this accurately capture your question? If so, the next agent will proceed with the simulation workflow to find mutations that increase the active site cavity size!

MutexaGPT:

Target property: cavity size of the active site binding pocket

The property "cavity size" is a fundamental property that does not require decomposition.

Here are the relevant computational metrics we currently support:

1. **Cavity Volume** (cavity) - The cavity volume of a binding pocket of the protein

Please confirm which one you want to use.

Suggestion: #1 *Reason*: The Cavity Volume metric directly measures the size of the binding pocket, which aligns perfectly with your property of interest.

User:

Looks good

MutexaGPT:

I found the following missing information for these metrics:

1. **Cavity Volume** (cavity)
   - **ligand_selection_pattern** OR **pocket_compositing_residue_pattern**: You need to define either the ligand contained in the cavity (which determines the pocket) OR the residues that compose the pocket. (Suggestion: Since you mentioned an "SAH holo-complex", SAH (S-adenosylhomocysteine) is likely the ligand in the active site. You could use resn SAH to select it by residue name, or if you know the specific residues forming the pocket, you could specify them, e.g., resi 11+30+31+39+41 around 5 to capture residues near your mutation sites)

These information defines the question you want to study. Please be careful and let me know your choice. You can directly identify them following the EnzyHTP syntax or describe them using natural language.

User:

The cavity is composed by residues: "B.289", "A.7", "A.11", "A.27", "A.40", "A.41", "A.43", "A.165", "A.168", "A.169", "A.199", "A.210", "A.211"

MutexaGPT:

Great, here is the compiled information for the target computational metrics:


```
{
    "metrics" : [
        {
            "name" : "cavity",
            "arguments" : {
                "pocket_compositing_residue_pattern" : "(chain A and resi 7+11+27+40+41+43+165+168+169+199+210+211) or (chain B and resi 289)"
            }
        }
    ],
    "constraints" : []
}
```


Summary of your configuration:

- **Metric**: Cavity Volume - measures the volume of the active site binding pocket
- **Pocket Definition**: 13 residues composing the cavity across chains A and B
  - Chain A: residues 7, 11, 27, 40, 41, 43, 165, 168, 169, 199, 210, 211
  - Chain B: residue 289
- **No Constraints**: Cavity volume is calculated on the apo-protein/holo-complex without requiring any geometric constraints

This setup will calculate the cavity size of the active site pocket for each mutant in your combinatorial library (residues 11, 30, 31, 39, 41) to identify mutations that increase the active site cavity volume!

MutexaGPT:

Please confirm the mutations you want:

a:[resi 11:ALA or PHE or ILE or LEU, resi 30:CYS or PHE or ILE or MET, resi 31:TRP, resi 39:HIS or ASP or ILE or GLU or THR, resi 41:PHE]

This pattern defines a **combinatorial single-point mutation library** (indicated by the a: prefix) in which each mutant contains **at most one** amino-acid substitution (the number of mutations is omitted, defaulting to 1). The library spans five specific positions and includes the wild-type enzyme plus every possible single mutant drawn from the following sets: residue 11 can be changed to Alanine, Phenylalanine, Isoleucine, or Leucine; residue 30 to Cysteine, Phenylalanine, Isoleucine, or Methionine; residue 31 to Tryptophan; residue 39 to Histidine, Aspartate, Isoleucine, Glutamate, or Threonine; and residue 41 to Phenylalanine. Because the force-mutation flag "M" is absent, the wild-type (no mutation) is also included in the scheme.

User:

Looks good

Supplementary Section 5. Example dialog between user and MutexaGPT for setting up the cavity engineering study using Kimi K2.5.

User:

I am studying a bidomain enzyme. This enzyme showed cold-adaption behavior, that is, the catalytic activity reduces much slower in lower temperatures. We found that just by changing the linker, the cold-adaption changes, can you help me perform some modeling to find linkers that provide stronger cold-adaption?

MutexaGPT:

I need some clarification to help you study the cold-adaptation of your bidomain enzyme through modeling.

- **Target Protein**: I could not find the uploaded structure file for your bidomain enzyme. Could you please upload the PDB structure file in the upload box? (Suggestion: Please upload the wild-type PDB file of your bidomain enzyme, ideally including the linker region that connects the two domains)
- **Mutations**: You mentioned "changing the linker", but could you specify exactly what linker mutations or variants you want to explore? (Suggestion: Do you want to study specific linker sequences (e.g., 5-10 different linker variants), or random mutations within the linker region, or do you need computational suggestions for linker designs? Also, what is the current linker sequence or residue range in the wild-type structure?)
- **Property of Interest**: You mentioned "cold-adaptation" as reduced loss of catalytic activity at lower temperatures. This is clear, but do you want to compare activity at a specific low temperature vs optimal temperature, or study the temperature-activity profile? (Suggestion: Do you want to study "the relative catalytic activity at a lower temperature (e.g., 4°C/277K) compared to optimal temperature"? Or perhaps "the activation energy or thermal stability related to catalytic activity"?)

User:

I have uploaded a zip file that contains psA wild-type and 10 psA variants with different linker sequences. No mutations needed. The cold-adaption is defined as the relative activity at 0°C relative to that at 45°C

MutexaGPT:

Perfect! I have all the information needed. Here is the final analysis:

Final Output:

- **Target Protein**: the uploaded zip file (initial_stru.zip) containing the psA wild-type structure and 10 psA variants with different linker sequences
- **Mutations**: N/A (the linker variants are already provided in the uploaded files)
- **Property of Interest**: the relative catalytic activity at 0°C (273K) relative to that at 45°C (318K) as a measure of cold-adaptation, where stronger cold-adaptation means higher relative activity at low temperature

---

This setup will allow us to computationally screen the 10 linker variants against the wild-type to identify which linkers provide stronger cold-adaptation (higher relative activity at 0°C vs 45°C).

I will now proceed to the next agent to begin the computational workflow.

MutexaGPT:

Target property: Relative catalytic activity at 0°C relative to 45°C (cold-adaptation ability)

Your target property describes the ability of a bidomain enzyme to maintain activity at lower temperatures relative to higher temperatures, which is a measure of cold-adaptation ability.

Here are the relevant computational metrics we currently support:

1. Domain Seperation Index (DSI) (modeled as the apo-protein without constraint)
2. Domain Seperation Index (DSI) (modeled as the protein-ligand complex without constraint)
3. Domain Seperation Index (DSI) (modeled as the pre-reaction complex with a constraint applied to the forming bond)

Please confirm which one you want to use.

Suggestion: #1 *Reason*: The Domain Seperation Index (DSI) is specifically designed to predict cold-adaptation ability in bidomain enzymes by measuring how separate the two domains are in their dynamic motion. This metric requires the apo-protein conformation (without ligand or constraints) to properly capture the inherent domain dynamics. This aligns perfectly with your property of interest in measuring relative activity at 0°C vs 45°C for the psA bidomain enzyme variants with different linker sequences.

User:

Sounds good

MutexaGPT:

I found the following missing information for these metrics:

1. Domain Seperation Index (DSI) (modeled as the apo-protein without constraint) domain1_sequence : The sequence of the first domain. The backend will locate this exact sequence in the chain to determine the sequence range of the first domain. (Suggestion: Please provide the amino acid sequence of the first domain of psA)

domain2_sequence : The sequence of the second domain. The backend will locate this exact sequence in the chain to determine the sequence range of the second domain. (Suggestion: Please provide the amino acid sequence of the second domain of psA)

These information defines the question you want to study. Please be careful and let me know your choice. You can directly identify them following the EnzyHTP syntax or describe them using natural language.

User:

domain 1: DQAGKSPAGVRYHGGDEIILQGFHWNVVREAPNDWYNILRQQASTIAADGFSAIWMPVPWRDFSSWTDGGKSGGGEGYFWHDFNKNGRYGSDAQLRQAAGALGGAGVKVLYDVVPNHMNRGYPDKEINLPAGQGFWRNDCADPGNYPNDCDDGDRFIGGESDLNTGHPQIYGMFRDELANLRSGYGAGGFRFDFVRGYAPERVDSWMSDSADSSFCVGELWKGPSEYPSWDWRNTASWQQIIKDWSDRAKCPVFDFALKERMQNGSVADWKHGLNGNPDPRWREVAVTFVDNHDTGYSPGQNGGQHHWALQDGLIRQAYAYILTSPGTPVVYWSHMYDWGYGDFIRQLIQVRRTAGVRADSAISFHSGYSGLVATVSGSQQTLVVALNSDLANPGQVASGSFSEAVNASNGQVRVWRS

domain 2: VNVNFRCDNGVTQMGDSVYAVGNVSQLGNWSPASAVRLTDTSSYPTWKGSIALPDGQNVEWKCLIRNEADATLVRQWQSGGNNQVQAAAGASTSGSF

MutexaGPT:

Great, here is the compiled information for the target computational metrics:


```
{
    "metrics" : [
        {
            "name" : "dsi",
            "arguments" : {
                "domain1_sequence" : "DQAGKSPAGVRYHGGDEIILQGFHWNVVREAPNDWYNILRQQASTIAADGFSAIWMPVPWRDFSSWTDGGKSGGGEGYFWHDFNKNGRYGSDAQLRQAAGALGGAGVKVLYDVVPNHMNRGYPDKEINLPAGQGFWRNDCADPGNYPNDCDDGDRFIGGESDLNTGHPQIYGMFRDELANLRSGYGAGGFRFDFVRGYAPERVDSWMSDSADSSFCVGELWKGPSEYPSWDWRNTASWQQIIKDWSDRAKCPVFDFALKERMQNGSVADWKHGLNGNPDPRWREVAVTFVDNHDTGYSPGQNGGQHHWALQDGLIRQAYAYILTSPGTPVVYWSHMYDWGYGDFIRQLIQVRRTAGVRADSAISFHSGYSGLVATVSGSQQTLVVALNSDLANPGQVASGSFSEAVNASNGQVRVWRS",
                "domain2_sequence" : "VNVNFRCDNGVTQMGDSVYAVGNVSQLGNWSPASAVRLTDTSSYPTWKGSIALPDGQNVEWKCLIRNEADATLVRQWQSGGNNQVQAAAGASTSGSF"
            }
        }
    ],
    "constraints" : []
}
```

MutexaGPT:

Please confirm the mutations you want:

WT

This pattern represents the **wild-type (WT)** enzyme with no amino acid substitutions. It is used as a reference library containing the original, unmutated protein sequence without any modifications to the residue composition.

User:

Looks good.

Supplementary Section 6. Example dialog between user and MutexaGPT for setting up the non-native activity engineering study using Kimi K2.5.

User:

Help me find mutations that make my enzyme faster for an non-native substrate ethyl iodide.

MutexaGPT:

I need some more information to help you with this study:

- **Target Protein**: Could you specify which enzyme you are studying and provide its structure? (Suggestion: Please upload the PDB file for the wild-type enzyme. Also, since you mentioned a non-native substrate, you may need to provide both the apo structure and the enzyme-substrate complex with ethyl iodide.)
- **Mutations**: Could you specify what library of mutations you want to explore for improving the catalytic activity? (Suggestion: Do you want to study 'site-saturation mutations at residues within 5 angstroms of the substrate' or '50 random single-point mutations across the active site residues'?)
- **Property of Interest**: You mentioned making the enzyme "faster" for the non-native substrate ethyl iodide. Do you mean the catalytic rate or activity? (Suggestion: Do you want to study 'the catalytic activity (catalytic rate/kcat)' for the substrate ethyl iodide? Note that a proper substrate binding pose in the complex structure will be needed.)

User:

I have uploaded the wild-type atHMT in complex with the non-native substrate ethyl iodide (ETI). For mutations, I want to explore all single point mutations with in 4A of ETI. For property, it will be the catalytic efficiency of ETI.

MutexaGPT:

Here is the final decomposed question based on your input:

- **Target Protein**: the uploaded wild-type atHMT in complex with ethyl iodide (ETI) at athmt-eti.pdb
- **Mutations**: all single-point mutations within 4 angstroms of the substrate ETI
- **Property of Interest**: the catalytic efficiency for the substrate ETI

Does this accurately represent what you want to study? If everything looks correct, the workflow can proceed with this specification.

MutexaGPT:

Target property: catalytic efficiency for the substrate ETI

Catalytic efficiency (relating to both kcat and Km) can be decomposed into: **Activation Barrier** (relates to the chemical step/kcat), **Substrate Binding** (relates to substrate affinity/Km), and **Thermostability** (since mutations are involved and may affect protein stability and thus activity).

Here are the relevant computational metrics we currently support:

1. Electric Field Strength along the Bond (modeled as the "pre-reaction complex" - protein-ligand complex with a constraint applied to the "forming bond") & MMPB/GBSA Ligand Binding Energy (modeled as the protein-ligand complex without constraint) & Relative Folding Free Energy
2. Substrate Positioning Index (SPI) (modeled as the "pre-reaction complex" - protein-ligand complex with a constraint applied to the "forming bond") & MMPB/GBSA Ligand Binding Energy (modeled as the protein-ligand complex without constraint) & Relative Folding Free Energy
3. Active Site RMSD (modeled as the "pre-reaction complex" - protein-ligand complex with a constraint applied to the "forming bond") & MMPB/GBSA Ligand Binding Energy (modeled as the protein-ligand complex without constraint) & Relative Folding Free Energy

Please confirm which one you want to use.

Suggestion: #1 *Reason*: The Electric Field Strength along the Bond measured in the pre-reaction complex is specifically established to predict enzyme activity. Combined with Relative Folding Free Energy (to account for thermostability changes from mutations), this combination comprehensively covers all aspects of catalytic efficiency.

User:

Electric Field Strength & Relative Folding Free Energy

MutexaGPT:

I found the following missing information for these metrics:

1. **Electric Field Strength along the Bond** (modeled as the "pre-reaction complex" - protein-ligand complex with a constraint applied to the "forming bond")
   - **atom_1, atom_2**: The two atoms of the bond where you want to measure the electric field strength.

(Suggestion: For a methyltransferase with ethyl iodide substrate, this is typically the bond that breaks during the methyl transfer. Please specify the atoms in format "Chain_id.Residue_index.Atom_name", e.g., "A.125.SG" for the nucleophile and "A.201.C1" for a carbon on the substrate ETI)

2. **Distance Constraint** (required for the pre-reaction complex MD model)
   - **constraint atoms**: The two atoms of the "forming bond" to apply the constraint.

(Suggestion: These would typically be the atoms involved in the bond formation/elongation during catalysis. Please specify in format "Chain_id.Residue_index.Atom_name")

These information defines the question you want to study. Please be careful and let me know your choice. You can directly identify them following the EnzyHTP syntax or describe them using natural language.

User:

For EF, atom 1 and atom 2 are C.218.C1 and C.218.I1. For constraint, they are B.217.S1, C.218.C1

MutexaGPT:

Great, here is the compiled information for the target computational metrics:


```
{
    "metrics": [
        {
            "name": "electric_field",
            "arguments": {
                "atom_1": "C.218.C1",
                "atom_2": "C.218.I1"
            }
        },
        {
            "name": "ddg_fold",
            "arguments": {}
        }
    ],
    "constraints": [
        {
            "type": "distance",
            "arguments": ["B.217.S1", "C.218.C1"]
        }
    ]
}
```


MutexaGPT:

Please confirm the mutations you want:

{a:1[resn ETI around 4:all not self]}

This pattern generates a combinatorial library comprising the wild-type protein and single-point mutants. Specifically, each residue located within 4 angstroms of any residue named ETI is individually mutated to all other 19 standard amino acids, producing variants that contain either no mutations or exactly one substitution at these surrounding positions.

User:

looks good

Supplementary Section 7. The design of each individual LLM-agent.

All LLM agents in the multi-agent system of MutexaGPT conform to a similar design pattern (Supplementary Figure 1). Again, an LLM agent is a LLM model (e.g.: GPT-4o[1], etc.) configured with system prompt that instruct and regulate its behavior. Supplementary Figure 1 summarizes the system prompt in three high-level components: memory, reasoning, and tools.[2,3] The memory module stores and recalls information that informs decisions and actions of the agent.[3] In MutexaGPT, we found that the sensory memory embedded in models such as GPT-4o already contains enough domain knowledge of computational enzymology for most of our tasks. In this case, we only supplied necessary additional domain knowledge and the EnzyHTP

document in the form of short-term memory[2-4] (i.e., "Reference" section in the system prompt). These memories help agents achieve the tasks that sensory memory by itself cannot handle.

The reasoning module leverages the information in the memory and create new information by drawing conclusions or making decisions.[5, 6] In MutexaGPT, we employed strategies such as chain-of-through (CoT)[7-9] and in-context-learning (ICL)[10]. CoT means the system prompt contains a list of instructions directing the agent to follow a logic chain (explicitly), or a few sentences that simply ask the agent to use think step by step (implicitly). ICL means examples, as input and output pairs, are provided in the system prompt, also known as few-shot learning. Studies show that both of these strategies improve the performance of LLM agents.[7-10] Later in this section we will also evaluate the importance of these strategies to the final performance of the agent.

The tools are external functions, such as API calls, that an agent knows and can invoke to act. We design and implement tools in our backend and allow agents to interact with the environment, which in our case, consists of the user, UX in the frontend, and the file system in the backend. For example, when the QuestionAnalyzer wants the user to upload a PDB file of the enzyme, it can call a function tool defined via the OpenAI Assistant API and display an upload box at our frontend. Aside from the modules, we use structured data (e.g.: JSON) as much as possible as the input and output of the agents for enhanced accuracy in formatting.[11] Overall, the agents in MutexaGPT follow a unified Memory–Reasoning–Tools blueprint, and work together to convert user's initial questions or requests into MD-based molecular-level insights.

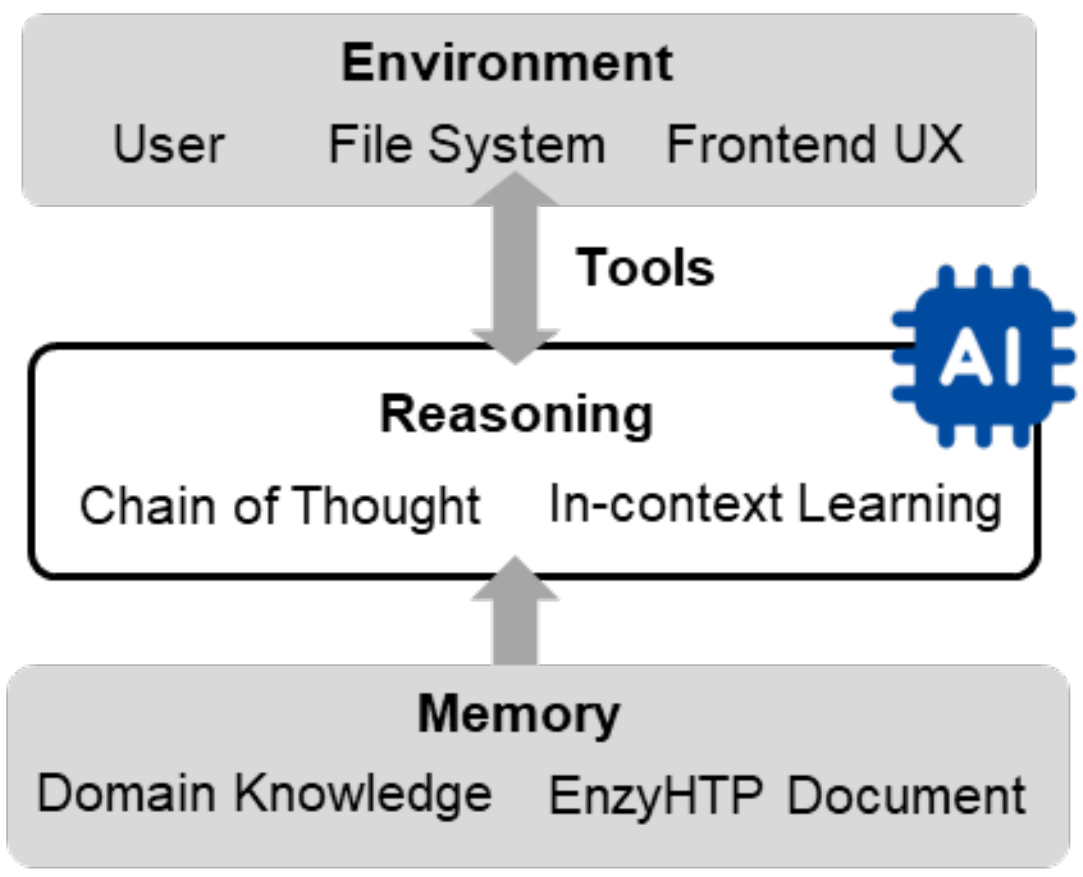

Supplementary Figure 1. The design logics of LLM agents in MutexaGPT. The agent architecture comprises three high-level components: memory, reasoning, and tools. The memory module stores and retrieves information that informs the agent's decisions and actions. The reasoning module uses information from memory to derive new information, draw conclusions, and make decisions. Tools are external functions, such as API calls, that the agent can invoke to perform actions. We design and implement these tools in the backend, enabling agents to interact with the environment, which in MutexaGPT includes the user, the frontend user interface, and the backend file system.

Supplementary Section 8. The evaluation system of LLM-agents.

To assess how effectively each agent fulfils its role and iterates their design, we designed an automated evaluation framework for MutexaGPT (Supplementary Figure 2). To test an LLM agent like QuestionAnalyzer, which takes natural language (i.e.: the scientific inquiry from user) both as input and in the form of output (i.e.: the feedback in the conversation with the user), two challenges arise: 1) how to create a test set that is diverse enough to cover the vast space natural language can span? 2) how to judge whether the natural language output of the agent is a desired one given the initial question? We addressed both challenges with purpose-built auxiliary LLM agents. The **Test Set Curator** agent converts high-level attributes of the desired test case (e.g.: "irrelevant") into a test case input that conforms the testee agent's API. It also simultaneously labels the test case by its properties, which will later be used as the ground truth against the testee agent's response. For example, in the evaluation system for QuestionAnalyzer, with the input "irrelevant", the QATestsetCurator generates a fake user inquiry that is not relevant to enzyme engineering or molecular dynamics and label this test case as "irrelevant". The curator agent is effective because the curator performs the task of synthesizing questions from known attributes. Compared with QuestionAnalyzer, this task is relatively simpler. In addition, the use of curator agents is also finite, and we manually went through all the generated test cases to ensure accuracy. For the second challenge, we created a list of **Classifier** agents that classify the natural language response of the testee agent into desired properties. For example, for QuestionAnalyzer, the agent should be able to distinguish relevant user inquiries from irrelevant ones and check users for missing details. These needs lead to two basic evaluation metrics of importance to us: "relevance" and "completeness". Accordingly, we created two specialist classifier agents: QARelevanceJudge and QACompletenessJudge. QARelevanceJudge inspects the QuestionAnalyzer's reply and decides whether it correctly classifies the user inquiry as in-scope for MutexaGPT or flags it as irrelevant. The QACompletenessJudge examines the same reply and checks which of the three required elements (enzyme target, mutation plan, engineering objective) QuestionAnalyzer thinks is still missing or underspecified. Both classifier agents give a list of labels describing the properties of care. These labels are then compared with the ground truth from the creation stage of the test cases and determine whether a test is passed or not.

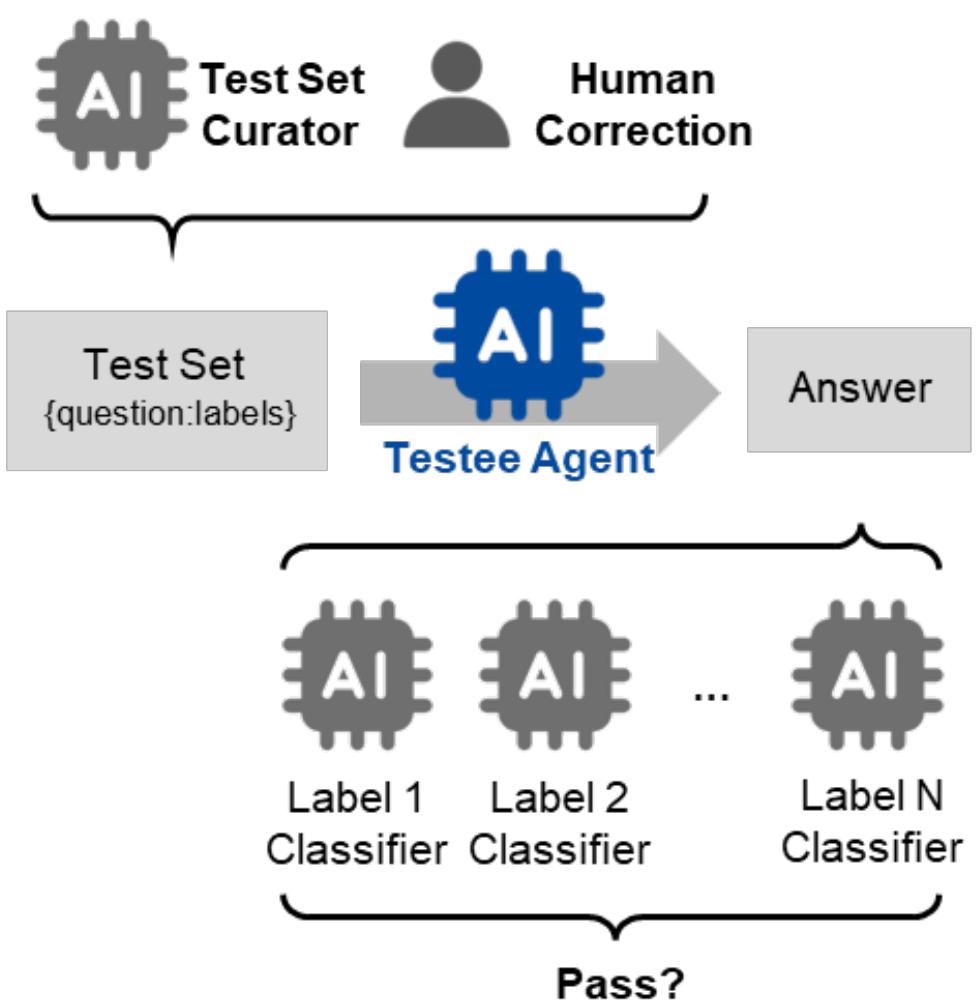

Supplementary Figure 2. The evaluation system of LLM-agents. A Test Set Curator generates test inputs from predefined test case attributes and assigns ground-truth labels. The testee agent processes each input, and specialist Classifier agents convert its natural-language response into structured labels. These labels are compared with the ground truth to determine whether each test case passes.

The detailed evaluation design of all other agents besides QuestionAnalyzer mentioned above are summarized below:

MetricsPlanner. To evaluate MetricsPlanner, we use a similar curator–extractor–judge pipeline. First, the MetPTestsetCurator generates three-line QuestionAnalyzer-style output ("Target Protein", "Mutations", "Property of Interest"), as the test inputs for MetricsPlanner, from gold labels of metric combinations and MD model context, while a separate MetPUnsupportedTestSetCurator generates clearly unsupported-property cases. MetricsPlanner then take these inputs and yield outputs. For supported cases, outputs are parsed by MetPAccuracyExtractor into normalized candidate metric sets, and a case is counted as pass only when the labeled metric(s) (and MD model when applicable) are present in the extracted candidates. For unsupported cases, MetPUnsupportedNoticeJudge verifies that the agent clearly states "unsupported" and does not continue recommendation/workflow steps. Same as the QA benchmarking, each case is run in three independent replicas.

MutantPlanner. MutantPlanner is evaluated with category-controlled natural-language requests curated by MutPTestSetCurator, which enforces five mutation-planning regimes (Unrestricted, Pocket, Interface, Distal, and Designated) and pairs each input with a reference EnzyHTP mutation pattern. MutantPlanner is required to convert the natural language input into a strict JSON output containing a single EnzyHTP pattern under "output". Pass/fail is determined by semantic equivalence to the gold pattern rather than string identity alone: if exact text does not match, both predicted and reference patterns are expanded into mutant sets by EnzyHTP (with random patterns normalized to combinatorial form for comparison), and the case passes when the two mutant sets are identical. Each case is also tested in three independent runs.

ResultExplainer. For ResultExplainer, test inputs are curated by RETestsetCurator as schema-valid JSON payloads containing scientific_question, metrics, results, downloadables, and metadata, with controlled diversity across basic, intent-edge, metric-edge, and extreme-value scenarios. Outputs are evaluated by two specialist judges. RETaskAlignmentJudge scores four binary criteria: (1) whether the explanation directly addresses the stated scientific_question; (2) whether it captures the main decision objective (or explicitly states ranking is not possible, with reason); (3) whether it focuses on metrics relevant to that objective; and (4) whether it provides a decision-oriented takeaway (prioritization or actionable recommendation). It also flags critical_misalignment for substantially off-target responses. REInterpretationBoundaryJudge scores four binary criteria: (1) whether metrics are presented as descriptors/proxies rather than direct proof; (2) whether cautious language is used (e.g., "may indicate," "suggests"); (3) whether the text explicitly states that key metrics do not directly prove activity/affinity/causality; and (4) whether causal overclaims are avoided. It separately flags overclaim_detected when major overclaim appears. A case is counted as overall pass only

when both judges pass (alignment pass and interpretation-boundary pass). Same as other agents, each test case is executed in three independent replicas.

Supplementary Section 9. Failure mode analysis in the agent performance evaluation

QuestionAnalyzer. See main text, Section 2.2.
MetricsPlanner. We summarized representative failure cases below: (1) In a case labeled as active_site_rmsd under pre-reaction complex for an activation-barrier query, the agent returned only electric_field + spi candidates and missed the labeled metric-model pair, so the case failed strict label matching. (2) In an enzyme-activity case labeled as mmpbgbsa under pre-reaction complex, the output included mmpbgbsa but under protein-ligand complex rather than the labeled MD model, which was counted as a fail. (3) In another enzyme-activity case labeled as spi under protein-ligand complex, the output contained spi only under pre-reaction complex; this MD-model mismatch also failed the strict criterion. These examples suggest the main failure pattern in the MetricsPlanner test is the "incomplete metrics option coverage" rather than completely incorrect planning. For complex properties such as enzyme activity, multiple modeling choices can be scientifically defensible (for example, different metric combinations and MD-model contexts). In several failed cases, the agent still returned plausible options, but did not include all candidate metrics (omitted the labeled metric – model pairing) required by our strict benchmark rule. Therefore, these failures mainly reflect reduced completeness of options presented to users, rather than a total loss of property-to-metric mapping capability.

MutantPlanner. We summarized representative failure cases: (1) For an interface request ("Enumerate all combinatorial single-point mutations at the dimer interface to smaller residues"), the agent's output was "a:1[byres chain A around 5.0 and chain B:smaller]" which added the single-point mutation restriction. (i.e., the labeled reference pattern is "a:[byres chain A around 5.0 and chain B:smaller]") (2) For a distal mutation case ("Mutate S141 to alanine and additionally generate 4 distal single-point mutations (>30 A from LIG) toward less negative charge."), the model messed up the pattern of the designated mutation and output "{SS141ALA, r:1[byres all and not (byres resn LIG around 30 or resn LIG):charge+]*4}", where the reference is "{S141A, r:1[byres all and not (byres resn LIG around 30 or resn LIG):charge+]*4}". Note that in both cases, the error didn't dominate all three independent runs. In fact, only one out of three have the error. Overall, errors are rare and can be detected by the EnzyHTP engine in the backend. (i.e., the parsing will fail or there will be significantly more mutants generated.)

ResultExplainer. We summarized a representative failure case: the input is

> "{"scientific_question": "How does maximum folding energy affect binding at extreme electric fields?", "metrics": "[{'name': 'ddg_fold', 'unit': 'kcal/mol', 'description': 'Folding Free Energy Change'}, {'name': 'electric_field', 'unit': 'GV/m', 'description': 'Electric Field Strength along Bond'}]", "results": "[{'wt_path': 'proteinI.pdb', 'mutant': 'WT', 'metric': 'ddg_fold', 'value': -8.0}, {'wt_path': 'proteinI.pdb', 'mutant': 'G10V', 'metric': 'ddg_fold', 'value': 8.0}, {'wt_path': 'proteinI.pdb', 'mutant': 'WT', 'metric': 'electric_field', 'value': 5.0}, {'wt_path': 'proteinI.pdb', 'mutant': 'G10V', 'metric': 'electric_field', 'value': -5.0}]",

"downloadables": "[{'file_type': 'csv', 'description': 'full results table', 'filename': 'extreme_values_0009_full.csv'}, {'file_type': 'pickle', 'description': 'trajectory data', 'filename': 'extreme_values_0009_traj.pkl'}]", "metadata": {"simulation_engine": "AMBER", "temperature_K": 320.0, "production_ns": 150, "date": "2023-10-23T17:45:00Z"}}".
The agent's output passed the interpretation-boundary judge test but failed the task-alignment test due to not providing a decision orientated takeaway as it mentions "this dataset does not directly measure binding, so it can only suggest whether folding-energy change and electric-field values move together across variants.". This is likely an ill-defined test case as the "results" in the payload is not enough to answer the "scientific question" and ResultExplainer is doing a good job explaining this incapability. We have check other cases in the test set and found no similar problem.

Supplementary Section 10. Example ResultExplainer output and Summary page screenshot for each use case.

Use case 1: Cavity Engineering

Executive Summary

Your main question was which acl-MT mutations most strongly **increase active-site cavity volume** relative to the uploaded wild type. Based on the reported **Cavity (Å^3)** values, many variants exceed WT (**524.0 Å^3**), and the biggest cavity-expanding candidates are concentrated in combinations involving **LA39H**, **VA11F**, **LA30I/M**, **WA31Y**, and in some cases **WA41F**. Top performers include **LA39H VA11F (2807.5 Å^3)**, **LA30I VA11A WA31Y (2717.7 Å^3)**, **LA39H LA30M VA11L (2641.1 Å^3)**, and **LA39D LA30F WA31Y VA11I (2640.1 Å^3)**. These are strong candidates if your goal is to **maximize geometric cavity size**.

Simulation context is relatively strong for screening: **600 variants**, **50 ns production MD**, **1 ns equilibration**, **Amber**, **ff14SB+GAFF**, **TIP3P**, with equilibration assessed by the **Chodera automated equilibration detection scheme**; **600/600** replica assessments were marked equilibrated. Confidence for prioritizing variants by cavity size: **Good**. That is good enough to shrink a library, but still not enough to prove improved substrate scope, catalysis, or stability, especially since there is only **1 replica per variant** and only one metric is provided.

- raw_results.csv — table of all reported variant results.
- plots/equilibration — equilibration outputs, including equil_assessment.json, energy, pressure, and temperature plots.

Ranked Candidates

Ranking logic: because your scientific question is specifically about **increasing cavity size**, I ranked mutants by **Cavity (Å^3)** from highest to lowest. No stability-first filter was applied because no stability-related metric was provided.

1. **LA39H VA11F — 2807.5 Å^3**; about **5.36× WT**.
2. **LA30I VA11A WA31Y — 2717.7 Å^3**; about **5.19× WT**.
3. **LA39H LA30M VA11L — 2641.1 Å^3**; about **5.04× WT**.
4. **LA39D LA30F WA31Y VA11I — 2640.1 Å^3**; about **5.04× WT**.

5. **WA31Y LA39H VA11F — 2530.8 Å^3**; about **4.83× WT**.
6. **LA39H WA31Y — 2511.3 Å^3**; about **4.79× WT**.
7. **LA30I WA41F VA11I — 2502.5 Å^3**; about **4.78× WT**.
8. **WA41F LA39D VA11I LA30M WA31Y — 2470.4 Å^3**; about **4.71× WT**.
9. **LA30I LA39H VA11F WA31Y — 2357.7 Å^3**; about **4.50× WT**.
10. **WA31Y LA39H LA30F VA11F — 2351.5 Å^3**; about **4.49× WT**.

Notable simpler mutants if you want easier experimental entry points:

- **LA39H VA11F** — strongest 2-mutation hit.
- **LA39H WA31Y** — very large 2-mutation hit.
- **LA30I LA39I — 2226.1 Å^3**.
- **LA39D VA11A — 2248.6 Å^3**.
- **WA41F WA31Y — 2042.5 Å^3**.

Metric-by-Metric Insights

Cavity (Å^3)

- **What it may indicate:**
  This is a geometric descriptor of how much empty pocket space is present in the active site region. In your project, a larger value may indicate that a mutation opens the pocket or creates more room for substrate binding or altered substrate size preference.
- **What it does NOT directly prove:**
  It does **not** directly prove stronger binding, better catalysis, broader substrate scope, higher methyltransferase activity, or better protein stability. A larger cavity can also come from local opening, rearrangement, or a less well-packed active site that is not beneficial.
- **WT/reference pattern:**
  WT is **524.0 Å^3**. Many mutants are substantially larger than WT, while some are smaller than WT, showing that the library explores both opening and constricting effects.
- **Mutants that stand out:**
  Largest increases: **LA39H VA11F**, **LA30I VA11A WA31Y**, **LA39H LA30M VA11L**, **LA39D LA30F WA31Y VA11I**, **WA31Y LA39H VA11F**, **LA39H WA31Y**.
  Strong single mutants relative to WT include **WA41F (1393.0 Å^3)**, **VA11F (1492.5 Å^3)**, **LA39D (1367.4 Å^3)**, **LA39I (1290.7 Å^3)**, and **LA30I (1547.6 Å^3)**.
  Some mutations can also sharply reduce cavity size depending on context, for example **WA41F LA39H VA11A (214.3 Å^3)** and **LA30I LA39T VA11F (236.8 Å^3)**.
- **Confidence / caveat:**
  Good for prioritizing variants by predicted cavity expansion because the MD metadata are fairly complete and equilibration was assessed for all runs. Main caveats are **single replica per variant** and lack of complementary metrics, so these values should be treated as screening-level structural proxies.

Recommended Follow-Ups

Recommended Follow-Ups

1. **Experimentally test the top cavity-expanded variants** first, especially a mix of high-ranking simple and complex mutants such as **LA39H VA11F**, **LA39H**

WA31Y, **LA30I LA39I**, **LA30I VA11A WA31Y**, and **LA39D LA30F WA31Y VA11I**.

2. **Inspect trajectories and pocket shapes visually** for the top hits. A large average cavity can come from a useful open conformation, but it can also come from irregular opening or partial local unfolding.
3. **Run longer and replicated simulations** for the best candidates. Extra replicas would help determine whether the largest cavity values are robust or driven by one sampled trajectory, and adding stability or active-site contact metrics would tie the results more closely to function.

Appendix — Definitions

- **Molecular dynamics (MD):** a computer simulation that models how atoms in a protein move over time.
- **Equilibration:** the early part of a simulation where the system settles before measurements are interpreted.
- **Cavity volume:** a **geometric descriptor** of pocket size, not a direct measurement of activity or binding.
- **Replica:** an independent repeat simulation used to test whether a result is reproducible.
- **Force field:** the mathematical model used to approximate atomic interactions in the simulation.
- **Solvent model:** the representation used for water and surrounding liquid in the simulation.

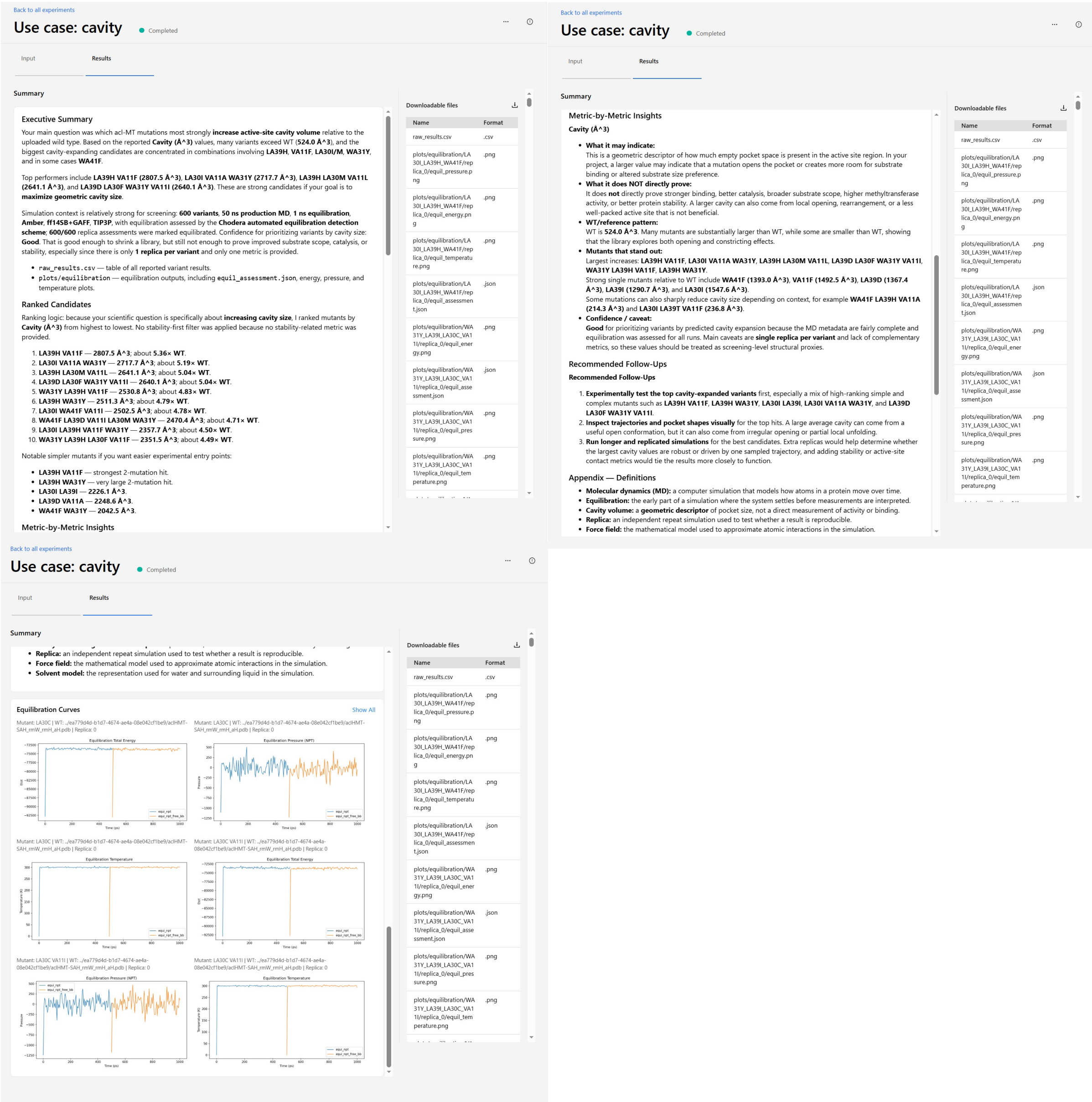

Supplementary Figure 3. Screenshots of the Summary page for use case 1: Cavity Engineering. The summary page contains a summary generated by the ResultExplainer agent as final response to the original research question from the user, a list of downloadable source files, and a panel of quality control figures showing the convergence of the simulation.

Use case 2: Cold Adaptation Engineering

Executive Summary

The main usable signal here is the **Domain Separation Index, DSI (scalar)**, which your input describes as being predictive of cold adaptation in bidomain enzymes. Under that assumption, **higher DSI is the favorable direction** for your question about maintaining activity at 0°C relative to 45°C. Based on this metric alone, the most promising linker designs are **psAt6** and **psAt5**, with **psAt6** standing out strongly above all others and above the original WT structure.

Simulation context is reasonably documented: **11 variants**, **50 ns production MD** each, **1 ns equilibration**, **1 replica per variant**, run in **Amber** with **ff14SB+GAFF** and **TIP3P** water. Equilibration was assessed using the **Chodera automated equilibration detection scheme**, and **11/11 replicas were marked equilibrated**. Overall confidence is **Fair**: the setup is described and equilibration looks acceptable, but there is only **one replica per variant**, one temperature (**300 K**) rather than cold/heat conditions directly, and only one model-derived metric tied indirectly to cold adaptation.

- Downloadable: **raw_results.csv** — raw per-variant metric values.

Ranked Candidates

Ranking logic: I ranked designs by **DSI (scalar)** from highest to lowest, because your metric description states that DSI is predictive of cold-adaptation behavior in bidomain enzymes. Since no direct activity measurements or explicit stability metrics were provided, this is a **proxy-based prioritization**, not proof of improved cold activity.

1. **psAt6** — DSI **20.61**; much higher than WT.pdb (**5.54**), strongest predicted shift toward favorable inter-domain dynamics for cold adaptation.
2. **psAt5** — DSI **9.48**; clearly above WT.pdb, second-best candidate.
3. **WT.pdb** — DSI **5.54**; the original WT reference is mid-to-high in this set.
4. **psAt3** — DSI **4.66**; somewhat below WT.pdb but still among the higher-scoring linker designs.
5. **psAt10** — DSI **3.42**; positive but lower than WT.pdb.
6. **psAt9** — DSI **1.23**; modest signal.
7. **psAt8** — DSI **0.61**; weak positive signal.
8. **psAt2** — DSI **-0.076**; near zero, not encouraging.
9. **psAt1** — DSI **-0.305**; slightly unfavorable relative to WT.pdb.
10. **psAt7** — DSI **-0.364**; slightly more unfavorable.

Lowest-ranked overall:

- **psAt4** — DSI **-1.36**; least favorable by this metric.

Metric-by-Metric Insights

Domain Separation Index (scalar)

- **What it may indicate:** A larger DSI may indicate that the two enzyme domains move more independently or remain more separated in ways associated, in prior work, with better cold-adaptive behavior in bidomain enzymes.
- **What it does NOT directly prove:** It does not directly measure catalytic rate, substrate binding, turnover at 0°C, or the actual ratio of activity at 0°C versus 45°C. It also does not by itself prove that a linker variant is stable, well-folded, or experimentally expressible.
- **WT/reference pattern:** The original **WT.pdb** value is **5.54**. Two linker designs exceed it substantially: **psAt6 (20.61)** and **psAt5 (9.48)**. Several designs fall below WT, and some are negative.
- **Mutants that stand out:** **psAt6** is the clearest positive outlier. **psAt5** is the next strongest. At the unfavorable end, **psAt4** is the clearest negative outlier.
- **Confidence / caveat: Fair**. The metric is relevant to the scientific question because your JSON explicitly links DSI to cold adaptation, but confidence is limited by the lack

of replicate simulations, the single-metric basis for ranking, and the absence of direct low-temperature functional measurements.

Recommended Follow-Ups

Recommended Follow-Ups

1. **Experimentally test psAt6 and psAt5 first**, and likely include **psAt3** as a secondary candidate, by measuring the actual **relative catalytic activity at 0°C vs 45°C**. This is the key validation because DSI is only a predictor.
2. **Run longer and replicated MD simulations** for the top candidates and WT. Multiple replicas would show whether the very high DSI for psAt6 is robust or just a single-trajectory effect.
3. **Inspect trajectories and add complementary analyses** tied to linker behavior, such as inter-domain distance distributions, linker flexibility, and active-site geometry. These could help explain *why* a linker raises DSI and whether that change is likely to support catalysis rather than just increase motion.

Appendix — Definitions

- **Molecular dynamics (MD):** A computer simulation method that models how atoms move over time.
- **Domain Separation Index (DSI):** A model-derived descriptor of how separately two protein domains move; here it is a **proxy** for cold adaptation, not a direct activity measurement.
- **Equilibration:** The early part of a simulation where the system settles before analysis; it is not itself proof that the simulation fully sampled all relevant conformations.
- **Replica:** An independent repeat simulation used to test whether a result is reproducible.
- **Force field:** The mathematical model used to estimate atomic interactions in the simulation.
- **Solvent model:** The representation of water in the simulation; here it is part of the computational setup, not an experimental measurement.

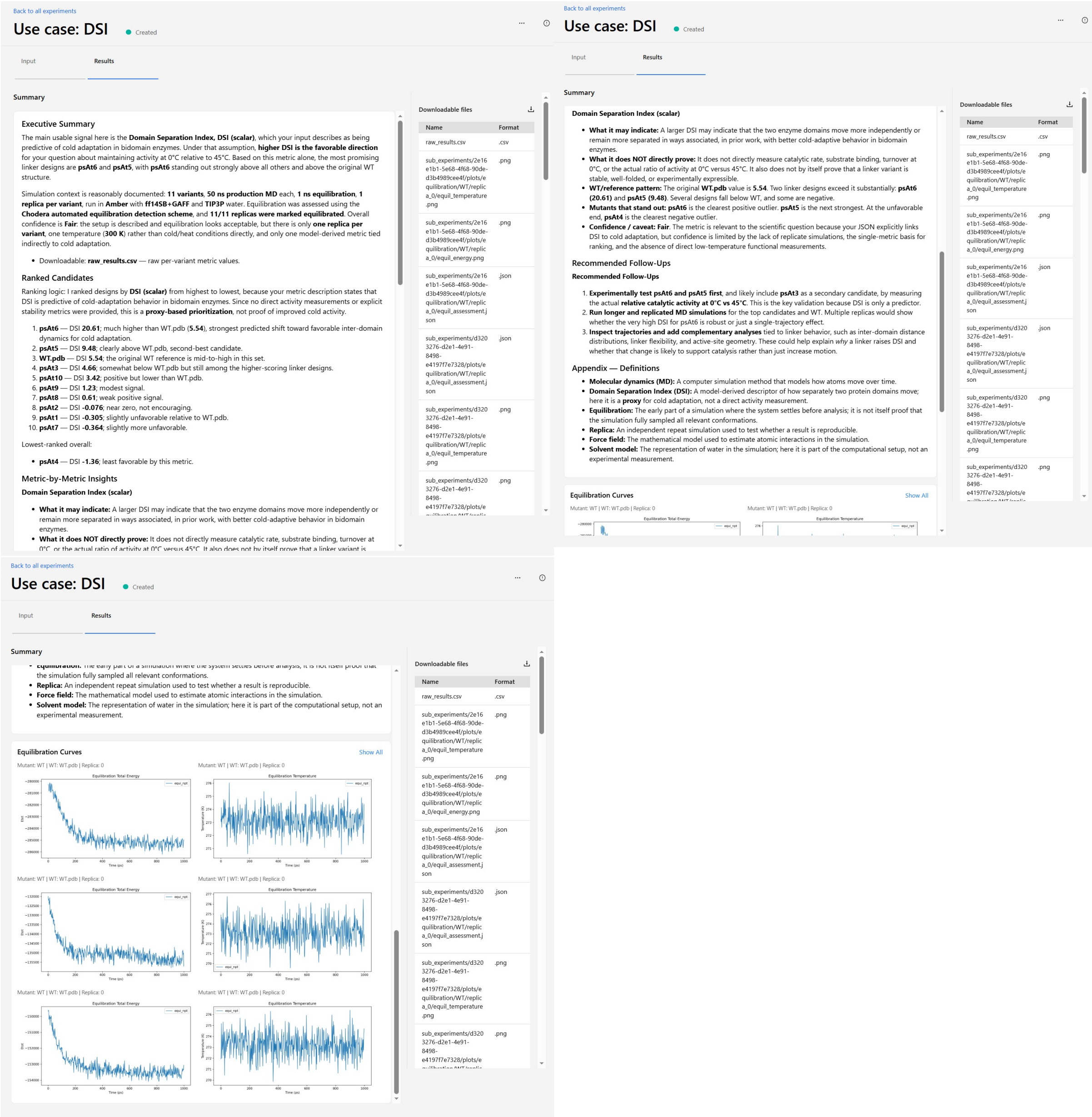

Supplementary Figure 4. Screenshots of the Summary page for use case 2: Cold Adaptation Engineering. The summary page contains a summary generated by the ResultExplainer agent as final response to the original research question from the user, a list of downloadable source files, and a panel of quality control figures showing the convergence of the simulation.

Use case 3: Non-native Enzyme Activity Engineeering

Executive Summary

This dataset helps narrow which single-point mutants near ETI are most worth testing for improved **catalytic efficiency (kcat/KM)**, but it does **not** directly measure kcat/KM. The most decision-useful strategy here is to first remove mutants predicted to disrupt folding, then prioritize mutants with the most favorable **Electric field (MV/cm)** if we assume that a **more negative field along the target bond** is beneficial for ETI chemistry. Under that assumption,

the strongest candidates are mainly at **A132**, with **A132E, A132T, A132I, A132H, and A132M** standing out.

Simulation context is fairly strong for screening: **191 variants**, **50 ns production** each, **1 ns equilibration**, **1 replica per variant**, run in **Amber** with **ff14SB+GAFF** and **TIP3P** solvent at **300 K**. Equilibration was assessed by the **Chodera automated equilibration detection scheme**, and **191/191** replica assessments were marked equilibrated. Confidence is **Good** for triaging variants, but not Very Good, because the dataset uses only **one replica per variant** and relies on proxy metrics rather than direct catalytic calculations or experiments.

- raw_results.csv — table of all mutant metric values.
- plots/equilibration/equil_assessment.json and the energy/pressure/temperature plots — equilibration diagnostics across variants.

Ranked Candidates

Ranking logic: I applied a **stability-first filter** using **ddG fold (Rosetta Energy Unit)**, keeping the most favorable ~33% of mutants (lowest ddG values), then ranked those by **Electric field (MV/cm)**. This assumes that a more negative electric field along the target bond is more favorable for the desired ETI reaction.

1. **A132E** — Electric field **-12.87 MV/cm**; ddG fold **3.95 REU**. Much more negative field than WT (**-6.92 MV/cm**), but predicted less stable than WT.
2. **A132T** — Electric field **-11.69 MV/cm**; ddG fold **3.16 REU**. Stronger field than WT, modest predicted stability penalty.
3. **V128C** — Electric field **-11.59 MV/cm**; ddG fold **3.44 REU**. Stronger field than WT, but some folding penalty.
4. **V11F** — Electric field **-11.20 MV/cm**; ddG fold **4.91 REU**. Stronger field, but stability concern is larger.
5. **P170F** — Electric field **-10.95 MV/cm**; ddG fold **-0.95 REU**. Good combination of favorable field and no predicted folding penalty.
6. **A132I** — Electric field **-10.86 MV/cm**; ddG fold **-0.95 REU**. Strong field with favorable predicted stability.
7. **Y160C** — Electric field **-10.52 MV/cm**; ddG fold **5.30 REU**. Good field, but larger stability concern.
8. **V11T** — Electric field **-10.45 MV/cm**; ddG fold **5.10 REU**. Similar tradeoff: field gain, stability risk.
9. **V128F** — Electric field **-10.43 MV/cm**; ddG fold **-1.66 REU**. Attractive balanced candidate.
10. **V128T** — Electric field **-10.21 MV/cm**; ddG fold **4.43 REU**. Better field than WT, moderate stability cost.

If you want the most balanced experimental starting set, **P170F, A132I, V128F, A132H, and A132M** are especially attractive because they combine favorable or neutral predicted folding effects with improved electric field.

Metric-by-Metric Insights

Electric field (MV/cm)

- What it may indicate: This is a model-derived descriptor of how the enzyme environment biases charge distribution along a chosen substrate bond. For this question,

it may reflect whether a mutant better preorganizes the active site for ETI bond polarization and potentially faster chemistry.

- What it does NOT directly prove: It does **not** directly measure **kcat**, **KM**, or **kcat/KM**. It also does not prove that ETI binds correctly, stays in a productive pose, or reaches the transition state more easily.
- WT/reference pattern: WT has **-6.92 MV/cm**. Several mutants are substantially more negative than WT, especially **A132E (-12.87)**, **A132T (-11.69)**, **V128C (-11.59)**, **V11F (-11.20)**, **P170F (-10.95)**, and **A132I (-10.86)**.
- Mutants that stand out: The strongest field shifts cluster at **A132**. Among those, **A132I, A132H, and A132M** are notable because they also avoid large folding penalties.
- Confidence / caveat: **Fair to Good.** The simulation context is decent, but the crucial limitation is conceptual: electric field is only a **proxy** for catalytic efficiency, and the favorable direction is inferred rather than explicitly validated in the JSON.

ddG fold (Rosetta Energy Unit)

- What it may indicate: This is a model-derived estimate of how a mutation changes protein folding stability relative to WT. Lower values, especially near or below zero, usually suggest less risk of destabilizing the fold.
- What it does NOT directly prove: It does **not** directly measure expression, solubility, thermal stability, active-site integrity during catalysis, or enzymatic activity.
- WT/reference pattern: WT is **0.0 REU**. Many mutations outside a few tolerated positions show strongly positive values, suggesting likely instability risk. Some mutants are predicted more favorable than WT, including **A132F (-6.99)**, **A132L (-5.31)**, **A132Y (-5.24)**, **A132W (-5.00)**, **V128W (-4.74)**, **Y160W (-4.30)**, **A132M (-4.04)**, **V11I (-1.67)**, **V128F (-1.66)**, and **P170F (-0.95)**.
- Mutants that stand out: **A132** is unusually permissive, with many substitutions showing favorable ddG values. In contrast, many **C131, W24, W35, and Y160** substitutions are strongly destabilizing.
- Confidence / caveat: **Good** as a screening filter, but only for folding-risk prioritization. The values are in **Rosetta Energy Units**, so they should be treated as a comparative score, not a direct experimental free energy.

Recommended Follow-Ups

Recommended Follow-Ups

1. **Experimentally test a focused panel** for ETI **kcat, KM, and kcat/KM**, prioritizing balanced candidates such as **P170F, A132I, V128F, A132H, A132M**, plus a few high-field/high-risk mutants like **A132E** and **A132T**.
2. **Run longer and replicated simulations** for the top 5–10 candidates, because this dataset has only **1 replica per variant**. Replicates would show whether the electric-field ranking is reproducible.
3. **Inspect trajectories and substrate poses** for the top candidates to confirm ETI remains in a productive orientation; adding analyses more directly tied to catalysis or binding would strengthen the link to **kcat/KM**.

Appendix — Definitions

- **Electric field**: a **model-derived descriptor** of the local electrostatic environment along a chosen bond; it is a proxy for possible catalytic preorganization, not a direct activity measurement.
- **ddG fold**: a **model-derived stability score** estimating how a mutation may change folding stability relative to WT; it is not a direct experimental stability measurement.
- **Equilibration**: the early part of a simulation where the system settles before analysis.
- **Replica**: an independent repeat simulation of the same variant.
- **WT**: wild type, the original reference enzyme sequence.
- **Catalytic efficiency (kcat/KM)**: an experimental enzyme-performance measure describing how effectively an enzyme converts substrate at low substrate concentration.

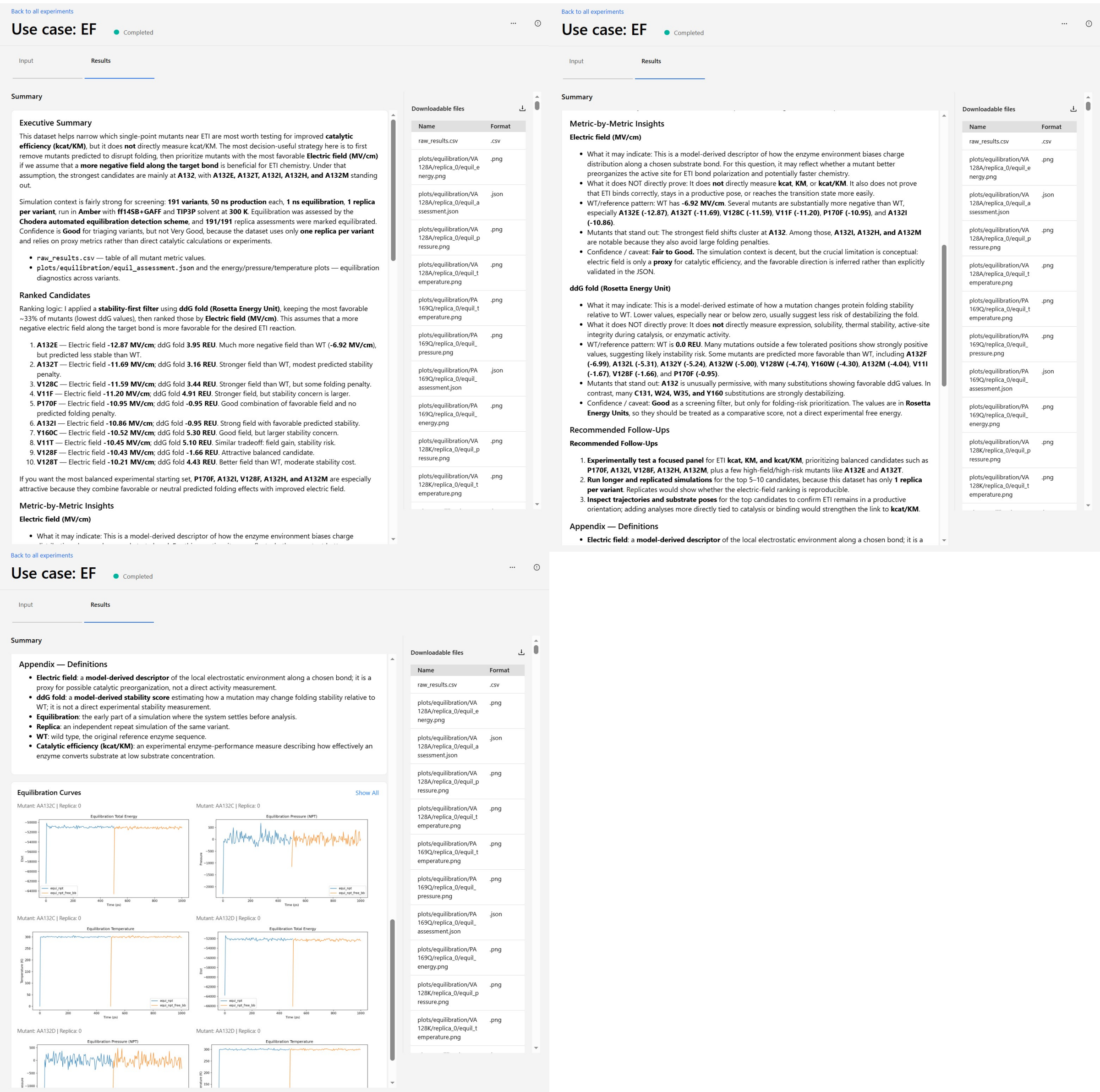

Supplementary Figure 5. Screenshots of the Summary page for use case 3: Non-native Enzyme Activity Engineering. The summary page contains a summary generated by the ResultExplainer agent as final response to the original research question from the user, a list of

downloadable source files, and a panel of quality control figures showing the convergence of the simulation.

Supplementary Section 11. Example ResultExplainer output and Summary page screenshot for each use case. (model = Kimi K2.5)

Use case 1: Cavity Engineering

Executive Summary

The analysis identified specific point mutations and combinations in the acl-MT combinatorial library that significantly expand the active site binding pocket. The top-performing variants show **4- to 5-fold increases** in cavity volume compared to wild-type, with the largest cavities reaching approximately **2,800 Å³** (WT: 524 Å³).

Main takeaway: Mutations at positions 39 (H or D), 30 (I or F), and 11 (F, I, or L) combined with the W31Y mutation produce the largest cavity expansions. Position 39 to histidine consistently appears in top performers.

Simulation reliability: Good. The dataset includes 600 variants with 50 ns production runs each. All replicas passed the Chodera automated equilibration detection. The ff14SB+GAFF force field with TIP3P solvent at 300K provides reasonable confidence for this structural screening purpose, though this is a single-replica screening suitable for narrowing candidates rather than establishing quantitative correlations.

Available files:

- aclHMT-SAH_rmW_rmH_aH.pdb — starting structure
- raw_results.csv — complete numerical results
- equil_assessment.json, equil_energy.png, equil_pressure.png, equil_temperature.png — equilibration diagnostics

---

Ranked Candidates

Sorted by **cavity (Å³)** in descending order to identify the largest active site expansions. No stability filter was applied as no stability metrics were provided in this dataset.

Rank	Mutant	Cavity (Å³)	vs. WT
1	**LA39H VA11F**	2,807.5	**+5.4×**
2	**LA30I VA11A WA31Y**	2,717.7	**+5.2×**
3	**LA39H LA30M VA11L**	2,641.1	**+5.0×**
4	**LA39D LA30F WA31Y VA11I**	2,640.1	**+5.0×**
5	**WA31Y LA39H VA11F**	2,530.8	**+4.8×**
6	**LA39H WA31Y**	2,511.3	**+4.8×**
7	**LA30I WA41F VA11I**	2,502.5	**+4.8×**
8	**WA41F LA39D VA11I LA30M WA31Y**	2,470.4	**+4.7×**
9	**LA30I LA39H VA11F WA31Y**	2,357.7	**+4.5×**
10	**WA31Y LA39H LA30F VA11F**	2,351.5	**+4.5×**

Key pattern: Position 39 mutations to H or D, combined with W31Y and specific substitutions at position 11 (F, I, L) consistently produce the largest cavities. The single mutation LA39H

alone achieves 564 Å³, but combining it with other positions (especially 11F or 31Y) yields multiplicative effects.

Metric-by-Metric Insights

cavity (Å³)

- **What it may indicate:** Larger cavity volume suggests more space available for substrate binding or accommodation of larger halide substrates in the methyltransferase active site.
- **What it does NOT directly prove:** It does not prove the enzyme will bind substrates better, have higher catalytic turnover, or that the cavity is physically accessible to substrates. It is purely a geometric descriptor of void space.
- **WT/reference pattern:** The WT structure shows 524 Å³. Many variants actually shrink the pocket (lowest: 214 Å³), while others dramatically expand it.
- **Mutants that stand out:** The top 10 mutants all exceed 2,300 Å³. Notably, LA39H (position 39 to histidine) appears in 6 of the top 10, often combined with VA11F, WA31Y, or LA30I/M.
- **Confidence / caveat:** The cavity calculation is sensitive to probe radius and protein conformation. These are single 50-ns snapshots; dynamic fluctuations could alter accessibility. Some large cavities may be "dead ends" or disconnected from the substrate entry path. The metric does not distinguish between useful expansion and irrelevant void formation.

Recommended Follow-Ups

1. **Visual inspection of top-ranked variants:** Download the PDB and trajectory files for the top 10 candidates (especially LA39H VA11F and LA30I VA11A WA31Y) to visually inspect cavity location and connectivity. Confirm the cavity is connected to solvent or substrate entry channels, not buried voids.
2. **Docking or substrate placement:** The current analysis only measures empty space. Perform substrate docking with your target halides to verify the enlarged cavities can actually accommodate the desired substrates in catalytically relevant poses.
3. **Stability validation:** The dataset lacks stability metrics (RMSD, RMSF, B-factors). Run additional simulations on the top 5 candidates with stability monitoring and longer trajectories (100+ ns) to ensure these large cavities don't compromise protein stability or induce unfolding.
4. **Experimental validation:** Test the top 3-5 candidates (particularly those with single or double mutations like LA39H VA11F or LA30I VA11A WA31Y) for actual methyltransferase activity with bulkier substrates to correlate cavity size with functional improvement.

Appendix — Definitions

- **cavity (Å³)** — A geometric descriptor calculating the volume of void space within the protein's active site region using a probe sphere; measured in cubic angstroms. It is a computational proxy for potential binding space, not a measurement of actual substrate occupancy or catalytic efficiency.

- **Equilibration** — The initial simulation phase where the system settles into a stable energy state. The Chodera scheme automatically determines when temperature, pressure, and energy fluctuations stabilize before collecting production data.
- **ff14SB+GAFF** — Combined protein force field (ff14SB for amino acids) and general Amber force field (GAFF) for any small molecules or cofactors in the system.
- **TIP3P** — A standard model for simulating water molecules in the simulation box.

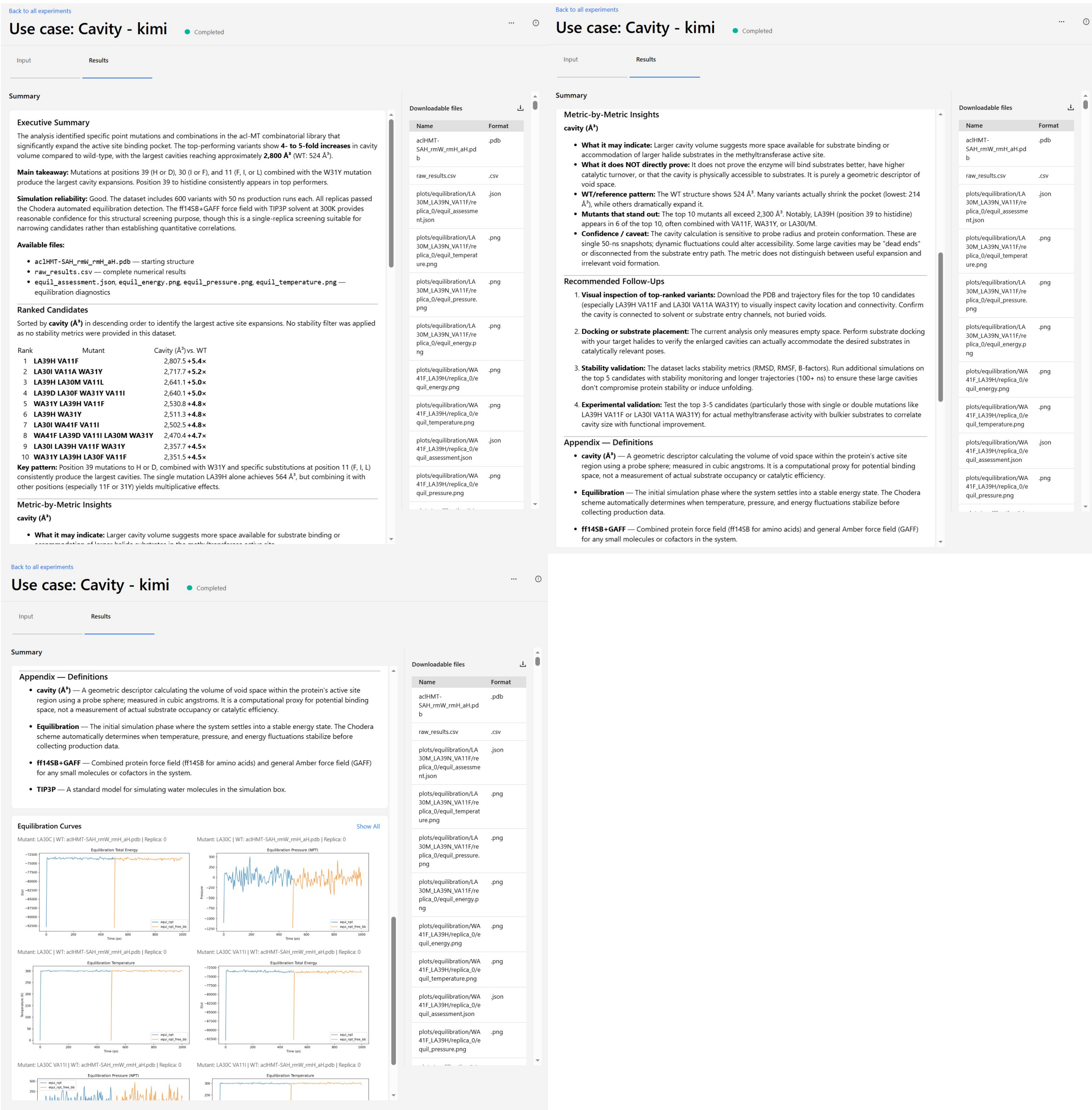

Supplementary Figure 6. Screenshots of the Summary page for use case 1: Cavity Engineering using Kimi K2.5. The summary page contains a summary generated by the ResultExplainer agent as final response to the original research question from the user, a list of downloadable source files, and a panel of quality control figures showing the convergence of the simulation.

Use case 2: Cold Adaptation Engineering

Executive Summary

The computational screening identifies **psAt6** and **psAt5** linker variants as the most promising candidates for enhanced cold-adaptation, showing Domain Separation Index (DSI) values of 20.61 and 9.48 respectively—3.7- and 1.7-fold higher than the WT baseline (5.54). According to literature, higher DSI correlates with better activity retention at cold temperatures in bidomain enzymes.

Decision: Prioritize psAt6 and psAt5 for experimental testing of 0°C activity retention.

Simulation reliability: Poor-to-Fair for quantitative predictions but adequate for preliminary triaging. The dataset comprises 11 variants (10 linkers + WT), each with 50 ns production simulations (Amber, ff14SB+GAFF/TIP3P) following 1 ns equilibration. All trajectories were confirmed equilibrated via the Chodera automated method. However, single-replica sampling with short trajectories lacks statistical power for robust convergence or correlation confidence. These results are suitable for prioritizing a short experimental candidate list, not for establishing quantitative structure-function relationships.

Available download:

- raw_results.csv — Complete DSI dataset for all variants.

Ranked Candidates

Variants are ranked by Domain Separation Index (DSI) in descending order; higher DSI indicates greater domain separation, which prior literature (Ning et al.) associates with improved cold-adaptation. No stability pre-filter was applied because stability-specific metrics were unavailable.

1. **psAt6** (psAt6.pdb) — DSI: 20.61; ~3.7× higher than WT (5.54). Exceptional outlier suggesting substantially enhanced domain separation.
2. **psAt5** (psAt5.pdb) — DSI: 9.48; ~1.7× higher than WT. Secondary strong candidate.
3. **psAt3** (psAt3.pdb) — DSI: 4.66; 0.84× WT (slightly below baseline).
4. **psAt10** (psAt10.pdb) — DSI: 3.42; 0.62× WT (substantially reduced).
5. **psAt9** (psAt9.pdb) — DSI: 1.23; 0.22× WT.
6. **psAt8** (psAt8.pdb) — DSI: 0.61; 0.11× WT.
7. **psAt2** (psAt2.pdb) — DSI: -0.08; essentially zero separation.
8. **psAt1** (psAt1.pdb) — DSI: -0.30; negative value indicating "anti-separation" or different correlated motion.
9. **psAt7** (psAt7.pdb) — DSI: -0.36; low/negative separation.
10. **psAt4** (psAt4.pdb) — DSI: -1.36; lowest score, suggesting more compact or correlated domain motion than WT.

Metric-by-Metric Insights

Domain Separation Index (DSI) (scalar)

- **What it may indicate:** Based on prior literature, DSI quantifies how independently the two enzyme domains move dynamically. Higher DSI suggests the domains flex and separate more freely, which has been correlated with the ability of bidomain enzymes to maintain catalytic activity at lower temperatures (cold-adaptation). This may relate to the linker allowing sufficient conformational freedom for catalytic site alignment when overall molecular motions slow at 0°C.

- **What it does NOT directly prove:** DSI is a **geometric/dynamic descriptor**, not a direct experimental measurement of catalytic rate (kcat/Km), binding affinity, or actual relative activity at 0°C versus 45°C. It is a computational proxy score. High DSI does not guarantee that the variant will actually perform better at cold temperatures; it only flags candidates for which the computational model predicts favorable domain dynamics based on prior statistical correlations.
- **WT/reference pattern:** The original WT.pdb reference registers DSI = 5.54. Two variants (psAt6 and psAt5) exceed this substantially. Five variants show positive but lower-than-WT values. Four variants exhibit negative DSI values (psAt1, psAt2, psAt4, psAt7), indicating domain motions that are correlated or "anti-separated" compared to the reference frame.
- **Mutants that stand out: psAt6** is a dramatic outlier (DSI = 20.61), showing roughly four-fold greater domain separation than WT—an extremely large dynamic effect given the short simulation. **psAt5** shows a robust but less extreme enhancement (9.48). These two represent the primary computational hits. Conversely, psAt4 (-1.36) and psAt7 (-0.36) show the most reduced domain separation.
- **Confidence / caveat: Poor**. The metric derives from a single literature correlation, not a validated mechanistic equation. The underlying simulations (50 ns, single replica) provide insufficient sampling to assess convergence of the domain motion statistics, meaning the DSI values could reflect transient fluctuations rather than stable conformational differences. Results are exploratory screening priorities, not reliable activity predictions.

Recommended Follow-Ups

1. **Experimental cold-activity validation:** Synthesize the top two candidates (psAt6 and psAt5) alongside WT and the low-scoring psAt4 (as a negative control). Measure their relative catalytic activity at 0°C versus 45°C to test the hypothesis that high DSI correlates with cold-adaptation. Given the computational uncertainty, these predictions require experimental confirmation before concluding anything about structure-function relationships.
2. **Extended MD sampling:** For psAt6, psAt5, and WT, run triplicate 200–500 ns production simulations or add multiple replicas (n=3–5) to assess whether the observed DSI differences are stable and reproducible. The current 50 ns single-replica data is insufficient to distinguish genuine linker-dependent dynamics from sampling noise.
3. **Mechanistic trajectory inspection:** Visually examine the psAt6 and psAt5 trajectories to determine the physical basis of the high DSI (e.g., extended linker conformations, specific hinge angles, or domain opening events). Complement this with active-site-specific metrics (e.g., catalytic cleft geometry, substrate accessibility) to bridge the domain-level description to actual catalytic function.

Appendix — Definitions

- **Domain Separation Index (DSI):** A computational descriptor quantifying the degree of independent motion between two protein domains based on MD trajectory analysis; it is a **geometric proxy** derived from simulation data, not a direct experimental measurement of domain behavior.

- **Equilibration (Chodera automated method):** A statistical algorithm detecting when a simulation has reached a thermodynamically representative state; all 11 trajectories passed this check.
- **Production simulation:** The phase of MD used for analysis after equilibration; here, 50 ns is considered short for converging protein dynamics, limiting confidence in metric stability.
- **Force field (ff14SB+GAFF):** A parameterized energy function describing atomic interactions—ff14SB for standard protein components and GAFF for general (possibly non-standard) atoms in the system.

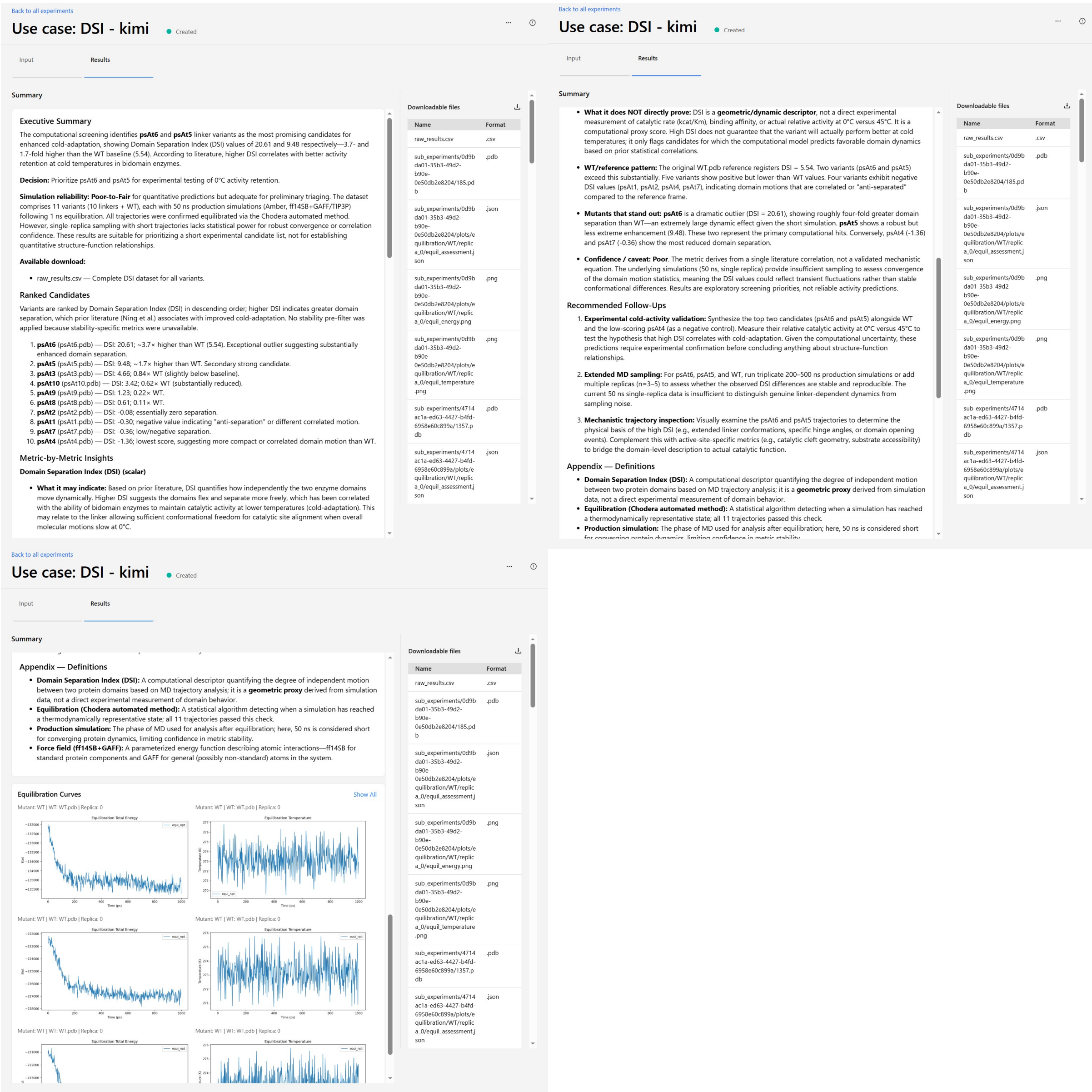

Supplementary Figure 7. Screenshots of the Summary page for use case 2: Cold Adaptation Engineering using Kimi K2.5. The summary page contains a summary generated by the ResultExplainer agent as final response to the original research question from the user, a list of

downloadable source files, and a panel of quality control figures showing the convergence of the simulation.

Use case 3: Non-native Enzyme Activity Engineeering

Executive Summary

The simulations screened 191 single-point mutations near the ETI substrate to identify variants with enhanced catalytic electrostatic environments. After filtering for stability (keeping the top 33% most stable variants), several candidates—particularly **A132E/T/I**, **V128C/F**, and **P170F**—show electric field strengths up to 86 % stronger than wild-type (-12.9 vs -6.9 MV/cm), suggesting potentially faster turnover.

Decision Takeaway: Prioritize the stabilizing or neutral variants **P170F, A132I, and V128F** for experimental testing; they improve the electric field by 40–58 % while maintaining or improving thermostability. Avoid highly destabilizing mutations such as V128P (+57 REU) and proline substitutions generally.

Reliability Assessment: Fair. While all 191 replicas passed the Chodera automated equilibration detection scheme and used established force fields (ff14SB+GAFF, TIP3P) at 300 K, the 50 ns production runs are short for full conformational sampling, and single-replica sampling limits statistical confidence. These results are suitable for narrowing the candidate list but insufficient for quantitative activity predictions.

Downloadable Files:

- raw_results.csv: Complete dataset of all 191 variants
- athmt-eti.pdb: Input wild-type structure
- plots/equilibration/ (191 sets): PNG plots and JSON data for temperature, pressure, and total energy convergence assessment

---

Ranked Candidates

Ranking Logic: We first filtered the 191 variants to the most stable 33 % (64 candidates, ddg_fold ≤ ~5.3 REU), then ranked by the magnitude of the electric_field (assuming more negative values indicate stronger catalytically productive fields).

Rank	Mutant	electric_field (MV/cm)	ddg_fold (REU)	Assessment
1	**A132E**	-12.87	+3.95	Strongest field; mildly destabilizing
2	**A132T**	-11.69	+3.16	Strong field; near-neutral stability
3	**V128C**	-11.59	+3.44	Strong field; near-neutral stability
4	**V11F**	-11.20	+4.91	Strong field; borderline stability
5	**P170F**	-10.95	**-0.95**	**Excellent candidate** — stabilizing
6	**A132I**	-10.86	**-0.95**	**Excellent candidate** — stabilizing
7	Y160C	-10.52	+5.30	Good field; marginally stable
8	V11T	-10.45	+5.10	Good field; marginally stable
9	**V128F**	-10.43	**-1.66**	**Strong candidate** — stabilizing
10	V128T	-10.21	+4.43	Good field; moderate stability

WT reference: electric_field = -6.92 MV/cm; ddg_fold = 0.0 REU

Metric-by-Metric Insights

ddg_fold (Rosetta Energy Unit)

- **What it may indicate:** The estimated change in thermodynamic stability of the folded protein relative to WT. Negative values suggest increased melting temperature or resistance to denaturation; positive values warn of potential expression or stability problems.
- **What it does NOT directly prove:** It does not measure catalytic activity, substrate binding affinity, or cellular expression levels. It is a static-structure model estimate, not an experimental melting point.
- **WT/reference pattern:** WT = 0.0 REU.
- **Mutants that stand out:** Highly stabilizing: A132F (-7.0), V128W (-4.7), Y160W (-4.3). Highly destabilizing (exclude): V128P (+57.4), A132P (+35.9), C131R/L/K (+26 to +33).
- **Confidence / caveat:** Fair for flagging large stability changes; small $\Delta\Delta G$ values (< 2 REU) are within typical error margins of single-structure calculations.

electric_field (MV/cm)

- **What it may indicate:** The electrostatic field at the target bond of ETI, generated by the enzyme's internal charge distribution. A stronger aligned field (more negative values in this convention) can polarize the C–I bond, potentially lowering the activation barrier for the SN2 reaction.
- **What it does NOT directly prove:** This is a geometric descriptor from equilibrium charge distributions, not a measured reaction barrier or turnover rate. It does not capture dynamic substrate motions, the precise alignment of the field vector with the bond axis, or solvent reorganization during bond breaking.
- **WT/reference pattern:** -6.92 MV/cm.
- **Mutants that stand out:** A132E nearly doubles the field magnitude (-12.87); A132T/I, V128C/F, and P170F show 50–86 % enhancements. Worrying: V128M (-3.05) and W35F (-3.81) show *weaker* fields than WT.
- **Confidence / caveat:** Fair. Side-chain rotamers may not be fully converged in 50 ns; field directionality (not just scalar magnitude) determines catalytic relevance.

Recommended Follow-Ups

1. **Experimental validation:** Test the top 4 stabilizing, field-enhanced variants (**P170F, A132I, V128F, Y160W**) in vitro for ethyl iodide turnover rates. Start with these four because they combine computational signals of improved catalysis with guaranteed stability.
2. **Enhanced sampling:** Run 200–500 ns simulations (multiple replicas) for the top 5 candidates to verify that the electric field distribution converges and that the active site pocket remains structurally intact over longer timescales.
3. **Mechanistic refinement:** Compute the projection of the electric field vector onto the specific reaction coordinate (the C–I bond axis of ETI) to confirm field alignment is sterically productive, and consider QM/MM free energy perturbations to directly estimate activation barriers for the top candidates.

Appendix — Definitions

- **ddg_fold (Rosetta Energy Unit):** Computational estimate of folding free energy change relative to WT, derived from Rosetta algorithm. Negative = more stable. It is a model-derived prediction, not a direct calorimetric measurement.
- **electric_field (MV/cm):** Computed internal electrostatic field strength at the center of the target substrate bond. Measured in megavolts per centimeter; serves as a *proxy* for catalytic preorganization but is not equivalent to a measured reaction rate constant.

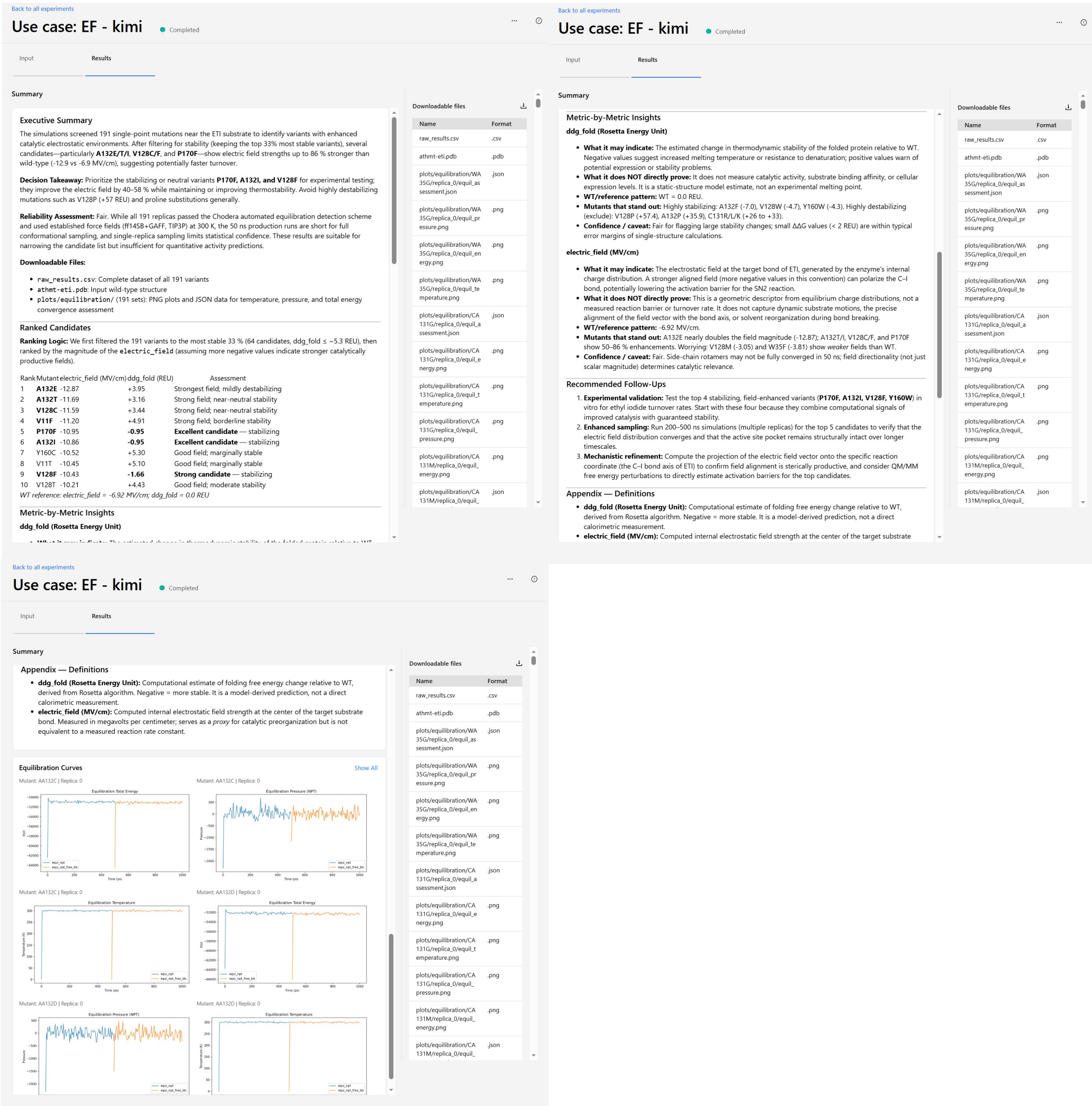

Supplementary Figure 8. Screenshots of the Summary page for use case 3: Non-native Enzyme Activity Engineering using Kimi K2.5. The summary page contains a summary generated by the ResultExplainer agent as final response to the original research question from the user, a list of downloadable source files, and a panel of quality control figures showing the convergence of the simulation.

Supplementary Section 12. The hit rate baseline of the *Aspergillus clavatus* methyltransferase library used in this study.

The initial mutation library has 600 multipoint mutations (as described in Supplementary Section 1), from the screening by Schülke et al[12], the total experimental hits that observed improved multi-specific activity towards bulkier substrates are 66. The baseline hit rate is thus 66/600 = 0.11. And the hit rate improvement is 0.4/0.11=3.6 fold.

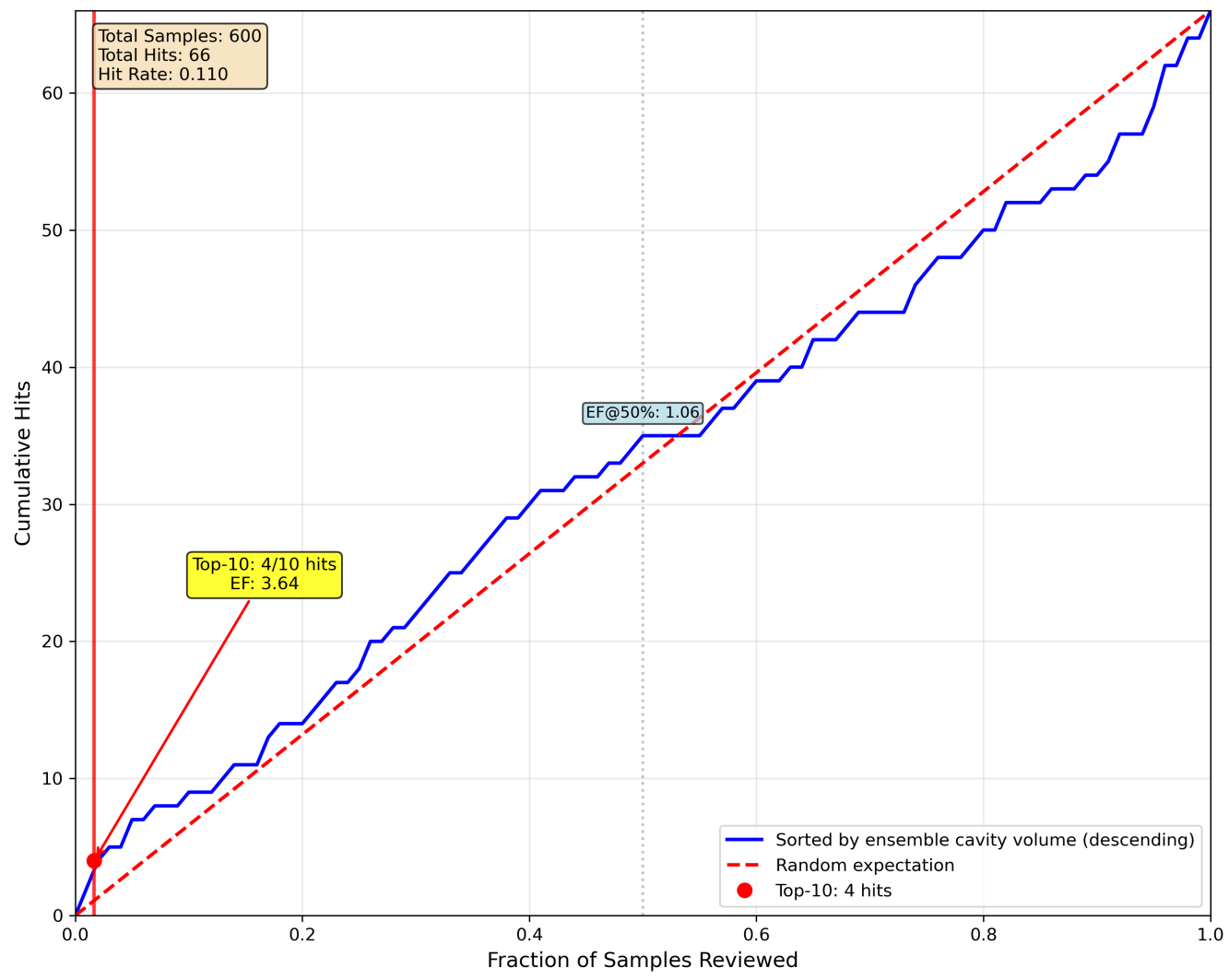

Supplementary Figure 9. The cumulative hit curve for use case 1: Cavity Engineering. (EF stands for enrichment factor) This figure is built by first ranking all samples by their ensemble average cavity volume, then scanning from the top of that ranking and counting how many experimentally confirmed hits have been recovered as screening progresses. The x-axis represents the fraction of samples reviewed, and the y-axis is the cumulative number of hits found up to that point. A random-expectation baseline is included as a reference, where expected hits increase linearly with the reviewed fraction. Enrichment factors at fixed review depths (e.g., 50%) quantify how much better the ranking performs than random selection at early retrieval stages, and a Top-10 summary provides an early-priority metric.

Supplementary Table 1. Metrics MutexaGPT currently supports. This table lists the metrics MutexaGPT currently supports. The related property column shows relevant properties of interest in enzyme engineering campaigns.

Metrics	Type	Related Property
Electric Field Strength along the Bond (electric_field)	Supported	Enzyme activity; transition-state stabilization; chemo-/regio-selectivity (for polarization-sensitive steps); pH-activity profile sensitivity (electrostatic microenvironment)
Substrate Positioning Index (SPI) (spi)	Supported	Enzyme activity; substrate specificity; stereoselectivity/enantioselectivity (when reactive pose geometry controls outcome)

Active Site RMSD (active_site_rmsd)	Supported	Enzyme activity; substrate specificity; substrate scope; mutation-induced functional plasticity; conformational gating effects
MMPB/GBSA Ligand Binding Energy (mmpbgbsa)	Supported	Substrate/cofactor/inhibitor affinity; inhibitor potency; product inhibition tendency; binding-driven specificity/promiscuity tradeoff
Relative Folding Free Energy (ddg_fold)	Supported	Thermostability
Cavity Volume (cavity)	Supported	Substrate scope; promiscuity; selectivity; accommodation of bulky substrates/intermediates; evolvability of active site
Domain Separation Index (DSI) (dsi)	Supported	For bidomain enzymes: enzyme activity change as temperature change; enzyme activity at lower temperature relative to higher temperature

Supplementary Section 13. Topology-based selection of psA linkers.

The ten linkers used in the cold-adaptation engineering case study was randomly selected from the 120 linkers resulted from a topology-based screening from our previous study.[13] In the original study, those 120 linker variants are filtered from 3,528 psA variants by replacing psA's native linker with sequences obtained from the LinkDB[14] and SynLinker databases[15]. The study predicted the structures of these 3,528 psA variants using AlphaFold2, and determined the secondary structure within the linker region using cpptraj with the Dictionary of Secondary Structure of Proteins (DSSP) method. The study then classified psA variants based on their linker topology, emphasizing linker length and the presence of helical motifs. With an average linker length of 9.2 amino acids over 3528 linkers, the study classified linkers longer than 9 amino acids as "long" and the rest as "short". Linkers with a secondary structure featuring a 3-10 helix, α-helix, or π-helix were categorized as "helical", and the rest as "non-helical". Using these two criteria, the study grouped the psA variants into four categories: 1) short and helical, 2) short and non-helical, 3) long and helical, and 4) long and non-helical. The study randomly selected 30 variants from each group, totaling 120 variants.

Supplementary Table 2. Linker sequences used in cold-adaption engineering and their calculated Domain Separation Index (DSI). The table lists the linker sequence, calculated Domain Separation Index (DSI), and variance for each engineered variant and the wild type (WT).

Variant	Linker	DSI (Å)	Variance (Å)
psAt1	IKLYLGYKNKKLRVNINVPMKTE	-0.30	0.10
psAt2	ESPVDSEQRGENDL	-0.08	0.15
psAt3	LEPRVFREVTVKGI	4.66	2.68
psAt4	ENTRGFHG	-1.36	0.09
psAt5	TNGRL	9.48	4.83
psAt6	ITAKDE	20.61	0.19

psAt7	GTLSP	-0.36	0.20
psAt8	FWGMASSSY	0.61	0.17
psAt9	VLATDMSKH	1.23	0.19
psAt10	VKTMADR	3.42	1.44
WT	GSGDGGGNDGGEGGL	5.54	3.36

Supplementary Figure 10. The DSI distribution of the *Pseudomonas saccharophila* amylase wild type and its ten linker variants. (with representative structures for both wild-type peaks) For variants with the largest (psAt6) and smallest (psAt4) average DSI and the wild type, a representative structure of the distribution peak is attached. (grey: catalytic domain, red: linker, dark grey: CBM domain) The linker sequences are noted next to each structure. The kernel density distribution is fitted using the gaussian_kde function from the scipy package. (data obtained from 100 snapshots sampled from the MD trajectory of each variant)

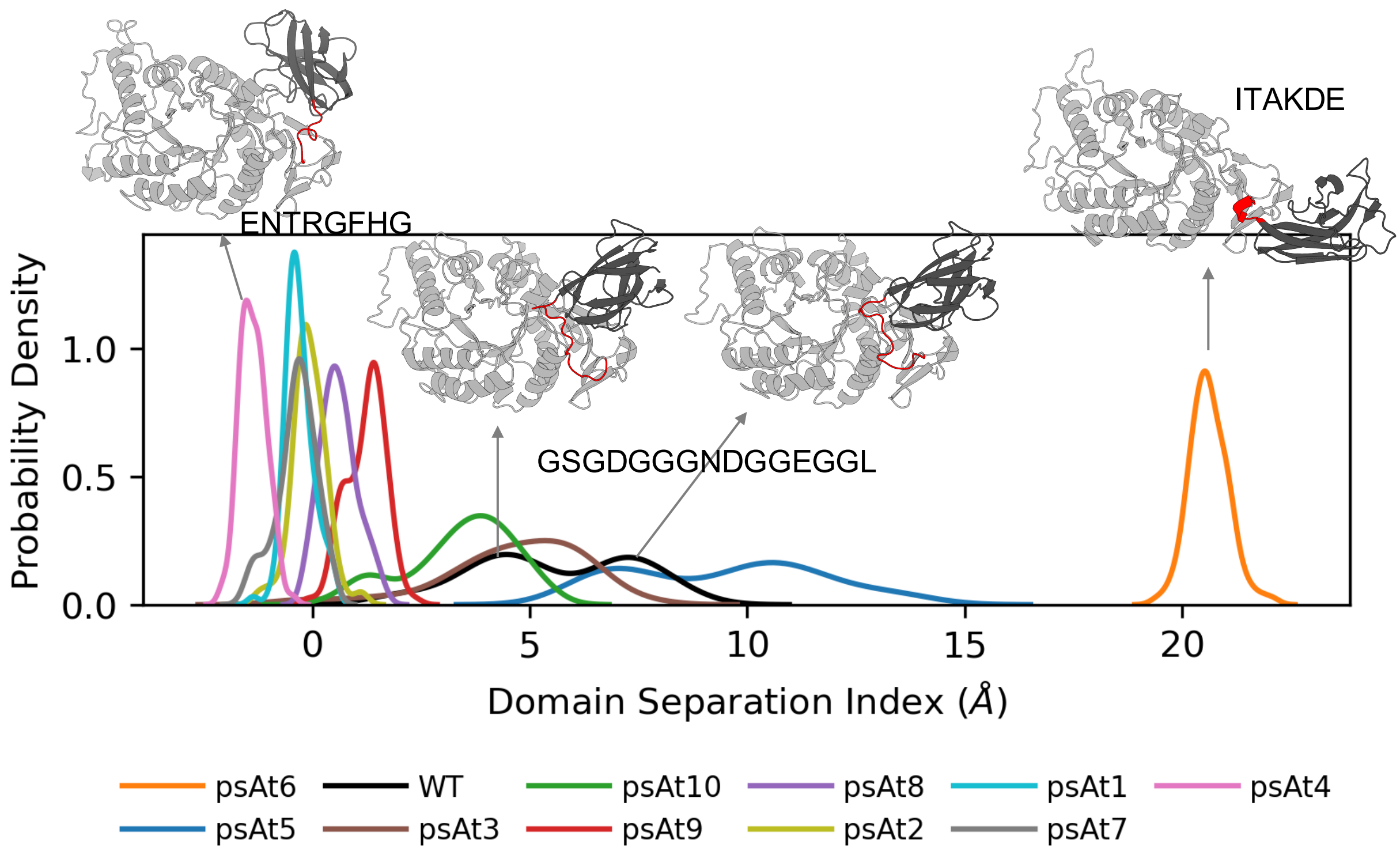

Supplementary Table 3. Amylase activity of psA wild-type, psAt5, and psAt6 under 0 °C and 45 °C. The activity assays were conducted using 0.1 µM enzymes in 15 mM PBS buffer (pH 7.0). One unit of hydrolytic activity was defined as the quantity of enzyme that released reducing sugar equivalent to one µmol of glucose per minute under the assay conditions. Relative activity was quantified as the percentage ratio of enzymatic activity observed at 0 °C to that at the optimal temperature for the enzyme (45 °C). Data are shown as mean ± standard deviation.

variant	Activity (U/mL)		Relative activity (%)
	45°C	0°C	

psAt6	2.86 ± 1.25	0.94 ± 0.05	32.94
psAt5	2.83 ± 0.17	0.43 ± 0.03	15.27
WT	4.14 ± 0.15	0.37 ± 0.08	9.06

Supplementary Table 4. Parameters used for structure prediction using ColabFold server. The table lists the non-default parameter settings used for ColabFold structure prediction. Parameters not listed were set to their default values.

Index	Parameter
num_relax	5
template_mode	pdb100
msa_mode	mmseqs2_uniref_env
pair_mode	unpaired
num_recycles	3

References

1. OpenAI; Hurst, A.; Lerer, A.; Goucher, A. P.; Perelman, A.; Ramesh, A.; Clark, A.; Ostrow, A.; Welihinda, A.; Hayes, A.; Radford, A.; Mądry, A.; Baker-Whitcomb, A.; Beutel, A.; Borzunov, A.; Carney, A.; Chow, A.; Kirillov, A.; Nichol, A.; Paino, A.; Renzin, A.; Tachard Passos, A.; Kirillov, A.; Christakis, A.; Conneau, A.; Kamali, A.; Jabri, A.; Moyer, A.; Tam, A.; Crookes, A.; Tootoochian, A.; Tootoonchian, A.; Kumar, A.; Vallone, A.; Karpathy, A.; Braunstein, A.; Cann, A.; Codispoti, A.; Galu, A.; Kondrich, A.; Tulloch, A.; Mishchenko, A.; Baek, A.; Jiang, A.; Pelisse, A.; Woodford, A.; Gosalia, A.; Dhar, A.; Pantuliano, A.; Nayak, A.; Oliver, A.; Zoph, B.; Ghorbani, B.; Leimberger, B.; Rossen, B.; Sokolowsky, B.; Wang, B.; Zweig, B.; Hoover, B.; Samic, B.; McGrew, B.; Spero, B.; Giertler, B.; Cheng, B.; Lightcap, B.; Walkin, B.; Quinn, B.; Guarraci, B.; Hsu, B.; Kellogg, B.; Eastman, B.; Lugaresi, C.; Wainwright, C.; Bassin, C.; Hudson, C.; Chu, C.; Nelson, C.; Li, C.; Shern, C. J.; Conger, C.; Barette, C.; Voss, C.; Ding, C.; Lu, C.; Zhang, C.; Beaumont, C.; Hallacy, C.; Koch, C.; Gibson, C.; Kim, C.; Choi, C.; McLeavey, C.; Hesse, C.; Fischer, C.; Winter, C.; Czarnecki, C.; Jarvis, C.; Wei, C.; Koumouzelis, C.; Sherburn, D.; Kappler, D.; Levin, D.; Levy, D.; Carr, D.; Farhi, D.; Mely, D.; Robinson, D.; Sasaki, D.; Jin, D.; Valladares, D.; Tsipras, D.; Li, D.; Nguyen, D. P.; Findlay, D.; Oiwoh, E.; Wong, E.; Asdar, E.; Proehl, E.; Yang, E.; Antonow, E.; Kramer, E.; Peterson, E.; Sigler, E.; Wallace, E.; Brevdo, E.; Mays, E.; Khorasani, F.; Petroski Such, F.; Raso, F.; Zhang, F.; von Lohmann, F.; Sulit, F.; Goh, G.; Oden, G.; Salmon, G.; Starace, G.; Brockman, G.; Salman, H.; Bao, H.; Hu, H.; Wong, H.; Wang, H.; Schmidt, H.; Whitney, H.; Jun, H.; Kirchner, H.; Ponde de Oliveira Pinto, H.; Ren, H.; Chang, H.; Chung, H. W.; Kivlichan, I.; O'Connell, I.; O'Connell, I.; Osband, I.; Silber, I.; Sohl, I.; Okuyucu, I.; Lan, I.; Kostrikov, I.; Sutskever, I.; Kanitscheider, I.; Gulrajani, I.; Coxon, J.; Menick, J.; Pachocki, J.; Aung, J.; Betker, J.; Crooks, J.; Lennon, J.; Kiros, J.; Leike, J.; Park, J.; Kwon, J.; Phang, J.; Teplitz, J.; Wei, J.; Wolfe, J.; Chen, J.; Harris, J.; Varavva, J.; Lee, J. G.; Shieh, J.; Lin, J.; Yu, J.; Weng, J.; Tang, J.; Yu, J.; Jang, J.; Quinonero Candela, J.; Beutler, J.; Landers, J.; Parish, J.; Heidecke, J.; Schulman, J.; Lachman, J.; McKay, J.; Uesato, J.; Ward, J.; Kim, J. W. GPT-4o System Card. arXiv:2410.21276, 2024; https://ui.adsabs.harvard.edu/abs/2024arXiv241021276O (accessed October 01, 2024).
2. Gao, S.; Fang, A.; Huang, Y.; Giunchiglia, V.; Noori, A.; Schwarz, J. R.; Ektefaie, Y.; Kondic, J.; Zitnik, M., Empowering biomedical discovery with AI agents. *Cell* **2024**, *187*, 6125-6151.
3. Ramos, M. C.; Collison, C. J.; White, A. D., A review of large language models and autonomous agents in chemistry. *Chem. Sci.* **2025**, *16*, 2514-2572.
4. Xiong, Z.; Lin, Y.; Xie, W.; He, P.; Tang, J.; Lakkaraju, H.; Xiang, Z. How Memory Management Impacts

LLM Agents: An Empirical Study of Experience-Following Behavior. arXiv:2505.16067, 2025; https://ui.adsabs.harvard.edu/abs/2025arXiv250516067X (accessed May 01, 2025).

5. Huang, W.; Xia, F.; Xiao, T.; Chan, H.; Liang, J.; Florence, P.; Zeng, A.; Tompson, J.; Mordatch, I.; Chebotar, Y.; Sermanet, P.; Brown, N.; Jackson, T.; Luu, L.; Levine, S.; Hausman, K.; Ichter, B. Inner Monologue: Embodied Reasoning through Planning with Language Models. arXiv:2207.05608, 2022; https://ui.adsabs.harvard.edu/abs/2022arXiv220705608H (accessed July 01, 2022).

6. Kojima, T.; Gu, S. S.; Reid, M.; Matsuo, Y.; Iwasawa, Y. Large Language Models are Zero-Shot Reasoners. arXiv:2205.11916, 2022; https://ui.adsabs.harvard.edu/abs/2022arXiv220511916K (accessed May 01, 2022).

7. Wei, J.; Wang, X.; Schuurmans, D.; Bosma, M.; Ichter, B.; Xia, F.; Chi, E.; Le, Q.; Zhou, D. Chain-of-Thought Prompting Elicits Reasoning in Large Language Models. arXiv:2201.11903, 2022; https://ui.adsabs.harvard.edu/abs/2022arXiv220111903W (accessed January 01, 2022).

8. Liu, H.; Sferrazza, C.; Abbeel, P. Chain of Hindsight Aligns Language Models with Feedback. arXiv:2302.02676, 2023; https://ui.adsabs.harvard.edu/abs/2023arXiv230202676L (accessed February 01, 2023).

9. Kang, J.; Laroche, R.; Yuan, X.; Trischler, A.; Liu, X.; Fu, J. Think Before You Act: Decision Transformers with Working Memory. arXiv:2305.16338, 2023; https://ui.adsabs.harvard.edu/abs/2023arXiv230516338K (accessed May 01, 2023).

10. Brown, T. B.; Mann, B.; Ryder, N.; Subbiah, M.; Kaplan, J.; Dhariwal, P.; Neelakantan, A.; Shyam, P.; Sastry, G.; Askell, A.; Agarwal, S.; Herbert-Voss, A.; Krueger, G.; Henighan, T.; Child, R.; Ramesh, A.; Ziegler, D. M.; Wu, J.; Winter, C.; Hesse, C.; Chen, M.; Sigler, E.; Litwin, M.; Gray, S.; Chess, B.; Clark, J.; Berner, C.; McCandlish, S.; Radford, A.; Sutskever, I.; Amodei, D. Language Models are Few-Shot Learners. arXiv:2005.14165, 2020; https://ui.adsabs.harvard.edu/abs/2020arXiv200514165B (accessed May 01, 2020).

11. Pokrass, M. Introducing Structured Outputs in the API. https://openai.com/index/introducing-structured-outputs-in-the-api/

12. Schülke, K. H.; Fröse, J. S.; Klein, A.; Garcia-Borràs, M.; Hammer, S. C., Efficient Transferase Engineering for SAM Analog Synthesis from Iodoalkanes. *ChemBioChem* **2024**, *25*.

13. Ding, N.; Jiang, Y.; Ge, R.; Shao, Q.; Shin, W.; Ran, X.; Yang, Z. J., Enhancing Cold Adaptation of Bidomain Amylases by High-Throughput Computational Engineering. *Angew. Chem. Int. Ed.* **2025**, *64*.

14. George, R. A.; Heringa, J., An analysis of protein domain linkers: their classification and role in protein folding. *Protein Engineering, Design and Selection* **2002**, *15*, 871-879.

15. Liu, C.; Chin, J. X.; Lee, D.-Y., SynLinker: an integrated system for designing linkers and synthetic fusion proteins. *Bioinformatics* **2015**, *31*, 3700-3702.
