# Supplementary material for: MutexaGPT: an intuition-to-design translator for physics-based enzyme engineering
Source: Nat Comput Sci. 2026 Sep 10;6(10):1133–46. doi: 10.1038/s43588-026-01049-y (PMC13686939; doi:10.1038/s43588-026-01049-y)
Supplement: Supplementary file 2 [file 43588_2026_1049_MOESM2_ESM.pdf]

nature portfolio

Corresponding author(s): Zhongyue John Yang

Last updated by author(s): Jul 4, 2026

Reporting Summary

Nature Portfolio wishes to improve the reproducibility of the work that we publish. This form provides structure for consistency and transparency in reporting. For further information on Nature Portfolio policies, see our Editorial Policies and the Editorial Policy Checklist.

Statistics

For all statistical analyses, confirm that the following items are present in the figure legend, table legend, main text, or Methods section.

n/a	Confirmed	
☐	☒	The exact sample size (n) for each experimental group/condition, given as a discrete number and unit of measurement
☐	☒	A statement on whether measurements were taken from distinct samples or whether the same sample was measured repeatedly
☒	☐	The statistical test(s) used AND whether they are one- or two-sided<br>*Only common tests should be described solely by name; describe more complex techniques in the Methods section.*
☒	☐	A description of all covariates tested
☒	☐	A description of any assumptions or corrections, such as tests of normality and adjustment for multiple comparisons
☐	☒	A full description of the statistical parameters including central tendency (e.g. means) or other basic estimates (e.g. regression coefficient) AND variation (e.g. standard deviation) or associated estimates of uncertainty (e.g. confidence intervals)
☒	☐	For null hypothesis testing, the test statistic (e.g. F, t, r) with confidence intervals, effect sizes, degrees of freedom and P value noted<br>*Give P values as exact values whenever suitable.*
☒	☐	For Bayesian analysis, information on the choice of priors and Markov chain Monte Carlo settings
☒	☐	For hierarchical and complex designs, identification of the appropriate level for tests and full reporting of outcomes
☒	☐	Estimates of effect sizes (e.g. Cohen's d, Pearson's r), indicating how they were calculated

Our web collection on statistics for biologists contains articles on many of the points above.

Software and code

Policy information about availability of computer code

Data collection	Python 3.9 https://www.python.org/, AMBER22 http://ambermd.org/, EnzyHTP 2.0 https://github.com/ChemBioHTP/EnzyHTP
Data analysis	Python 3.9 https://www.python.org/, AMBER22 http://ambermd.org/, PYMOL 3.1, EnzyHTP 2.0 https://github.com/ChemBioHTP/EnzyHTP

For manuscripts utilizing custom algorithms or software that are central to the research but not yet described in published literature, software must be made available to editors and reviewers. We strongly encourage code deposition in a community repository (e.g. GitHub). See the Nature Portfolio guidelines for submitting code & software for further information.

Data

Policy information about availability of data

All manuscripts must include a data availability statement. This statement should provide the following information, where applicable:

- Accession codes, unique identifiers, or web links for publicly available datasets
- A description of any restrictions on data availability
- For clinical datasets or third party data, please ensure that the statement adheres to our policy

The original input file for all involved MD simulations and full LLM-agent evaluation results are available from Zenodo (https://doi.org/10.5281/zenodo.21197440). The MutexaGPT webapp is available at https://enzyhtp.app.vanderbilt.edu/. The source code of MutexaGPT and the evaluation system of LLM-agents in MutexaGPT is available from https://github.com/ChemBioHTP/EnzyHTP-GPT/. (DOI: 10.5281/zenodo.21197663; OpenAI Codex was used as an AI coding assistant during the development of MutexaGPT, and all final code was reviewed and curated by the authors.)

March 2021

Human research participants

Policy information about studies involving human research participants and Sex and Gender in Research.

Reporting on sex and gender	N/A
Population characteristics	N/A
Recruitment	N/A
Ethics oversight	N/A

Note that full information on the approval of the study protocol must also be provided in the manuscript.

Field-specific reporting

Please select the one below that is the best fit for your research. If you are not sure, read the appropriate sections before making your selection.

☒ Life sciences ☐ Behavioural & social sciences ☐ Ecological, evolutionary & environmental sciences

For a reference copy of the document with all sections, see nature.com/documents/nr-reporting-summary-flat.pdf

Life sciences study design

All studies must disclose on these points even when the disclosure is negative.

Sample size	No a priori sample size calculation was performed. Sample sizes were chosen based on predefined computational coverage, available variant libraries, and practical proof-of-concept validation. LLM benchmarks used balanced curated test sets to cover relevant input classes. The cavity-engineering study simulated all 600 variants in an existing experimentally characterized library.<br>600 combinatorial mutations of Aspergillus clavatus methyltransferase are from mutating residue 11 to A, F, I, or L; residue 30 to C, F, I, or M; residue 31 to W; residue 39 to H, D, I, E, or T; residue 41 to F. Each point can either be mutated or not mutated.<br>The linker-engineering study evaluated WT and 10 predefined linker variants, with experimental validation of the two top-ranked variants. Biochemical assays used three biologically independent replicates per construct and temperature to estimate mean and standard deviation.<br>The 10 Pseudomonas saccharophila amylase linker variants are selected based on linker topology from 120 linkers from a previous study of ours. (Supplementery Section 7)
Data exclusions	No data were excluded from the analyses.
Replication	Replication can be done following the experimental methods described in the main text. All attempts at replication were successful.
Randomization	Randomization was not relevant to the computational experiments because the study did not involve allocation of subjects to treatment groups
Blinding	Blinding was not relevant to the computational experiments because the study did not involve allocation of subjects to treatment groups; enzyme variants, prompt designs and simulation inputs were predefined, and analyses were performed using automated workflows and predefined criteria. For the biochemical validation assays, investigators were aware of enzyme identity because the WT and designed variants were expressed and assayed as predefined constructs.

Reporting for specific materials, systems and methods

We require information from authors about some types of materials, experimental systems and methods used in many studies. Here, indicate whether each material, system or method listed is relevant to your study. If you are not sure if a list item applies to your research, read the appropriate section before selecting a response.

Materials & experimental systems

n/a	Involved in the study
☒	☐ Antibodies
☒	☐ Eukaryotic cell lines
☒	☐ Palaeontology and archaeology
☒	☐ Animals and other organisms
☒	☐ Clinical data
☒	☐ Dual use research of concern

Methods

n/a	Involved in the study
☒	☐ ChIP-seq
☒	☐ Flow cytometry
☒	☐ MRI-based neuroimaging

March 2021
