# Supplementary material for: MutexaGPT: an intuition-to-design translator for physics-based enzyme engineering
Source: Nat Comput Sci. 2026 Sep 10;6(10):1133–46. doi: 10.1038/s43588-026-01049-y (PMC13686939; doi:10.1038/s43588-026-01049-y)
Supplement: Supplementary file 3 [file 43588_2026_1049_MOESM3_ESM.pdf]

nature portfolio

Peer Review File

MutexaGPT: An Intuition-to-Design Translator for Physics-based Enzyme Engineering

Corresponding Author: Professor Zhongyue Yang

Version 0:

Reviewer comments:

Reviewer #1

(Remarks to the Author)
Co-reviewed with Reviewer #2
Review Report for manuscript NATCOMPUTSCI-25-4365

The manuscript “MutexaGPT: An Intuition-to-Design Translator for Physics-based Enzyme Engineering” by Shao et al. presents a multi-LLM agent web-based platform for in silico enzyme design/engineering campaigns. The main objective of this work is to enable non-computational experts to execute high-throughput computational simulations that target an intuition-guided initial prompt. To accomplish this, MutexaGPT was designed with three main agents: QuestionAnalyzer, WorkPlanningBoard, and ResultExplainer. The performance of these agents, with particular focus on the QuestionAnalyzer, is assessed using an automated LLM-based benchmarking framework that included curated prompts and classification of results in terms of relevance and completeness to ensure the LLM operates as intended. The authors present two case studies to highlight how MutexaGPT can be used. This work is likely to be high impact with broad interest to the field of enzyme engineering/design for non-computational researchers who would be interested in using the platform, as well as scientists who may be interested in developing LLM agents for their own computational programs. However, we believe there are some revisions required to ensure MutexaGPT is production-ready.

Major Comments:
1. The authors should make it more clear to the non-computational expert what each computable metric could correspond to. For example, (although MutexaGPT does not automate receptor-ligand docking simulations) docking simulations calculate G-scores, not binding affinities, so distinctions like this should be made abundantly clear to the non-computational expert to avoid misinterpretation of results. Line 194 says one goal of the ResultExplainer agent is to “supply just enough domain background so non-experts grasp why a result matters”, but no data is included in the manuscript as evidence this happens. The raw ResultExplainer responses for the two case studies should be included in the SI and discussed in the manuscript.
2. We really appreciate the effort behind assessing the QuestionAnalyzer LLM agent performance. For this work to be of value to the community, it is essential that it operates as intended. However, the current information regarding the performance evaluation is not enough for us to determine if MutexaGPT operates as intended, for a few reasons:
2.1. The authors explain that a set of test inquiries were generated (by an LLM) and assigned attributes/labels corresponding to their relevance and completeness. The SI Figure S2 indicates the TestSetCurator may have included some “human correction” to generate the test questions and corresponding labels, whereas in the main text it says the labels were “compared with manually verified ground-truth labels” (line 216, among other places). The authors should include this entire test set in the SI, along with their labels, and performance assessment of each question with the different MutexaGPT agents (and corresponding prompt variations). This transparency is mandatory to ensure the evaluation spans an appropriate domain of applicability such that MutexaGPT operates as intended.
2.2. Only 20/120 inquiries were “irrelevant”, and the Full QuestionAnalyzer prompt design performance for relevance was 96.7%. Our understanding is that corresponds to 116 inquiries were correctly identified as either relevant or irrelevant, but details beyond that are lacking. Since only ~17% of the test data was “irrelevant”, the design of this evaluation is skewed, especially if the prompt design is more likely to consider an irrelevant inquiry as relevant (rather than more likely to consider a relevant inquiry as irrelevant). This test should be designed with less bias so it can be more accurately assessed. Furthermore, presentation of the performance results for each inquiry will help us understand if the prompt design has any bias. We believe this is very important because if MutexaGPT is not able to detect an irrelevant inquiry accurately, this could potentially lead to wasted computational time (and thus wasted resources).
2.3. The performance percentages in Figure 2B do not make sense. If there are 120 test inquiries and no possibility for “partial” pass, then the percentages of 99.4, and 97.2 are not possible. Maybe our interpretation of how this performance is calculated is wrong. If not, the authors should carefully review all performance data.
2.4. The authors should include a discussion about why certain inquiries failed at certain points.

2.5. Line 226: Please elaborate on what is meant by “behave correctly”. Were the missing pieces of information in under-specified explicitly labeled, and assessed if that corresponded to the QuestionAnalyzer request? How was correctness determined?
2.6. More information regarding the performance of other agents is warranted (in addition to providing the specific inputs used to assess their performance and definition of fails/passes). In the paper, only the performance of QuestionAnalyzer is discussed in detail, whereas that of the others is just mentioned that it was completed. For example, the agent MetricsPlanner has a success rate of 91%. What goes wrong for it to fail 9% of the time? This one seems very crucial to get correct. Furthermore, MutationPlanner has a 100% success rate. Is this because in all the test cases the exact mutations were provided?
2.7. What is considered “failed” in MetricsPlanner? Is each EnzyHTP computable metric represented in the test inquiries? To accomplish the main objective, it is paramount that MutexaGPT translates the intuition into an appropriate calculatable property. Therefore, this performance must be critically assessed.
3. The results in the MutexaGPT platform examples do not show any statistics, even though replicate simulations were performed. What was the length of equilibration for the MD simulations performed? What analysis was used to confirm that the system has equilibrated? This type of information is always necessary to include when discussing simulation results, so it would be valuable to display this information in the “executive summary” section of the MutexaGPT web platform, and included in the ResultExplainer agent.
4. The reported “hit rate” for the cavity size example appears cherry-picked (e.g., top 10 variants). Rank-based metrics (e.g., Spearman’s correlation across all variants, or at least the 66 experimental “ground-truth” hits) would be more appropriate. We understand the objective of this paper is not to optimize these two enzymes, therefore the success of these campaigns is not critical to this manuscript. Rather, these examples showcase what MutexaGPT could help automate. In line with this, these results sections should include more specifics on what the MutexaGPT outputs were, such as including a screen shot of the MutexaGPT ResultsExplainer output in the SI. Line 325 says “MutexaGPT’s top-ranked mutations”, which is a little misleading, since those are the top ranks from the simulation data (MutexaGPT did not provide any inference; it is just displaying the simulation results in natural-language, to our understanding). Limitations, especially those that a non-computational expert would be unaware of, should be discussed in these example sections and explained by the ResultsExplainer (e.g., cavity size as a descriptor, force-field choice, simulation length, solvent model, etc.). It would also be useful to discuss whether ResultExplainer could suggest methodological improvements, such as performing longer simulations on top-ranked variants.
5. It was not discussed what the workflow would look like if the intuition does not correspond to an EnzyHTP computable metric, which we believe is important to address. Would this count as “irrelevant” and be accurately flagged by MutexaGPT? Are there examples of these scenarios in the performance benchmarking? Overall, the limitations of MutexaGPT (and inherently, the limitations of EnzyHTP), should be identified in the discussion. Paragraph starting at line 506 would be appropriate to clarify this.

Minor Comments:
6. If a non-computational researcher used MutexaGPT and then wanted to publish a manuscript, will the output provided by MutexaGPT be able to supply reproducible methods? This is already a concern in the field. Some discussion on this should be included, because non-computational experts cannot be expected to know what level of detail is required for simulation method reproducibility.
7. In the introduction and discussion, it should be made clearer that MutexaGPT is an automation tool, not a predictive tool. That is, it translates the “intuition” enclosed in the user’s question to a metric (or multiple metrics) that can be computed from simulation, but the available metrics are hard coded into the system (EnzyHTP capabilities as well as the MetricPlanning LLM Agent prompt).
8. Section 2.2 was challenging to follow. Some of the content in the SI should be moved to the main text for better clarity. An introduction into the fact that LLM agents need to be given a prompt would be beneficial to the reader to make it clear that this section assesses the performance based on how the agents were designed (rather than performance of an enzyme design study, for example). Furthermore, it should be made very clear that the “prompt design” is the prompt given to the agent, and not the prompt that is provided by the user (aka the inquiry/question/input). Explanation of all different designs (CoT scaffold, ICL, among others) should be moved to the main text.
9. Will the LLM retain information provided by the user? Is there any potential issue with data protection?
10. The purpose of the survey is unclear and does not seem directly relevant to the main objective of the paper. Consider moving all survey-related content to the SI. Perhaps the authors could estimate how long it took the user to interact with MutexaGPT in the two examples and compare that time with how much time it takes for the average person to set up an MD simulation.
11. Line 256: “second” was already used. Should be “third”
12. Regarding section 2.2: Is the performance good in the general LLM context? Are there any references to more LLM specific research to suggest this is a good performance? Include discussion on potential pitfalls of using the full prompt (i.e., could this cause the agent to be restricted?)
13. Line 335: “moderate MD length” is a very domain specific and ambiguous term. Does MutexaGPT know if a simulation length is short vs moderate? Moreover, in line 575, it is mentioned that “MD simulation time was set to 50 ns”. Is this information that the non-expert user is expected to provide, or is there a default value? And are there any fail-safes to the simulations? If it blows up, how is it communicated to the non-expert? Examples of this would be very valuable.
14. Regarding the discussion paragraph beginning at line 524, we believe MutexaGPT should be classified at the assistant-level currently, particularly given that the MetricPlanner LLM agent prompt clearly guides what metric each “intuition” corresponds to. The evaluations that were performed do not indicate that a collaborator level has been reached, but the authors could consider different evaluations to assess this more quantitatively and thus strengthen their argument that MutexaGPT is beyond the assistant level.

(Remarks on code availability)
The code (specifically the web-based GUI) is a usable resource for the community. Our manuscript assessment (see comments to authors) explains that more information about the code performance is necessary to illustrate proper functionality of the code.

Reviewer #2

(Remarks to the Author)
Co-reviewed with Reviewer #1
Review Report for manuscript NATCOMPUTSCI-25-4365

The manuscript "MutexaGPT: An Intuition-to-Design Translator for Physics-based Enzyme Engineering" by Shao et al. presents a multi-LLM agent web-based platform for in silico enzyme design/engineering campaigns. The main objective of this work is to enable non-computational experts to execute high-throughput computational simulations that target an intuition-guided initial prompt. To accomplish this, MutexaGPT was designed with three main agents: QuestionAnalyzer, WorkPlanningBoard, and ResultExplainer. The performance of these agents, with particular focus on the QuestionAnalyzer, is assessed using an automated LLM-based benchmarking framework that included curated prompts and classification of results in terms of relevance and completeness to ensure the LLM operates as intended. The authors present two case studies to highlight how MutexaGPT can be used. This work is likely to be high impact with broad interest to the field of enzyme engineering/design for non-computational researchers who would be interested in using the platform, as well as scientists who may be interested in developing LLM agents for their own computational programs. However, we believe there are some revisions required to ensure MutexaGPT is production-ready.

Major Comments:
1. The authors should make it more clear to the non-computational expert what each computable metric could correspond to. For example, (although MutexaGPT does not automate receptor-ligand docking simulations) docking simulations calculate G-scores, not binding affinities, so distinctions like this should be made abundantly clear to the non-computational expert to avoid misinterpretation of results. Line 194 says one goal of the ResultExplainer agent is to "supply just enough domain background so non-experts grasp why a result matters", but no data is included in the manuscript as evidence this happens. The raw ResultExplainer responses for the two case studies should be included in the SI and discussed in the manuscript.
2. We really appreciate the effort behind assessing the QuestionAnalyzer LLM agent performance. For this work to be of value to the community, it is essential that it operates as intended. However, the current information regarding the performance evaluation is not enough for us to determine if MutexaGPT operates as intended, for a few reasons:
2.1. The authors explain that a set of test inquiries were generated (by an LLM) and assigned attributes/labels corresponding to their relevance and completeness. The SI Figure S2 indicates the TestSetCurator may have included some "human correction" to generate the test questions and corresponding labels, whereas in the main text it says the labels were "compared with manually verified ground-truth labels" (line 216, among other places). The authors should include this entire test set in the SI, along with their labels, and performance assessment of each question with the different MutexaGPT agents (and corresponding prompt variations). This transparency is mandatory to ensure the evaluation spans an appropriate domain of applicability such that MutexaGPT operates as intended.
2.2. Only 20/120 inquiries were "irrelevant", and the Full QuestionAnalyzer prompt design performance for relevance was 96.7%. Our understanding is that corresponds to 116 inquiries were correctly identified as either relevant or irrelevant, but details beyond that are lacking. Since only ~17% of the test data was "irrelevant", the design of this evaluation is skewed, especially if the prompt design is more likely to consider an irrelevant inquiry as relevant (rather than more likely to consider a relevant inquiry as irrelevant). This test should be designed with less bias so it can be more accurately assessed. Furthermore, presentation of the performance results for each inquiry will help us understand if the prompt design has any bias. We believe this is very important because if MutexaGPT is not able to detect an irrelevant inquiry accurately, this could potentially lead to wasted computational time (and thus wasted resources).
2.3. The performance percentages in Figure 2B do not make sense. If there are 120 test inquiries and no possibility for "partial" pass, then the percentages of 99.4, and 97.2 are not possible. Maybe our interpretation of how this performance is calculated is wrong. If not, the authors should carefully review all performance data.
2.4. The authors should include a discussion about why certain inquiries failed at certain points.
2.5. Line 226: Please elaborate on what is meant by "behave correctly". Were the missing pieces of information in under-specified explicitly labeled, and assessed if that corresponded to the QuestionAnalyzer request? How was correctness determined?
2.6. More information regarding the performance of other agents is warranted (in addition to providing the specific inputs used to assess their performance and definition of fails/passes). In the paper, only the performance of QuestionAnalyzer is discussed in detail, whereas that of the others is just mentioned that it was completed. For example, the agent MetricsPlanner has a success rate of 91%. What goes wrong for it to fail 9% of the time? This one seems very crucial to get correct. Furthermore, MutationPlanner has a 100% success rate. Is this because in all the test cases the exact mutations were provided?
2.7. What is considered "failed" in MetricsPlanner? Is each EnzyHTP computable metric represented in the test inquiries? To accomplish the main objective, it is paramount that MutexaGPT translates the intuition into an appropriate calculatable property. Therefore, this performance must be critically assessed.
3. The results in the MutexaGPT platform examples do not show any statistics, even though replicate simulations were performed. What was the length of equilibration for the MD simulations performed? What analysis was used to confirm that the system has equilibrated? This type of information is always necessary to include when discussing simulation results, so it would be valuable to display this information in the "executive summary" section of the MutexaGPT web platform, and

included in the ResultExplainer agent.
4. The reported “hit rate” for the cavity size example appears cherry-picked (e.g., top 10 variants). Rank-based metrics (e.g., Spearman’s correlation across all variants, or at least the 66 experimental “ground-truth” hits) would be more appropriate. We understand the objective of this paper is not to optimize these two enzymes, therefore the success of these campaigns is not critical to this manuscript. Rather, these examples showcase what MutexaGPT could help automate. In line with this, these results sections should include more specifics on what the MutexaGPT outputs were, such as including a screen shot of the MutexaGPT ResultsExplainer output in the SI. Line 325 says “MutexaGPT’s top-ranked mutations”, which is a little misleading, since those are the top ranks from the simulation data (MutexaGPT did not provide any inference; it is just displaying the simulation results in natural-language, to our understanding). Limitations, especially those that a non-computational expert would be unaware of, should be discussed in these example sections and explained by the ResultsExplainer (e.g., cavity size as a descriptor, force-field choice, simulation length, solvent model, etc.). It would also be useful to discuss whether ResultExplainer could suggest methodological improvements, such as performing longer simulations on top-ranked variants.
5. It was not discussed what the workflow would look like if the intuition does not correspond to an EnzyHTP computable metric, which we believe is important to address. Would this count as “irrelevant” and be accurately flagged by MutexaGPT? Are there examples of these scenarios in the performance benchmarking? Overall, the limitations of MutexaGPT (and inherently, the limitations of EnzyHTP), should be identified in the discussion. Paragraph starting at line 506 would be appropriate to clarify this.

Minor Comments:
6. If a non-computational researcher used MutexaGPT and then wanted to publish a manuscript, will the output provided by MutexaGPT be able to supply reproducible methods? This is already a concern in the field. Some discussion on this should be included, because non-computational experts cannot be expected to know what level of detail is required for simulation method reproducibility.
7. In the introduction and discussion, it should be made clearer that MutexaGPT is an automation tool, not a predictive tool. That is, it translates the “intuition” enclosed in the user’s question to a metric (or multiple metrics) that can be computed from simulation, but the available metrics are hard coded into the system (EnzyHTP capabilities as well as the MetricPlanning LLM Agent prompt).
8. Section 2.2 was challenging to follow. Some of the content in the SI should be moved to the main text for better clarity. An introduction into the fact that LLM agents need to be given a prompt would be beneficial to the reader to make it clear that this section assesses the performance based on how the agents were designed (rather than performance of an enzyme design study, for example). Furthermore, it should be made very clear that the “prompt design” is the prompt given to the agent, and not the prompt that is provided by the user (aka the inquiry/question/input). Explanation of all different designs (CoT scaffold, ICL, among others) should be moved to the main text.
9. Will the LLM retain information provided by the user? Is there any potential issue with data protection?
10. The purpose of the survey is unclear and does not seem directly relevant to the main objective of the paper. Consider moving all survey-related content to the SI. Perhaps the authors could estimate how long it took the user to interact with MutexaGPT in the two examples and compare that time with how much time it takes for the average person to set up an MD simulation.
11. Line 256: “second” was already used. Should be “third”
12. Regarding section 2.2: Is the performance good in the general LLM context? Are there any references to more LLM specific research to suggest this is a good performance? Include discussion on potential pitfalls of using the full prompt (i.e., could this cause the agent to be restricted?)
13. Line 335: “moderate MD length” is a very domain specific and ambiguous term. Does MutexaGPT know if a simulation length is short vs moderate? Moreover, in line 575, it is mentioned that “MD simulation time was set to 50 ns”. Is this information that the non-expert user is expected to provide, or is there a default value? And are there any fail-safes to the simulations? If it blows up, how is it communicated to the non-expert? Examples of this would be very valuable.
14. Regarding the discussion paragraph beginning at line 524, we believe MutexaGPT should be classified at the assistant-level currently, particularly given that the MetricPlanner LLM agent prompt clearly guides what metric each “intuition” corresponds to. The evaluations that were performed do not indicate that a collaborator level has been reached, but the authors could consider different evaluations to assess this more quantitatively and thus strengthen their argument that MutexaGPT is beyond the assistant level.

(Remarks on code availability)
MutexaGPT was accessed through the website and I logged into it using the credentials provided in the paper. Overall, it seems quite user-friendly, however, more details about the statistical aspects of the replica MD simulations would be better.

Reviewer #3

(Remarks to the Author)
The authors describe an agentic framework “MutexaGPT” for LLM-powered text-based automation of their previously developed automated simulation framework “EnzyHTP.” This links physical intuition to high-throughput simulation and analysis. The system is intended for users with expertise in enzyme engineering, but little to no molecular modeling expertise. Two specific and straightforward enzyme designs are demonstrated, including experimental validation. Strong claims regarding utility are made that are not well supported by the data. As currently written, there are several major deficiencies in the evaluation and reporting of results, including:
Major:

1. The authors present a survey with no methodological details, (ie. number of subjects, response rates) and the manuscript lacks a formal statement regarding ethical oversight (ie. consent / anonymization). Also, a median of 2 days is reported though it is not provided as a discrete option within the survey.

2. The utility for enzyme design is overstated in the abstract and introduction. The capacities of MutexaGPT carry at least the limitations of EnzyHTP. The statement “hypothesis refinement is still limited to correcting non-scientific statements, retrieving missing information for a vague request, and mapping general properties of interest to computable metrics” (page 27) clearly defines the scope of the contribution and should be emphasized earlier on, while more exaggerated claims should be tempered.

3. The design capacity is linked to the capacity for (high throughput) MD observables to be directly translated to enzyme design. This is only straightforward for a number of use cases such as increasing volume or distance. It is unclear how the agent will respond or what the hit-rate will be in examples where there is not a clear correlation between MD variable and desired activity change.

4. Use is intended for non-molecular modeling experts. However, 30 us of simulation requires substantial computational resources. It is unclear whether the web-app has the capacity to host these or whether additional resources are needed. If this app is capable of supporting, are the benchmarks for the time it will take to run the simulations?

5. The performance reported in figure 2B is difficult to interpret without knowledge of how challenging or diverse the discrimination tasks were. It is also not clear how these evaluation metrics compare to other state-of-the-art LLM-based agents.

Minor:

6. The SI is disorganized with Text / Figures / Tables interspersed, and the large text boxes are particularly long and difficult to scroll through.

7. Citations are missing on page 27 for the sentence “Existing LLM-agentic molecular simulation tools mostly operate at the assistant level, designed to streamline the molecular modeling process for domain experts.”

8. Computational method page 30 mentions a “search through rotamer library to find the optimal side-chain position,” however methodological details necessary for reproducibility are not provided.

(Remarks on code availability)
No apparent issues

Reviewer #4

(Remarks to the Author)
Shao et al. present MutexaGPT, a web server that provides a natural language interface to make enzyme engineering informed by molecular dynamics (MD) simulations accessible to non-expert users. The multi-agent system, running on the now-discontinued GPT-4o large language model, processes a user's request by analyzing the user's intent, selecting appropriate metric(s) for the intended improvement, executing MD-based pipelines, and evaluating their results. The tool's capabilities are tested on two relevant enzyme engineering tasks: active-site enlargement to accommodate bulkier substrates and improving enzyme performance at lower temperatures. While the tool successfully identified improved enzyme variants in both cases, its success largely depends on the capability of MD-based methods to provide meaningful metrics that correspond to the user's intent. Additionally, the fast pace at which large language models are developed and commercial models are discontinued raises questions about the life-cycle of the presented tool. Nevertheless, MutexaGPT has the potential to become a meaningful addition to the computational enzyme engineering toolkit, by significantly lowering the barrier of entry to complex MD-based pipelines to a level that they become accessible to enzyme engineers with little to no previous experience in computational methods. Beyond the technical advancements, the ease of use benefits from an intuitive user interface and the provision of computational resources for computationally heavy simulations, both of which I strongly acknowledge. I would like the authors to address the following concerns:

Major Concerns:

- All experiments presented in the manuscript were obtained with the large language model (LLM) GPT-4o from OpenAI, which has been recently discontinued and is no longer publicly available, making the results irreproducible. Even though I don't think this drastically affects the usefulness of the presented tool, it raises a concern the authors mention themselves as the final statement of the manuscripts: AI models are developing at a fast pace and fully closed models cannot be seen as a long-lasting resource available to researchers. This necessitates benchmarking the system's performance across different LMMs in the backend. I recommend to analyse the performance with at least one comparable LLM with open-weigths. Specifically I would like to see the performance of the prompts with an open-weights model and how it would perform for the computational site in the two design campaigns because they will remain accessible in the future.

- The authors claim multiple times that the presented tool helps to transform "physical-intuition" into "actionable insights" for enzyme engineering and claim to demonstrate this with the two enzyme engineering campaigns presented in the manuscripts. However, in both campaigns the tool is provided with a very specific set of mutations/linkers for which the tool should perform simulations. While the authors state that providing this information is optional or could be more coarse-grained, the performance of the tool in such a scenario remains unclear. To really see how the tool transforms the intuition of the user, the performance of the tool in the two engineering campaigns should be investigated with less information on the selected mutations upfront (e.g. define position only by distance from ligand). Alternatively, the manuscript should be heavily rephrased to avoid the impression that the tool is capable of running experiments without any prior knowledge of the activity at hand. Without the experiment of letting the tool identify potential mutations on its own, I don't see any notion of "intuition to actionable insights" as justified.

- Even though I generally think the manuscript is well written, it contains multiple lurid claims that are not supported by the data. It's important to point out that the tool can help in identifying mutants with improved properties only if MD-based metrics can provide meaningful insight into the property of interest. For example, the cavity engineering campaign recovered only 4 of the 10 best-performing mutants, and the data suggest that this is due to fundamental limitations of the MD-derived cavity volume metric in fully capturing differences in activity across substrates. Unfortunately, this limitation is not discussed in the manuscript. Rather, the tool's capabilities are displayed to "enable a new paradigm for enzyme engineering where human creativity is seamlessly integrated with computational simulation to make new discoveries and designs" (L503f), which is simply overselling the tool's capability given the presented data. I would like the authors to reflect more carefully on the limitations of the tools and recommend revising the following (but not limited) sections of the manuscript: L122-125, L279f., L351-L353, L502-L505.

- I recommend including an overview of all metrics supported in the current version of the tool. Further, the tool's usefulness depends heavily on the metrics' usefulness for engineering campaigns. I recommend discussing this relation in more depth based on the existing literature and focusing less on metrics that could be added in the future, which, at this moment, can only be treated as speculative.

- The analysis of the tools' performance with different prompt designs needs some clarification on the how it was evaluated. L216-218 states that the performance was tested across three runs. To what does "run" refer here? Was the agent prompted three times with the same question and evaluated three times by the evaluator LLM? Or do runs refer to successfully executed MD tool calls? This section and the corresponding section in the supplementary material would benefit from a more detailed explanation.

- It should be pointed out clearly that using the tool requires a paid OpenAI API key

Minor Concerns:

- The survey results (Figure 1A, Figure S3) are likely not representative and should be removed. I think readers of this paper will be aware of the steep learning curve MD simulations come along with, even without the results of the survey. I trust the main conclusions of the survey, but I fear the survey results could be cited in the future, even though the survey design might not reflect the state of the art and might not be representative.

- I recommend to cite Ai.zymes (https://doi.org/10.1002/anie.202507031) as another example of an automated pipeline that relies on MD-based metrics for user-friendly enzyme engineering, and DynaMate as an AI agent for preparing and running MD simulations for protein-ligand systems (https://doi.org/10.48550/arXiv.2512.10034)

- For the cavity engineering campaign, I believe another important comparison to understand the necessity of MD simulations would be co-folding of the mutated sequences and ligands with AlphaFold3 or Boltz2 and measuring the change in cavity volume in the predicted structures.

- For the experimental data of the cold adaptation campaign, I recommend including a plot that shows the unnormalised activity of the wildtype and tested variants in the supplementary information

- I appreciate the option to run the computationally heavy simulations through the webapp, while also providing the option to download scripts for running simulations locally. I was wondering if there are any limitations on the number of simulations or the simulation length. If so, it would be useful to state them in the manuscript and the webapp.

- I could not find any information on how the data the user uploads to the webapp is processed with regard to privacy. I would recommend to include information on this in the webapp

- The code repository is missing a licence that defines if and under which conditions someone is allowed to use the software. Any type of licence should be added to avoid confusion.

- The experimental results mentioned in L346-L350 miss a citation from which they are derived.

- I recommend making the dataset from the prompt evaluation experiment publicly available to allow for custom prompt engineering of users with automated methods (e.g. GEPA)

- The webapp has many Icons corresponding to features under construction. Will these actually be implemented in the future?

(Remarks on code availability)
Unfortunately I was not able to run the code or use the tool through the webapp, as it requires a paying for access to the GPT models from OpenAI.

The general documentation of the code for setting up the web server seems appropriate. However, the main contribution lies within the multi-LLM system. The code for this section could benefit from better documentation that should be pointed out in the main README of the repository.

Version 1:

Reviewer comments:

Reviewer #1

(Remarks to the Author)
All raised points have been satisfactorily addressed. No further comments.

(Remarks on code availability)

Reviewer #2

(Remarks to the Author)
All raised comments were satisfactorily addressed. No further comments.

(Remarks on code availability)

Reviewer #3

(Remarks to the Author)
The authors have thoroughly addressed the majority of my previous concerns. A few minor comments remain:

1) MutexaGPT runs several software
"interface modules to common software such as Amber83 for trajectory analysis, Gaussian84/xTB85
for quantum mechanics, Multiwfn86 for wavefunction analysis, Rosetta87 for biophysics, etc." some of which require a license to run. Are the authors certain they are not violating terms of any of these by freely enabling others to run their programs through MutexaGPT? Do individuals need to confirm they have their own license, or will the program simply not run and output scripts in certain cases?

2) If input scripts are output rather than using the server, this could be a challenge for non-computationalists. Different servers have different queue's / syntax / installed software. It is not clear how this function will actually be utilized.

3) The authors should state more clearly that the first test case is a retrospective validation while only the second is prospectively experimentally validated.

4) If significant portions of the code were written by AI coding assistants, the authors should disclose this.

(Remarks on code availability)

Reviewer #4

(Remarks to the Author)
I highly appreciate the extensive additions and evaluations in the revised manuscript. The authors have fully addressed my previous concerns regarding long-term reproducibility and framing the tool's capabilities. I believe MutexaGPT will be a valuable tool for non-computational researchers, making MD-guided protein engineering more accessible. I have no further concerns and recommend accepting the manuscript for publication.

(Remarks on code availability)

Open Access This Peer Review File is licensed under a Creative Commons Attribution 4.0 International License, which permits use, sharing, adaptation, distribution and reproduction in any medium or format, as long as you give appropriate credit to the original author(s) and the source, provide a link to the Creative Commons license, and indicate if changes were made.
In cases where reviewers are anonymous, credit should be given to 'Anonymous Referee' and the source.
The images or other third party material in this Peer Review File are included in the article's Creative Commons license, unless indicated otherwise in a credit line to the material. If material is not included in the article's Creative Commons license and your intended use is not permitted by statutory regulation or exceeds the permitted use, you will need to obtain permission directly from the copyright holder.
To view a copy of this license, visit https://creativecommons.org/licenses/by/4.0/

April 19th, 2026

Kaitlin McCardle, PhD
Senior Editor
Nature Computational Science
Nature portfolio

Dear Dr. McCardle:

Thank you and the reviewers for the constructive comments and evaluation of our manuscript entitled "MutexaGPT: An Intuition-to-Design Translator for Physics-based Enzyme Engineering" (manuscript ID **NATCOMPUTSCI-25-4365**). We have now addressed the comments and requests from the reviewers and submitted our revised version. The original reviewers' comments are provided in full on this letter with a response to each comment labeled "Response" alongside a reproduction of corresponding changes to the text with associated page numbers. The revised manuscript with marked edits is labeled as *mss_marked.pdf*.

I am the submitting author and my contact information is given below. All authors have seen and approved the submission of this manuscript, and it is being submitted exclusively to *Nature Computational Science.* If there are any questions, please do not hesitate to contact me at the e-mail address or phone number listed below.

Sincerely,

Prof. Zhongyue (John) Yang
The SC Family Dean's Faculty Fellow
Department of Chemistry
Data Science Institute
Email: zhongyue.yang@vanderbilt.edu
Phone: (615) 343-9849

Editorial Comments:

1) Please provide experiments/implementation using an open LLM to demonstrate similar/consistent results as the ones using GPT-4o. Please note that this is mandatory for ensuring reproducibility and trustworthiness of the results.

Response: We thank the editor for the comment. We have systematically refactored our entire website backend and added the support for multiple open-weights models (e.g., Kimi K2.5, MiniMax-M2.5, and GLM-5, etc.). Users can now select which model they want to use in MutexaGPT. (Figure R1) We benchmarked all four core agents with an open-weight model (i.e., Kimi K2.5) and added full details (test input, ground truth labels, the testee agent's output, the judge agent's output, the final pass/failure) as spreadsheets in the SI .zip file. The observed performance is similar to the original GPT-4o-based results: QuestionAnalyzer achieves 90% relevance pass and 96% effective completeness pass (previously 97% relevance and 97% completeness), MetricsPlanner achieves 90% pass ratio (previously 91%), MutantPlanner achieved 98% pass ratio (previously 95%), and ResultExplainer achieved 96% overall pass ratio (previously 99%).

We also re-ran the two design campaigns with the open-weight LLM backend (Kimi K2.5) and obtained similarly effective conversation that set up the experiment and similarly informative text summary from about the computed results. Specifically, in the cavity engineering case, the open model planned the same simulation as GPT-4o and prioritized the same set of top 10 mutants as its suggestion. In the cold-adaptation case, the open model also planned the same simulation as GPT-4o and prioritized the same two linker variants with superior domain separation as its suggestion for the experimental test. We included the complete conversation logs and screenshots of final summary pages for these open-weight runs in the SI.

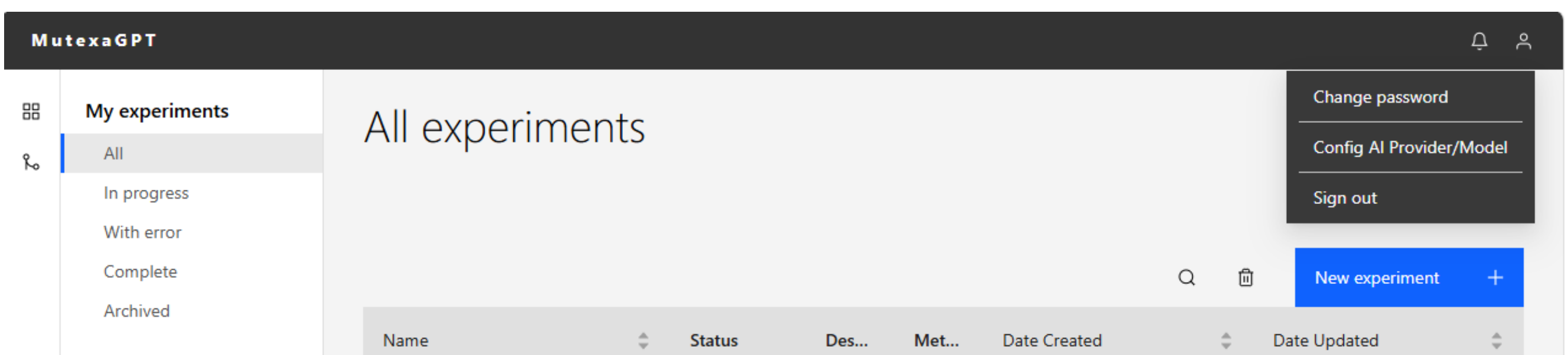

Figure R1. Screenshot of the MutexaGPT interface. Users can switch to open-weights models in the top right corner.

Changes to text:

In Page 34, Section 5a, Computational Method, *Configurations of the large language model (LLM)*, we added

> "Note that users can also choose the LLM model they want to use on the MutexaGPT website. (current options include: GPT-4o, GPT-5.4, Qwen3.6 Plus, MiniMax-M2.5, GLM-5, Kimi K2.5, GLM-4.7, Deepseek v3.2, Gemma 4 26B A4B IT, Gemma 4 31B IT)"

In Supporting Information, we added

> Text S4 "Example dialog between user and MutexaGPT for setting up the cavity engineering study using Kimi K2.5",
>
> Text S5 "Example dialog between user and MutexaGPT for setting up the cold-adaption study using Kim K2.5", and
>
> Text S11 "Example ResultExplainer output and Summary page screenshot for each use case (model = Kimi K2.5)."

In SI.zip file, we added `agent_evaluation` which contains .csv spreadsheets of full performance evaluation details of every agent.

Reviewer #1 (Remarks to the Author):

VANDERBILT UNIVERSITY
COLLEGE OF ARTS AND SCIENCE

Department of Chemistry

Co-reviewed with Reviewer #2
Review Report for manuscript NATCOMPUTSCI-25-4365

The manuscript "MutexaGPT: An Intuition-to-Design Translator for Physics-based Enzyme Engineering" by Shao et al. presents a multi-LLM agent web-based platform for in silico enzyme design/engineering campaigns. The main objective of this work is to enable non-computational experts to execute high-throughput computational simulations that target an intuition-guided initial prompt. To accomplish this, MutexaGPT was designed with three main agents: QuestionAnalyzer, WorkPlanningBoard, and ResultExplainer. The performance of these agents, with particular focus on the QuestionAnalyzer, is assessed using an automated LLM-based benchmarking framework that included curated prompts and classification of results in terms of relevance and completeness to ensure the LLM operates as intended. The authors present two case studies to highlight how MutexaGPT can be used. This work is likely to be high impact with broad interest to the field of enzyme engineering/design for non-computational researchers who would be interested in using the platform, as well as scientists who may be interested in developing LLM agents for their own computational programs. However, we believe there are some revisions required to ensure MutexaGPT is production-ready.

Response: We thank Reviewers 1 and 2 for their thoughtful comments and suggestions. Since their comments are identical and were submitted as part of a co-review, we respond to them together below.

Major Comments:
1. The authors should make it more clear to the non-computational expert what each computable metric could correspond to. For example, (although MutexaGPT does not automate receptor-ligand docking simulations) docking simulations calculate G-scores, not binding affinities, so distinctions like this should be made abundantly clear to the non-computational expert to avoid misinterpretation of results. Line 194 says one goal of the ResultExplainer agent is to "supply just enough domain background so non-experts grasp why a result matters", but no data is included in the manuscript as evidence this happens. The raw ResultExplainer responses for the two case studies should be included in the SI and discussed in the manuscript.

Response: We thank the reviewer for the suggestions. We have improved the ResultExplainer agent together with the improvements suggested by reviewer 1 in comment 3 & 4. For each metric involved, the output of ResultExplainer now contains two complementary paragraphs: "What it may indicate," which explains the physical model underlying the metric and its relevance to the user's original scientific question, and "What it does NOT directly prove," which alerts users to potential overinterpretation or misinterpretation of the results. We have added the raw output of ResultExplainer and screenshots of the Summary page to the Supporting Information (Text S10). In the main text, we updated the discussion about ResultExplainer at the section where it is first introduced.
For the renovated ResultExplainer, we also re-designed its performance evaluation to assess the "interpretation-boundary discipline" (i.e., avoidance of overclaiming beyond the provided data) and discussed it in detail in the revised manuscript and SI.

Changes to text:
In Page 10, Section 2.1, we updated
"Acting under the prompt shown in Supplementary Table S2, Result Explainer handles five top-level goals:

VU Station B#351822
2301 Vanderbilt Place
Nashville, Tennessee 37235-1822
Email zhongyue.yang@vanderbilt.edu
Phone 615.343.9849

(1) translate raw modeling results into concise, domain-relevant takeaways; (2) anchor every explanation to the user's scientific question rather than to the data in isolation; (3) explain, for each reported metric, what it may indicate biologically or structurally and, equally importantly, what it does not directly measure or prove, thereby reducing the risk of over-interpretation by non-computational users; (4) communicate the confidence and limitations of the analysis in light of the available simulation context, such as simulation length and equilibration information; and (5) prioritize the most relevant variants and recommend follow-up experiments, additional simulations, or data checks. (e.g., ResultExplainer can rank/filter enzyme variants based on computed metrics through functional tools; see Text S10 for examples of ResultExplainer output)."

In Supporting Information, we added "Text S10. Example ResultExplainer output and Summary page screenshot for each use case", we added subsections: "*Use case 1: Cavity Engineering*" and "*Use case 2: Cold Adaptation Engineering*".

In Page 15, Section 2.2, we added

"The outputs of ResultExplainer are evaluated by two judge agents, one assessing how well the output aligns to the scientific question from the input and the other assessing interpretation-boundary discipline (i.e., avoidance of overclaiming beyond the provided data), and overall pass requires satisfying both criteria."

In Supporting Information, Page S20, Text S8 "The evaluation system of LLM-agents.", we added

"The detailed evaluation design of all other agents besides QuestionAnalyzer mentioned above are summarized below:

…

ResultExplainer. For ResultExplainer, test inputs are curated by RETestsetCurator as schema-valid JSON payloads containing scientific_question, metrics, results, downloadables, and metadata, with controlled diversity across basic, intent-edge, metric-edge, and extreme-value scenarios. Outputs are evaluated by two specialist judges. RETaskAlignmentJudge scores four binary criteria: (1) whether the explanation directly addresses the stated scientific_question; (2) whether it captures the main decision objective (or explicitly states ranking is not possible, with reason); (3) whether it focuses on metrics relevant to that objective; and (4) whether it provides a decision-oriented takeaway (prioritization or actionable recommendation). It also flags critical_misalignment for substantially off-target responses. REInterpretationBoundaryJudge scores four binary criteria: (1) whether metrics are presented as descriptors/proxies rather than direct proof; (2) whether cautious language is used (e.g., "may indicate," "suggests"); (3) whether the text explicitly states that key metrics do not directly prove activity/affinity/causality; and (4) whether causal overclaims are avoided. It separately flags overclaim_detected when major overclaim appears. A case is counted as overall pass only when both judges pass (alignment pass and interpretation-boundary pass). Same as other agents, each test case is executed in three independent replicas."

2. We really appreciate the effort behind assessing the QuestionAnalyzer LLM agent performance. For this work to be of value to the community, it is essential that it operates as intended. However, the current information regarding the performance evaluation is not enough for us to determine if MutexaGPT operates as intended, for a few reasons:

Response: We thank the reviewer for recognizing our efforts in the performance evaluation of the agents. These comments are helpful and constructive. We improved the performance evaluation systematically and added detailed information about the evaluation in both SI and main text. The point-to-point response is provided below.

2.1. The authors explain that a set of test inquiries were generated (by an LLM) and assigned attributes/labels corresponding to their relevance and completeness. The SI Figure S2 indicates the TestSetCurator may have included some "human correction" to generate the test questions and corresponding labels, whereas in the main text it says the labels were "compared with manually verified ground-truth labels" (line 216, among other

places). The authors should include this entire test set in the SI, along with their labels, and performance assessment of each question with the different MutexaGPT agents (and corresponding prompt variations). This transparency is mandatory to ensure the evaluation spans an appropriate domain of applicability such that MutexaGPT operates as intended.

Response: We thank the reviewer for the comment. We have included the entire test set for every agent in SI.zip as .csv files. Each line in these files records a test case with its corresponding test input, ground truth labels, the testee agent's output, the judge agent's output, the final pass/failure.

Changes to text:
In Page 15, Section 2.2, we added

> "See SI.zip for spreadsheets that contain the test input, ground truth label, agent's output, the judge agent's output, and the pass/failure of every test case of each agent in MutexaGPT."

In SI.zip, we added `agent_evaluation` which contains full performance evaluation details of every agent.

2.2. Only 20/120 inquiries were "irrelevant", and the Full QuestionAnalyzer prompt design performance for relevance was 96.7%. Our understanding is that corresponds to 116 inquiries were correctly identified as either relevant or irrelevant, but details beyond that are lacking. Since only ~17% of the test data was "irrelevant", the design of this evaluation is skewed, especially if the prompt design is more likely to consider an irrelevant inquiry as relevant (rather than more likely to consider a relevant inquiry as irrelevant). This test should be designed with less bias so it can be more accurately assessed. Furthermore, presentation of the performance results for each inquiry will help us understand if the prompt design has any bias. We believe this is very important because if MutexaGPT is not able to detect an irrelevant inquiry accurately, this could potentially lead to wasted computational time (and thus wasted resources).

Response: We thank the reviewer for the suggestion. We added 80 irrelevant test cases in the test of QuestionAnalyzer as suggested by the reviewer to balance the evaluation (now 100/200 are relevant and 100/200 are irrelevant). After balancing, the relevance performance remains consistent with our original conclusion: from three independent runs, the QuestionAnalyzer with "Full prompt" achieves 97.0% relevance accuracy. We updated Figure 2B with the numbers from the balanced test.

Changes to text: In Page 9, Section 2, we updated Figure 2B.
In Page 12, Section 2.2, we updated "The performance was evaluated against six different prompt designs (Figure 2B) on the same curated test set of 200 inquiries (100 irrelevant and 100 relevant, …"

2.3. The performance percentages in Figure 2B do not make sense. If there are 120 test inquiries and no possibility for "partial" pass, then the percentages of 99.4, and 97.2 are not possible. Maybe our interpretation of how this performance is calculated is wrong. If not, the authors should carefully review all performance data.

Response: We thank the reviewer for this careful check. We confirm that the reviewer was correct: the percentages in the previous Figure 2B were miscalculated. We had incorrectly averaged pass rates across three independent runs, which produced the invalid percentages values (e.g., 99.4%, 97.2%). In the revised manuscript, we corrected the calculation to be consistent with the description in the main text: a prompt design is counted as failing for an inquiry

VU Station B#351822
2301 Vanderbilt Place
Nashville, Tennessee 37235-1822
Email zhongyue.yang@vanderbilt.edu
Phone 615.343.9849

if any of the three runs fails that test. We then recomputed all Figure 2B metrics accordingly and re-checked the full table. We have updated Figure 2B and all relevant numbers in the main text.

After correction, in the relevance test, all pass rates remain high. In the completeness test, the strict-pass rates decrease overall, double-check rates increase, and effective-pass rates remain similar. Importantly, the relative ranking across prompt designs and the qualitative conclusions are unchanged. The corrected analysis further highlights that ICL and CoT are important, as the No-ICL and No-CoT prompt variants now show larger drops compared with the Full prompt.

Changes to text: In Page 9, Section 2, we updated Figure 2B.
In Page 12, Section 2.2, we updated all numbers in the paragraph:

> "The benchmark results show that the *Full* prompt (see Table S2), … without them, the model defaults to hesitation and repetitive questioning instead of reliable information gathering."

`2.4. The authors should include a discussion about why certain inquiries failed at certain points.`

Response: We thank the reviewer for the suggestions. In the revised manuscript, we now explicitly discuss why some inquiries fail at specific points in the QuestionAnalyzer performance evaluation (with "Full" prompt design, n = 200, three independent runs). For the relevance test, there are 6 failed inquiries under a strict evaluation rubric (an inquiry is counted as failure if any single run fails), and all 6 are false negatives where relevant inquiries are incorrectly rejected. These failures all come from broadly phrased or extremely underspecified questions (e.g., "What effect do ligands have on proteins?"; "How do genetic alterations affect the behavior of the protein in the cell?"). Across runs, this behavior is partially consistent: 2 of the 6 failed relevance inquiries recur in all three runs, while the others appear in only one or two runs.

In the completeness test, focusing on effective-pass failures (as the double-check scenario is already discussed in the original manuscript), only 3 inquiries fail under strict a strict evaluation rubric. These failures are not fully consistent across runs: none fails in all three; one fails in two runs. A closer look into these cases suggests that they are marginal failures. Specifically, the input use "flexibility of the loop region (defined by residues 50-60)" to describe the property of interest, the label marked "property" as incomplete likely due to the vagueness of the word "flexibility". In three independent runs, two picked up the word "flexibility" correctly and asked for clarification from the user, while the failed run think the current description is already complete (i.e., a false positive). However, we should note that "flexibility of the loop region (defined by residues 50-60)" is marginally an incomplete description because in the MutexaGPT workflow, the following MetricsPlanner is capable of mapping it to metrics such as RMSD, etc.

Changes to text: In Page 13, Section 2.2, we added "Error analysis of the result of the *Full* QuestionAnalyzer shows that relevance failures are exclusively false negatives, primarily arising from overly vague inquiry (e.g., "What effect do ligands have on proteins?"), while effective-pass completeness failures are rare (3/100) and mostly associated with marginally ambiguous property definitions (e.g.: "flexibility of …") rather than systematic inability to identify incomplete inquires."

`2.5. Line 226: Please elaborate on what is meant by "behave correctly". Were the missing pieces of information in under-specified explicitly labeled, and assessed if that corresponded to the QuestionAnalyzer request? How was correctness determined?`

Response: We thank the reviewer for the comment. In our evaluation, correctness was defined based on predefined metrics for the completeness test. For each practical inquiry, the test set provides an explicit reference annotation of the missing information, using three predefined category labels: protein, mutation, and property. The QuestionAnalyzer output is then parsed by an independent judge model, which returns the set of missing categories the agent requested from the user. A strict completeness pass is assigned only when the predicted set exactly matches

the reference set (set(predicted) == set(reference)), i.e., precisely identifying all the required missing pieces. We additionally report an effective-pass metric, where a case passes if all gold missing items are covered even if extra clarification requests are included (set(reference) ⊆ set(predicted)), and this difference is reported as the "double-check" component. Across three independent runs, the revised analysis uses a strict evaluation rubric (an inquiry is counted as failed if any run fails), so "all three runs behave correctly" means the criterion above is satisfied in each run. We added clarification in the revised manuscript to reduce vagueness.

Changes to text: In Page 12, Section 2.2, we added "i.e., the agent asks for exactly the information categories that are actually missing in the inquiry: protein, mutation, and/or property; …"

2.6. More information regarding the performance of other agents is warranted (in addition to providing the specific inputs used to assess their performance and definition of fails/passes). In the paper, only the performance of QuestionAnalyzer is discussed in detail, whereas that of the others is just mentioned that it was completed. For example, the agent MetricsPlanner has a success rate of 91%. What goes wrong for it to fail 9% of the time? This one seems very crucial to get correct. Furthermore, MutationPlanner has a 100% success rate. Is this because in all the test cases the exact mutations were provided?

Response: We thank the reviewer for the suggestions. In the revised manuscript and SI, we now provide detailed benchmark designs, pass/fail definitions, and representative failure modes for all other agents. For the reviewer's question about the MetricsPlanner, the reported 91% means 9/100 test cases fail under a strict exact-match criterion (the recommended metric and MD-model pair must include the curated ground truth label). Most failures are near-miss planning errors, where the agent proposes plausible but non-labeled alternatives or a mismatched MD model for the labeled metric.

For the reviewer's question about the MutantPlanner, the 100% score is not because all test cases provide exact mutations; the benchmark includes unrestricted, pocket/interface/distal, and combinatorial mutation requests in natural language, and only a subset are explicit site-level mutations. Note that we found a bug in the MutantPlanner test that overestimated the pass-rate. After fixing the bug, the pass-rate goes to 92% (4/50 cases failed; a case is count as pass only when all three independent runs pass). We have added these clarifications in the main text and directed readers to SI sections with full details (benchmark designs, pass/fail definitions, and representative failure modes).

Changes to text:

In Page 14, Section 2.2, we added

> "For MetricsPlanner, the test-set curator generates inputs (that matches the format of the QuestionAnalyzer's final output; 100 cases asking about properties that can map to one of our supported metrics, and 100 cases asking about properties that are not) from ground-truth metric/MD-model labels, and a case passes only when the agent's recommended candidates contain the labeled metric-model pair or inform user when none of the supported metrics can correspond to the inquiry. For MutantPlanner, we used a set of manually curated inputs. It contains a balanced set of unrestricted, pocket, protein-protein interface, distal (relative to the pocket) mutational libraries, and designated site mutations (10 for each, 50 in total). The correctness is judged by whether the generated EnzyHTP mutation pattern is semantically equivalent to the labeled library (i.e., they are parsed by EnzyHTP and directly compared for equivalence). For ResultExplainer, a set of JSON payloads are curated by a curator agent (containing "scientific question", "metrics", "results", "downloadables", and "metadata"; 100 in total). The outputs of ResultExplainer are evaluated by two judge agents, one assessing how well the output aligns to the scientific question from the input and the other assessing interpretation-boundary discipline (i.e., avoidance of overclaiming beyond the provided data), and overall pass requires satisfying both criteria. Full benchmark inputs, pass definitions, and per-run outputs are provided in Text S8 and SI.zip/agent_evaluation.

VU Station B#351822
2301 Vanderbilt Place
Nashville, Tennessee 37235-1822
Email zhongyue.yang@vanderbilt.edu
Phone 615.343.9849

The results show that the prompt design for each agent is highly effective in their respective roles: for WorkPlanningBoard, MetricsPlanner on average passes 91% of its test cases, MutantPlanner achieves a 95% success rate; ResultExplainer met all evaluation criteria in 99% of the test cases. While not directly comparable, these performance levels are strong in the broader context of LLM benchmarks for scientific tasks (for reference, recent scientific-discovery benchmarks[65] typically report only 0.60–0.75 question-level accuracy across domains, while the best model in a question-clarification benchmark[66] reaches a maximum success rate of 60.1%). See Text S9 for error analysis of each agent. See SI.zip for spreadsheets that contain the test input, ground truth label, agent's output, the judge agent's output, and the pass/failure of every test case of each agent in MutexaGPT."

In Supporting Information, Page S20, Text S8 "The evaluation system of LLM-agents.", we added three sections under the existing example of QuestionAnalyzer, for each other agents:

"The detailed evaluation design of all other agents besides QuestionAnalyzer mentioned above are summarized below:

MetricsPlanner. To evaluate MetricsPlanner, we use a …

MutantPlanner. MutantPlanner is evaluated with ...

ResultExplainer. For ResultExplainer, test inputs are curated … "

In Supporting Information, Page S20, we added Text S9 "Failure mode analysis in the agent performance evaluation".

```
2.7. What is considered "failed" in MetricsPlanner? Is each EnzyHTP computable metric represented in the test inquiries? To accomplish the main objective, it is paramount that MutexaGPT translates the intuition into an appropriate calculatable property. Therefore, this performance must be critically assessed.
```

Response: We thank the reviewer for the comment. In our benchmark, a MetricsPlanner case is counted as failed under a strict evaluation rubric: for cases where MutexaGPT can provide a supported metric (e.g., cavity size, RMSD, and so on), failure means that the agent output does not include the curated ground truth metric(s); for cases where MutexaGPT has no supported metrics, failure means the agent does not return a proper unsupported notice and instead proceeds with recommendation/workflow steps. We have now clarified this pass/fail definition in the revised manuscript (Section 2.2) and SI (Text S8). We also confirm that all EnzyHTP-supported MetricsPlanner metrics are represented in the supported test inquiries, and we additionally include a dedicated unsupported-property set (same number of cases as the supported test cases) to assess boundary behavior. Full per-case inputs/labels/outputs are provided in SI.zip for transparent critical assessment.

Changes to text:

In Page 14, Section 2.2, we added

"For MetricsPlanner, the test-set curator generates inputs (that matches the format of the QuestionAnalyzer's final output; 100 cases asking about properties that can map to one of our supported metrics, and 100 cases asking about properties that are not) from ground truth metric/MD-model labels, and a case passes only when the agent's recommended candidates contain the labeled metric-model pair or inform user when none of the supported metrics can correspond to the inquiry. … Full benchmark inputs, pass definitions, and per-run outputs are provided in Text S8 and SI.zip/agent_evaluation."

In Supporting Information, Page 20, Text S8 "The evaluation system of LLM-agents.", we added

"The detailed evaluation design of all other agents besides QuestionAnalyzer mentioned above are summarized below:

MetricsPlanner. To evaluate MetricsPlanner, we use a …"

```
3. The results in the MutexaGPT platform examples do not show any statistics, even though replicate simulations were performed. What was the length of equilibration for the MD simulations performed? What analysis was used to confirm that the system has equilibrated? This type of information is always necessary to include when discussing simulation results, so it would be
```

valuable to display this information in the "executive summary" section of the MutexaGPT web platform, and included in the ResultExplainer agent.

Response: We thank the reviewer for the suggestions. We have now improved ResultExplainer to have it discuss about the equilibration statistics in the "executive summary" based on Chodera automated equilibration detection scheme.[1] We have also improved the front and back end of the website to plot temperature, energy, and pressure during the equilibration steps and display in the Summary page so that users can also check by themselves (See example output and screenshot for the use cases in Text S10).

Specifically, for the length of the equilibration, we wrote, in Section 5a, Computational Method, *Molecular dynamics*: "… 3) 500 ps equilibration at 300 K and 1 atm with the same backbone restraint. 4) 500 ps equilibration at 300K and 1 atm without the backbone restraint. …" These two steps combine into 1 ns equilibration. This information is then passed to ResultExplainer in the "metadata" field of its payload and reported to users in the Executive Summary.

For the equilibration analysis, we implemented the Chodera automated equilibration detection scheme in the backend which detects the equilibration onset (t0) by scanning each trajectory series to maximize effective sample size (Neff) with statistical inefficiency correction, then classifies each series as equilibrated only when the post-t0 tail has >10 points (i.e., 0.05 ns; applied to temperature, pressure, and total energy; overall status is equilibrated only if all assessed series pass). The resulting equilibration statistics (including equilibration assessment method, number of equilibrated runs, and per-run assessment summary) are now passed to ResultExplainer through metadata and reported to users in the Executive Summary (Figure S3).

Changes to text:

In Page 33, Section 5a, Computational Method, subsection *Molecular Dynamics*, we added

> "Chodera automated equilibration detection scheme[97] is used to analyze the equilibration statistics in the backend and the results are reported by ResultExplainer in the final Summary page."

In Supporting Information, we added Text S10, titled "Example ResultExplainer output and Summary page screenshot for each use case".

4. The reported "hit rate" for the cavity size example appears cherry-picked (e.g., top 10 variants). Rank-based metrics (e.g., Spearman's correlation across all variants, or at least the 66 experimental "ground-truth" hits) would be more appropriate. We understand the objective of this paper is not to optimize these two enzymes, therefore the success of these campaigns is not critical to this manuscript. Rather, these examples showcase what MutexaGPT could help automate. In line with this, these results sections should include more specifics on what the MutexaGPT outputs were, such as including a screen shot of the MutexaGPT ResultsExplainer output in the SI. Line 325 says "MutexaGPT's top-ranked mutations", which is a little misleading, since those are the top ranks from the simulation data (MutexaGPT did not provide any inference; it is just displaying the simulation results in natural-language, to our understanding). Limitations, especially those that a non-computational expert would be unaware of, should be discussed in these example sections and explained by the ResultsExplainer (e.g., cavity size as a descriptor, force-field choice, simulation length, solvent model, etc.). It would also be useful to discuss whether ResultExplainer could suggest methodological improvements, such as performing longer simulations on top-ranked variants.

Response: We thank the reviewer for the suggestions. In the revised manuscript, we clarified the final output of MutexaGPT in the main text where the two use cases are discussed. It says

VU Station B#351822
2301 Vanderbilt Place
Nashville, Tennessee 37235-1822
Email zhongyue.yang@vanderbilt.edu
Phone 615.343.9849

> "Eventually, MutexaGPT addressed the user's initial request, "*Help me find mutations that make the active site larger*", by ranking all 600 variants according to their ensemble-averaged cavity volumes and identifying the 10 candidates showing the greatest increases in volume (Figure 3D, see screenshot of the Summary page in Figure S3)"

In the backend, ResultExplainer calls function tools to filter and rank enzyme variants based on the calculated metrics. We also added screenshots of the final MutexaGPT Summary page that contains the output of ResultExplainer in the SI.

To make the readers more informed about the predictive power of the metrics, we added a cumulative hit curve in the revised SI, which shows that enrichment of beneficial mutants is strongest in early ranks and declines in later ranks, supporting the practical value of MutexaGPT for experimentally screening beneficial mutants. We did not add Spearman correlation as a primary metric for this use case because the intended application is to identify the most promising beneficial variants for experimental testing, rather than to predict the global ranking across both the beneficial and deleterious mutations. This is because the global rank-correlations are often dominated by the large size of medium-to-low activity/noisy regime and significantly underrepresent practical top-hit retrieval. Specifically, for the cavity engineering case, the overall Spearman correlation for the 66 experimentally characterized mutants (between ensemble average cavity volume and the activity of the largest substrate) is only 0.01. However, if we take the top 1/4 most active mutants from the 66 experimental hits, the Spearman correlation will rise up to 0.67. Note that many mutants only have <10-fold activity improvements, while the top hits can have >200-fold activity improvements.

The reviewer made a great point about including the limitations in the output of ResultExplainer. We have improved ResultsExplainer to include explicit discussion about simulation caveats and context for non-computational users (e.g., descriptor limitations, force field, simulation length, solvent model, and single-replica constraints) and to suggest methodological follow-ups such as longer and/or replicated simulations for top-ranked variants. Additionally, ResultExplainer will now also discuss how user should interpret the ranking from the metrics, what it is recommended to be used for and what it is not. For example, for metrics such as cavity volume, it will say things like "Good for prioritizing variants by predicted cavity expansion but should not be used to fully account for the mechanism or in a correlation study…"

Changes to text:

In Page 10, Section 2.1, we revised

> "… prioritize the most relevant variants and recommend follow-up experiments, additional simulations, or data checks. (e.g., ResultExplainer can rank/filter enzyme variants based on computed metrics through functional tools; see Text S10 for examples of ResultExplainer output)."

In Page 18, Section 3.1, we revised

> "Eventually, MutexaGPT addressed the user's initial request, "*Help me find mutations that make the active site larger*", by ranking all 600 variants according to their ensemble-averaged cavity volumes and identifying the 10 candidates showing the greatest increases in volume (Figure 3D, see screenshot of the Summary page in Figure S3)"

In Supporting Information, we added Figure S9 "The cumulative hit curve for use case 1: Cavity Engineering."

In Supporting Information, Page 22, "Text S10. Example ResultExplainer output and Summary page screenshot for each use case", we added subsections: "*Use case 1: Cavity Engineering*" and "*Use case 2: Cold Adaptation Engineering*".

5. It was not discussed what the workflow would look like if the intuition does not correspond to an EnzyHTP computable metric, which we believe is important to address. Would this count as "irrelevant" and be accurately flagged by MutexaGPT? Are there examples of these scenarios in the performance benchmarking? Overall, the limitations of MutexaGPT (and inherently, the limitations of EnzyHTP), should be identified in the discussion. Paragraph starting at line 506 would be appropriate to clarify this.

Response: We thank the reviewer for the comment. We agree that unsupported metrics for the user intuitions should be explicitly evaluated and discussed as a key limitation of both MutexaGPT and the current EnzyHTP metric space. In the revised evaluation, we added a dedicated set of 100 unsupported metrics test cases for MetricsPlanner (examples include *expression yield*, *immunogenicity*, and *folding kinetics*). For these cases, the expected behavior is to flag the request as out of scope (i.e., not currently computable by EnzyHTP) rather than forcing an invalid metric mapping. A typical output is: *"Your target property ... is not currently supported by our service. Feel free to contact the developer to request a new feature."* On this unsupported set, MetricsPlanner achieves a pass ratio of 91.0%. Combined with the original 100 supported-intuition cases, the overall pass ratio is 91% (200 total cases). A deeper look into the failed cases shows that errors are concentrated in marginally supported properties (e.g., "loss of function rate", or "shelf stability"), where the boundary between supported and unsupported intuition is ambiguous. In these cases, MetricsPlanner tends to decompose the requested intuition into lower-level properties (e.g., enzyme activity/thermostability, activation barrier, and substrate binding) and then map them to available EnzyHTP metrics instead of returning a strict unsupported-property notice.

We have added explicit discussion of the limitation of metric support in the Discussion section we added a new SI table that lists metrics MutexaGPT currently supported.

Changes to text:
In Page 29, Section 4, we added

> "How does MutexaGPT work beyond the two case studies? Currently, MutexaGPT supports high-throughput calculations of a list of function-related metrics: … Note that a practical limitation of MutexaGPT also comes from this list of computable metrics. If a property of interest from the user cannot be mapped to a currently supported EnzyHTP-computable metric, MutexaGPT cannot produce a physically grounded simulation answer and should instead return an explicit unsupported-property notice. Nevertheless, MutexaGPT is designed to be extensible, where new metrics can be easily (by changing just two files if they are supported by EnzyHTP) added in the future to support more enzyme engineering tasks (One can refer to Table S1 for currently supported metrics; We should note that the underlying EnzyHTP engine is developed with interface modules to common software such as Amber for trajectory analysis, Gaussian/xTB for quantum mechanics, Multiwfn for wavefunction analysis, Rosetta for biophysics, etc. and thus inherits the metrics-support-boundary of these software)."

In Supporting Information, we added Table S1 "Metrics MutexaGPT currently supports".

```
Minor Comments:
6. If a non-computational researcher used MutexaGPT and then wanted to publish a manuscript, will the output provided by MutexaGPT be able to supply reproducible methods? This is already a concern in the field. Some discussion on this should be included, because non-computational experts cannot be expected to know what level of detail is required for simulation method reproducibility.
```

Response: We thank the reviewer for the suggestions. We added a new feature to the MutexaGPT web interface that allows users to export the exact EnzyHTP script used to run the simulation and analysis. This export captures the full executable workflow and parameters. This way users can share a fully reproducible computational protocol directly. We also added corresponding discussion in the revised manuscript.
Changes to text: In Page 8, Section 2.1, we added

> "Note that to further support reproducibility if users want to publish any data generated from MutexaGPT, in the workflow review page, the website allows users to export the exact EnzyHTP Python script that is executed. This script contains full simulation setup and parameters and can be directly rerun or cited in Methods/Supplementary sections to ensure reproducibility, especially for users without extensive computational background."

7. In the introduction and discussion, it should be made clearer that MutexaGPT is an automation tool, not a predictive tool. That is, it translates the "intuition" enclosed in the user's question to a metric (or multiple metrics) that can be computed from simulation, but the available metrics are hard coded into the system (EnzyHTP capabilities as well as the MetricPlanning LLM Agent prompt).

Response: We thank the reviewer for the suggestions. We agree that MutexaGPT should be described as an automation and translation tool rather than a predictive tool. In the revised Introduction and Discussion, we now clarify this scope explicitly.

Changes to text:
In Page 5, Section 1, Introduction, we added

> "Note that although MutexaGPT ranks and filters enzyme variants based on computed metrics, it is not a predictive AI tool, but instead an integrated system for automation and intuition-translation."

In Page 28, Section 4, Discussion, we added

> "Notably, while MutexaGPT ranks and filters enzyme variants based on computed metrics, it is not a predictive AI tool, but instead an integrated system for automation and intuition-translation."

8. Section 2.2 was challenging to follow. Some of the content in the SI should be moved to the main text for better clarity. An introduction into the fact that LLM agents need to be given a prompt would be beneficial to the reader to make it clear that this section assesses the performance based on how the agents were designed (rather than performance of an enzyme design study, for example). Furthermore, it should be made very clear that the "prompt design" is the prompt given to the agent, and not the prompt that is provided by the user (aka the inquiry/question/input). Explanation of all different designs (CoT scaffold, ICL, among others) should be moved to the main text.

Response: We thank the reviewer for the suggestions. We have revised Section 2.2 based on the reviewer's suggestions.

Changes to text:
In Page 11, Section 2.2, we added

> "Note that, an LLM agent in MutexaGPT is a large language model (e.g.: GPT-4o[58], etc.) configured with a system prompt that instruct and regulate its behavior. In this section, "prompt design" refers specifically to the design of the system prompt that governs the agent's behavior, rather than the natural-language inquiry/input provided by the user. We therefore benchmark how different prompt-engineering choices affect agent performance under the same task definition and curated test set."

In Page 12, Section 2.2, we revised

> "The benchmark results show that the Full prompt (see Table S2), which contains chain-of-thought[59-61] (CoT; i.e., the system prompt contains a list of instructions directing the agent to follow a logic chainCoT) scaffold and seven carefully selected in-context learning[62] examples (ICL; i.e., examples, as input and output pairs, are provided in the system prompt, also known as few-shot learning), emerges as the best performing design."

9. Will the LLM retain information provided by the user? Is there any potential issue with data protection?

Response: We thank the reviewer for the comment. The LLM will not retain information provided by the user. The conversation between the user and LLM will be deleted by the backend of MutexaGPT (using the

`client.conversations.delete` endpoint). Note that the conversation messages between the user and LLM will be stored in our own database so that the user can review their conversation history on our website. Our privacy policy is listed at https://enzyhtp.app.vanderbilt.edu/docs.html?doc=privacy.

`10. The purpose of the survey is unclear and does not seem directly relevant to the main objective of the paper. Consider moving all survey-related content to the SI. Perhaps the authors could estimate how long it took the user to interact with MutexaGPT in the two examples and compare that time with how much time it takes for the average person to set up an MD simulation.`

Response: We thank the reviewer for the suggestions. We have removed all survey-related content as suggested by other reviewers.

Changes to text:
In Section 1, we removed the sentences about the survey.
In Section 1, we removed Figure 1A.
In Supporting Information, we removed Figure S3.

`11. Line 256: "second" was already used. Should be "third"`

Response: We thank the reviewer for the comment. We fixed the wording accordingly.
Changes to text: In Page 14, Section 2.2, we changed "Second" to "Third".

`12. Regarding section 2.2: Is the performance good in the general LLM context? Are there any references to more LLM specific research to suggest this is a good performance? Include discussion on potential pitfalls of using the full prompt (i.e., could this cause the agent to be restricted?)`

Response: We thank the reviewer for the comment. While our task-specific performance numbers are not directly comparable with other LLM benchmarks, the level of performance is considered to be higher than what is typical in LLM benchmarks for scientific tasks. For example, recent scientific-discovery benchmarks[2] typically report only 0.60–0.75 question-level accuracy across domains, while the best model in a question-clarification benchmark reaches a maximum success rate of 60.1%.[3]

We have added discussions about the performance level in general LLM context and potential pitfalls of using the full prompt in the revised manuscript.

Changes to text:
In Page 15, Section 2.2, we added

> "While not directly comparable, these performance levels are strong in the broader context of LLM benchmarks for scientific tasks (for reference, recent scientific-discovery benchmarks[65] typically report only 0.60–0.75 question-level accuracy across domains, while the best model in a question-clarification benchmark[66] reaches a maximum success rate of 60.1%)."

In Page 14, Section 2.2, we added

> "Note that, while the Full prompt design performs better in our context, a richer system prompt carries a potential downside: it improves in-domain precision by constraining the agent, but also reduces flexibility on atypical or out-of-distribution requests by anchoring behavior to the included examples and interaction schema.[63, 66]"

`13. Line 335: "moderate MD length" is a very domain specific and ambiguous term. Does MutexaGPT know if a simulation length is short vs moderate? Moreover,`

VU Station B#351822
2301 Vanderbilt Place
Nashville, Tennessee 37235-1822
Email zhongyue.yang@vanderbilt.edu
Phone 615.343.9849

in line 575, it is mentioned that "MD simulation time was set to 50 ns". Is this information that the non-expert user is expected to provide, or is there a default value? And are there any fail-safes to the simulations? If it blows up, how is it communicated to the non-expert? Examples of this would be very valuable.

Response: We thank the reviewer for the comment. We have revised this sentence to make it more concrete about the point we want to express: in our investigation about how the length of the MD simulation affect the outcome of use case 1, we tested 10 different values, the results shows simulations of 15 ns or longer consistently identify at least 4 hits.

For the question "Does MutexaGPT know if a simulation length is short vs moderate?", in our experience working with MutexaGPT, specifically, the ResultExplainer, it can tell when a simulation is too short (i.e., when we test the system using 1 ns MD, ResultExplainer always complains about it) or moderate (e.g., 50 ns).
The length of the MD simulation in MutexaGPT is default to 50 ns. However, in the workflow review page, users can configure any length they want by entering a number.

If a simulation blows up, the backend will raise an error as it will cause an illegal memory access on the Amber end. The user will receive a notice saying the experiment failed with the error message from the backend.

Changes to text:
In Page 18, Section 3.1, we revised "… these results show that moderate-length (i.e., 15 ns or longer) MD simulations …"

14. Regarding the discussion paragraph beginning at line 524, we believe MutexaGPT should be classified at the assistant-level currently, particularly given that the MetricPlanner LLM agent prompt clearly guides what metric each "intuition" corresponds to. The evaluations that were performed do not indicate that a collaborator level has been reached, but the authors could consider different evaluations to assess this more quantitatively and thus strengthen their argument that MutexaGPT is beyond the assistant level.

Response: We thank the reviewer for the comment. We also agree that MutexaGPT remains an assistant-level system. We have clarified this point in the revised manuscript.
Changes to text: In Page 31, Section 4, we revised "For protein engineers with little-to-no experience in computational modeling, MutexaGPT, while remaining an assistant-level system, takes a step toward becoming a collaborator-level agent for enzyme engineering."

VANDERBILT UNIVERSITY
COLLEGE OF ARTS AND SCIENCE
Department of Chemistry

Reviewer #3 (Remarks to the Author):

The authors describe an agentic framework "MutexaGPT" for LLM-powered text-based automation of their previously developed automated simulation framework "EnzyHTP." This links physical intuition to high-throughput simulation and analysis. The system is intended for users with expertise in enzyme engineering, but little to no molecular modeling expertise. Two specific and straightforward enzyme designs are demonstrated, including experimental validation. Strong claims regarding utility are made that are not well supported by the data. As currently written, there are several major deficiencies in the evaluation and reporting of results, including:

Response: We thank the reviewer for the suggestions. The point-to-point response is provided below.

Major:

1. The authors present a survey with no methodological details, (ie. number of subjects, response rates) and the manuscript lacks a formal statement regarding ethical oversight (ie. consent / anonymization). Also, a median of 2 days is reported though it is not provided as a discrete option within the survey.

Response: We thank the reviewer for the suggestions. We agree that the survey is not representative. We have removed all survey-related content as suggested by you and other reviewers.

Changes to text:
In Section 1, we removed the sentences about the survey.
In Section 1, we removed Figure 1A.
In Supporting Information, we removed Figure S3.

2. The utility for enzyme design is overstated in the abstract and introduction. The capacities of MutexaGPT carry at least the limitations of EnzyHTP. The statement "hypothesis refinement is still limited to correcting non-scientific statements, retrieving missing information for a vague request, and mapping general properties of interest to computable metrics" (page 27) clearly defines the scope of the contribution and should be emphasized earlier on, while more exaggerated claims should be tempered.

Response: We thank the reviewer for the suggestions. We agree that our previous wording in the Abstract and Introduction could overstate the current utility of MutexaGPT for enzyme design. In the revised manuscript, we have tempered these claims and explicitly moved the scope statement earlier in the paper.

Changes to text:
In Page 1, Abstract, we revised

> "Here we introduce MutexaGPT, an open-access, multi-agent large language model (LLM) platform that translates enzyme engineering intuition to physics-based simulations and thus variant designs."

In Page 5, Section 1, Introduction, we added

> "Note that although MutexaGPT ranks and filters enzyme variants based on computed metrics, it is not a predictive AI tool, but instead an integrated system for automation and intuition-translation."

In Page 6, Section 1, Introduction, we revised

VU Station B#351822
2301 Vanderbilt Place
Nashville, Tennessee 37235-1822
Email zhongyue.yang@vanderbilt.edu
Phone 615.343.9849

> "Complementary to screening-dominated approaches, MutexaGPT provides a scalable route to operationalize intuition-driven, physics-guided enzyme engineering within the scope of currently implemented metrics and simulations."

`3. The design capacity is linked to the capacity for (high throughput) MD observables to be directly translated to enzyme design. This is only straightforward for a number of use cases such as increasing volume or distance. It is unclear how the agent will respond or what the hit-rate will be in examples where there is not a clear correlation between MD variable and desired activity change.`

Response: We thank the reviewer for the comment. To address this comment, we revised the performance evaluation. In the revised evaluation, we added a set of 100 test cases for MetricsPlanner, where MutexaGPT has no supported molecular metrics for the user-desired properties (examples include asking about *expression yield*, *immunogenicity*, *folding kinetics*, and so on). For these cases, the expected behavior is to flag the request as being out of scope (i.e., not currently computable by EnzyHTP) rather than forcing an invalid metric mapping. A typical output is: *"Your target property ... is not currently supported by our service. Feel free to contact the developer to request a new feature."* In this unsupported set, MetricsPlanner achieves a pass ratio of 91.0%. Combined with the original 100 supported-inquiry cases, the overall pass ratio is 91% (200 total cases). A deeper look into the failed cases shows that errors are concentrated in marginally supported properties (e.g., "loss of function rate", or "shelf stability"), where the boundary between supported and unsupported intuition is ambiguous. In these cases, MetricsPlanner tends to decompose the requested intuition into lower-level properties (e.g., enzyme activity/thermostability, activation barrier, and substrate binding) and then map them to available EnzyHTP metrics instead of returning a strict unsupported-property notice. We have added explicit discussion of the metrics-support-related limitation in the Discussion section and we added a new SI table that lists metrics MutexaGPT currently supported.

Changes to text:

In Page 29, Section 4, we added

> "How does MutexaGPT work beyond the two case studies? Currently, MutexaGPT supports high-throughput calculations of a list of function-related metrics: … Note that a practical limitation of MutexaGPT also comes from this list of computable metrics. If a property of interest from the user cannot be mapped to a currently supported EnzyHTP-computable metric, MutexaGPT cannot produce a physically grounded simulation answer and should instead return an explicit unsupported-property notice. Nevertheless, MutexaGPT is designed to be extensible, where new metrics can be easily (by changing just two files if they are supported by EnzyHTP) added in the future to support more enzyme engineering tasks (One can refer to Table S1 for currently supported metrics; We should note that the underlying EnzyHTP engine is developed with interface modules to common software such as Amber for trajectory analysis, Gaussian/xTB for quantum mechanics, Multiwfn for wavefunction analysis, Rosetta for biophysics, etc. and thus inherits the metrics-support-boundary of these software)."

In Supporting Information, we added Table S1 "Metrics MutexaGPT currently supports".

`4. Use is intended for non-molecular modeling experts. However, 30 us of simulation requires substantial computational resources. It is unclear whether the web-app has the capacity to host these or whether additional resources are needed. If this app is capable of supporting, are the benchmarks for the time it will take to run the simulations?`

Response: We thank the reviewer for the suggestions. The web app executes simulations through Vanderbilt's Advanced Computing Center for Research and Education (ACCRE; approximately 300 GPUs and 20,000 CPU

cores), which supports the workloads in this study. At the same time, because this is a shared institutional resource, we hope to avoid overburdening it. So, we provide a “run on your own” option for larger campaigns, as written in the manuscript:

> “The platform also provides a choice for users to download a folder of prepared input files of all needed simulations and a simple script for configuring the environment, executing them using the users’ local computing resources, and uploading them back to MutexaGPT if needed.”

In addition, we plan to subscribe more computing resources from cloud providers (e.g., Amazon Web Services) to adapt to potential heavier workloads.

5. The performance reported in figure 2B is difficult to interpret without knowledge of how challenging or diverse the discrimination tasks were. It is also not clear how these evaluation metrics compare to other state-of-the-art LLM-based agents.

Response: We thank the reviewer for the comment. We have included the entire performance evaluation test set for every agent in SI.zip as .csv files. Each line on the spreadsheet records a test case with its corresponding test input, ground truth labels, the testee agent’s output, the judge agent’s output, the final pass/failure. In addition, we also added analysis of failed test cases in the revised manuscript and SI.
For comparing to other state-of-the-art LLM-based agents, while our task-specific performance numbers are not directly comparable with other LLM benchmarks, the level of performance is considered to be higher than what is typical in LLM benchmarks for scientific tasks. For example, recent scientific-discovery benchmarks[2] typically report only 0.60–0.75 question-level accuracy across domains, while the best model in a question-clarification benchmark reaches a maximum success rate of 60.05%.[3] We have added discussions about the performance level in general LLM context in the revised manuscript.

Changes to text:
In Page 15, Section 2.2, we added

> “See SI.zip for spreadsheets that contain the test input, ground truth label, agent’s output, the judge agent’s output, and the pass/failure of every test case of each agent in MutexaGPT.”

In Page 13, Section 2.2, we added

> “Error analysis of the result of the *Full* QuestionAnalyzer shows that relevance failures are exclusively false negatives, primarily arising from overly vague inquiry (e.g., “What effect do ligands have on proteins?”), while effective-pass completeness failures are rare (3/100) and mostly associated with marginally ambiguous property definitions (e.g.: “flexibility of …”) rather than systematic inability to identify incomplete inquires.”

In Supporting Information, Page S20, we added Text S9 “Failure mode analysis in the agent performance evaluation”.
In Page 15, Section 2.2, we added

> “While not directly comparable, these performance levels are strong in the broader context of LLM benchmarks for scientific tasks (for reference, recent scientific-discovery benchmarks[61] typically report only 0.60–0.75 question-level accuracy across domains, while the best model in a question-clarification benchmark[62] reaches a maximum success rate of 60.05%).”

Minor:

6. The SI is disorganized with Text / Figures / Tables interspersed, and the large text boxes are particularly long and difficult to scroll through.

Response: We thank the reviewer for the comment. We will discuss with the production team about moving some of the SI content (especially the large text boxes) to files in SI.zip.

VU Station B#351822
2301 Vanderbilt Place
Nashville, Tennessee 37235-1822
Email zhongyue.yang@vanderbilt.edu
Phone 615.343.9849

7. Citations are missing on page 27 for the sentence “Existing LLM-agentic molecular simulation tools mostly operate at the assistant level, designed to streamline the molecular modeling process for domain experts.”

Response: We thank the reviewer for the careful check. We have fixed the citation.
Changes to text: In Page 31, Section 4, we fixed the citation.

8. Computational method page 30 mentions a “search through rotamer library to find the optimal side-chain position,” however methodological details necessary for reproducibility are not provided.

Response: We thank the reviewer for the comment. We have added the methodological details in the revised manuscript.
Changes to text:
In Page 34, Section 5a, we added

> “These structures are generated by replacing the mutating side chains in the wild-type structure and search through a rotamer library to find the optimal side-chain position. (using the *mutate_stru()* function in EnzyHTP)”

Reviewer #4 (Remarks to the Author):

Shao et al. present MutexaGPT, a web server that provides a natural language interface to make enzyme engineering informed by molecular dynamics (MD) simulations accessible to non-expert users. The multi-agent system, running on the now-discontinued GPT-4o large language model, processes a user's request by analyzing the user's intent, selecting appropriate metric(s) for the intended improvement, executing MD-based pipelines, and evaluating their results. The tool's capabilities are tested on two relevant enzyme engineering tasks: active-site enlargement to accommodate bulkier substrates and improving enzyme performance at lower temperatures. While the tool successfully identified improved enzyme variants in both cases, its success largely depends on the capability of MD-based methods to provide meaningful metrics that correspond to the user's intent. Additionally, the fast pace at which large language models are developed and commercial models are discontinued raises questions about the life-cycle of the presented tool. Nevertheless, MutexaGPT has the potential to become a meaningful addition to the computational enzyme engineering toolkit, by significantly lowering the barrier of entry to complex MD-based pipelines to a level that they become accessible to enzyme engineers with little to no previous experience in computational methods. Beyond the technical advancements, the ease of use benefits from an intuitive user interface and the provision of computational resources for computationally heavy simulations, both of which I strongly acknowledge. I would like the authors to address the following concerns:

Response: We thank the reviewer for the suggestions. The point-to-point response is provided below.

Major Concerns:

1. All experiments presented in the manuscript were obtained with the large language model (LLM) GPT-4o from OpenAI, which has been recently discontinued and is no longer publicly available, making the results irreproducible. Even though I don't think this drastically affects the usefulness of the presented tool, it raises a concern the authors mention themselves as the final statement of the manuscripts: AI models are developing at a fast pace and fully closed models cannot be seen as a long-lasting resource available to researchers. This necessitates benchmarking the system's performance across different LMMs in the backend. I recommend to analyse the performance with at least one comparable LLM with open-weigths. Specifically I would like to see the performance of the prompts with an open-weights model and how it would perform for the computational site in the two design campaigns because they will remain accessible in the future.

Response: We thank the reviewer for the suggestions. To clarify, although GPT-4o is no longer accessible in the ChatGPT web interface, it remains accessible through the OpenAI API for external applications like MutexaGPT. However, we fully agree that long-term reproducibility should not rely on a single closed commercial model. We have systematically refactored our entire website backend and added the support for multiple open-weights models. (e.g., Kimi K2.5, MiniMax-M2.5, and GLM-5, etc.) Users can now explicitly select which model they want to use in MutexaGPT (Figure R1), and for open-weights models no paywall/API key is required.

Following the reviewer's recommendation, we benchmarked all four core agents with an open-weight model (Kimi K2.5) and added full details (test input, ground truth labels, the testee agent's output, the judge agent's

VU Station B#351822
2301 Vanderbilt Place
Nashville, Tennessee 37235-1822
Email zhongyue.yang@vanderbilt.edu
Phone 615.343.9849

output, the final pass/failure) as spreadsheets in the SI package. The observed performance is similar to the original GPT-4o-based results: QuestionAnalyzer achieves 90% relevance pass and 96% effective completeness pass (they are 97% and 97% when previously GPT-4o is used), MetricsPlanner achieves 90% pass ratio (previously 91%), MutantPlanner achieves 98% pass ratio (previously 95%), and ResultExplainer achieves 96% overall pass ratio (previously 99%).

We also re-ran the two design campaigns with the open-weight LLM backend (Kimi K2.5) and obtained similarly effective conversation that set up the experiment and similarly informative text summary from about the computed results. In the cavity engineering case, the open model planned the same simulation and prioritized the same top-10 mutants as its suggestion. In the cold-adaptation case, the open model also planned the same simulation and prioritized the same two linker variants with superior domain separation as its suggestion for the following experimental test. We included the complete conversation logs and screenshots of final summary pages for these open-weight runs in the SI.

Changes to text:

In Page 34, Section 5a, Computational Method, *Configurations of the large language model (LLM)*, we added

> "Note that users can also choose the LLM model they want to use on the MutexaGPT website. (current options include: GPT-4o, GPT-5.4, Qwen3.6 Plus, MiniMax-M2.5, GLM-5, Kimi K2.5, GLM-4.7, Deepseek v3.2, Gemma 4 26B A4B IT, Gemma 4 31B IT)"

In Supporting Information, we added

> Text S4 "Example dialog between user and MutexaGPT for setting up the cavity engineering study using Kimi K2.5",
>
> Text S5 "Example dialog between user and MutexaGPT for setting up the cold-adaption study using Kim K2.5", and
>
> Text S11 "Example ResultExplainer output and Summary page screenshot for each use case. (model = kimi k2.5)"

In SI.zip, we added `agent_evaluation` which contains .csv spreadsheets of full performance evaluation details of every agent.

```
2. The authors claim multiple times that the presented tool helps to transform "physical-intuition" into "actionable insights" for enzyme engineering and claim to demonstrate this with the two enzyme engineering campaigns presented in the manuscripts. However, in both campaigns the tool is provided with a very specific set of mutations/linkers for which the tool should perform simulations. While the authors state that providing this information is optional or could be more coarse-grained, the performance of the tool in such a scenario remains unclear. To really see how the tool transforms the intuition of the user, the performance of the tool in the two engineering campaigns should be investigated with less information on the selected mutations upfront (e.g. define position only by distance from ligand). Alternatively, the manuscript should be heavily rephrased to avoid the impression that the tool is capable of running experiments without any prior knowledge of the activity at hand. Without the experiment of letting the tool identify potential mutations on its own, I don't see any notion of "intuition to actionable insights" as justified.
```

Response: We thank the reviewer for the suggestions. We agree that demonstrating performance under less specific prior input is necessary to support our claim. In the revised manuscript, we add a new use case in which mutation sites are defined only by the distance from ligand (i.e., all single-point mutations within 4 Å of the substrate). In order to be able to cross-reference existing experimental screening, we asked MutexaGPT to engineer towards the enzyme activity of a specific substrate. Under this setup, MutexaGPT completes the workflow by filtering candidates with folding free energy and ranking by electric field, yielding a top-10 set with a 30% hit rate after

cross referencing an experimental screen previously done by Tang *et al.*[4] Notably, this 4 Å mutation library contains 10 residue sites and 7 out of the 10 were screened for saturation mutagenesis by Tang *et al.* In our top-10 candidates, 3 mutants are experimental hits from Tang *et al.*'s experiment. Among the other 7 mutants, 3 are confirmed non-hits, and 4 mutants remain uncharacterized. This means that the actual hit rate could be higher than 30%. This performance is comparable to our original use case where more specific mutation information was provided upfront, supporting that the framework remains effective when users' prior knowledge is minimal. We include the full conversation log and result summary for this new use case in the SI.

Changes to text:

In Page 22, Section 3.1, we added

> "To further test whether MutexaGPT remains useful when prior information about mutational hotspot is minimal, we designed a test where the mutation library is selected solely based on a distance cutoff from the substrate. In this new case, the user starts by asking "Help me find mutations that make my enzyme faster for an non-native substrate ethyl iodide." and during the user's conversation with the QuestionAnalyzer, the mutation library was defined by user as "I want to explore all single point mutations with in 4A of ETI.", where ETI is the 3-letter name of the target substrate (i.e., ethyl iodide) in user's PDB files. After the conversation, MutexaGPT plans a workflow that calculates the folding free energy and the electric field strength projected to the breaking C–I bond for each enzyme mutant. With the simulation data, MutexaGPT summarizes the results by filtering candidates with folding free energy and ranking by electric field, yielding a top-10 mutant list, of which 3 mutants are experimentally active in a previous screening reported by Tang *et al.*[4], corresponding to a hit rate of 30%. Notably, this 30% value serves a lower bound because 4 mutants on this list were not experimentally characterized and are therefore not labeled in our test. Overall, this low-prior-knowledge setup results in a similar prediction performance to the cavity engineering example, supporting MutexaGPT's ability to convert broadly-defined, less-informed design intent into actionable screening priorities."

In Supporting Information, Page S7, we added Text S3 "Example dialog between user and MutexaGPT for setting up the non-native activity engineering study."

In Supporting Information, Page S12, we added Text S6 "Example dialog between user and MutexaGPT for setting up the non-native activity engineering study using Kimi K2.5."

In Supporting Information, Page S22, Text S10 "Example ResultExplainer output and Summary page screenshot for each use case.", we added subsection: *Use case 3: Non-native Enzyme Activity Engineeering*

In Supporting Information, Page S38, Text S11 "Example ResultExplainer output and Summary page screenshot for each use case. (model = kimi k2,5)", we added subsection: *Use case 3: Non-native Enzyme Activity Engineeering*

3. Even though I generally think the manuscript is well written, it contains multiple lurid claims that are not supported by the data. It's important to point out that the tool can help in identifying mutants with improved properties only if MD-based metrics can provide meaningful insight into the property of interest. For example, the cavity engineering campaign recovered only 4 of the 10 best-performing mutants, and the data suggest that this is due to fundamental limitations of the MD-derived cavity volume metric in fully capturing differences in activity across substrates. Unfortunately, this limitation is not discussed in the manuscript. Rather, the tool's capabilities are displayed to "enable a new paradigm for enzyme engineering where human creativity is seamlessly integrated with computational simulation to make new discoveries and designs" (L503f), which is simply overselling the tool's capability given the presented data. I would like the authors to reflect more carefully on the limitations of the tools and recommend revising the following (but not limited) sections of the manuscript: L122-125, L279f., L351-L353,

VU Station B#351822
2301 Vanderbilt Place
Nashville, Tennessee 37235-1822
Email zhongyue.yang@vanderbilt.edu
Phone 615.343.9849

L502-L505.

Response: We thank the reviewer for the suggestions. We agree that our previous wording could overstate the current utility of MutexaGPT. In the revised manuscript, we have tempered these claims as well as emphasized the the limitation of MutexaGPT in its metrics & MD dependence. We have also improved the ResultExplainer agent in MutexaGPT to make it capable of reporting the limitation/caveat of the applied metrics (e.g., the limitation of just using cavity volume). ResultExplainer now contains two complementary paragraphs: "What it may indicate," which explains the physical model underlying the metric and its relevance to the user's original scientific question, and "What it does NOT directly prove," which alerts users to potential overinterpretation or misinterpretation of the results. The user will also receive suggestions about methodological improvements based on the results (e.g., computing more metrics for the top-ranking variants). We have added the raw output of ResultExplainer and screenshots of the Summary page to the Supporting Information (Text S10). In the main text, we updated the discussion about ResultExplainer at the section where it is first introduced.

Changes to text:

In Page 1, Abstract, we revised

> "Here we introduce MutexaGPT, an open-access, multi-agent large language model (LLM) platform that translates enzyme engineering intuition to physics-based simulations and thus variant designs."

In Page 5, Section 1, Introduction, we added

> "Note that although MutexaGPT ranks and filters enzyme variants based on computed metrics, it is not a predictive AI tool, but instead an integrated system for automation and intuition-translation."

In Page 6, Section 1, Introduction, we revised

> "Complementary to screening-dominated approaches, MutexaGPT provides a scalable route to operationalize intuition-driven, physics-guided enzyme engineering within the scope of currently implemented metrics and simulations."

In Page 5, Section 1, Introduction, we added

> "Note that although MutexaGPT ranks and filters enzyme variants based on computed metrics, it is not a predictive AI tool, but instead an integrated system for automation and intuition-translation."

In Page 28, Section 4, Discussion, we added

> "Notably, while MutexaGPT ranks and filters enzyme variants based on computed metrics, it is not a predictive AI tool, but instead an integrated system for automation and intuition-translation."

In Page 29, Section 4, we added

> "How does MutexaGPT work beyond the two case studies? Currently, MutexaGPT supports high-throughput calculations of a list of function-related metrics: … Note that a practical limitation of MutexaGPT also comes from this list of computable metrics. If a property of interest from the user cannot be mapped to a currently supported EnzyHTP-computable metric, MutexaGPT cannot produce a physically grounded simulation answer and should instead return an explicit unsupported-property notice. Nevertheless, MutexaGPT is designed to be extensible, where new metrics can be easily (by changing just two files if they are supported by EnzyHTP) added in the future to support more enzyme engineering tasks (One can refer to Table S1 for currently supported metrics; We should note that the underlying EnzyHTP engine is developed with interface modules to common software such as Amber for trajectory analysis, Gaussian/xTB for quantum mechanics, Multiwfn for wavefunction analysis, Rosetta for biophysics, etc. and thus inherits the metrics-support-boundary of these software)."

In Page 10, Section 2.1, we updated

> "Acting under the prompt shown in Supplementary Table S2, Result Explainer handles five top-level goals: (1) translate raw modeling results into concise, domain-relevant takeaways; (2) anchor every explanation to the user's scientific question rather than to the data in isolation; (3) explain, for each reported metric, what it may indicate biologically or structurally and, equally importantly, what it does not directly measure or prove, thereby reducing the risk of over-interpretation by non-computational users; (4) communicate the confidence and limitations of the analysis in light of the available simulation context, such as simulation length and equilibration information; and (5) prioritize the most relevant variants and recommend follow-

up experiments, additional simulations, or data checks. (e.g., ResultExplainer can rank/filter enzyme variants based on computed metrics through functional tools; see Text S10 for examples of ResultExplainer output)."

In Supporting Information, Page 22, "Text S10. Example ResultExplainer output and Summary page screenshot for each use case", we added subsections: "*Use case 1: Cavity Engineering*" and "*Use case 2: Cold Adaptation Engineering*".

4. I recommend including an overview of all metrics supported in the current version of the tool. Further, the tool's usefulness depends heavily on the metrics' usefulness for engineering campaigns. I recommend discussing this relation in more depth based on the existing literature and focusing less on metrics that could be added in the future, which, at this moment, can only be treated as speculative.

Response: We thank the reviewer for the suggestions. We have added a new SI table that lists metrics MutexaGPT currently supported and the scientific questions they are related to. We have also added discussion of the metrics-related limitations in the Discussion section.

Changes to text:

In Page 29, Section 4, we revised

"How does MutexaGPT work beyond the two case studies? Currently, MutexaGPT supports high-throughput calculations of a list of enzyme function-related metrics: electric field strength, substrate positioning index (SPI), active site RMSD, MM-PB/GBSA ligand binding energy, relative folding free energy, cavity volume, and domain separation index (DSI). Beyond the non-native substrate accommodation (metric: cavity volume) and cold adaptation (metric: DSI) as demonstrated in the current study, this full metrics list can be applied to a large variety of enzyme engineering tasks. For example, the cavity volume metric guides the engineering of enantioselectivity.[33, 44, 45] Electric field strength can guide the engineering of rate enhancement for enzymes that catalyze reactions involving a substantial change of charge and polarity state from the reactant state to the transition state.[20-25] MMPB/GBSA ligand binding energy can guide the engineering of enzyme with enhanced binding affinity to the substrate or reduced product inhibition.[81] SPI can guide the engineering of catalytic efficiency for enzymes where precise substrate positioning is critical for catalysis.[82] Relative folding free energy can be used to guide the engineering of enzyme stability. Combining these metrics is expected to address more complex challenges, such as studying drug resistance mutations. Note that a practical limitation of MutexaGPT also comes from this list of computable metrics. If a property of interest from the user cannot be mapped to a currently supported EnzyHTP-computable metric, MutexaGPT cannot produce a physically grounded simulation answer and should instead return an explicit unsupported-property notice. Nevertheless, MutexaGPT is designed to be extensible, where new metrics can be easily (by changing just two files if they are supported by EnzyHTP) added in the future to support more enzyme engineering tasks. (One can refer to Table S1 for currently supported metrics; We should note that the underlying EnzyHTP engine is developed with interface modules to common software such as Amber[83] for trajectory analysis, Gaussian[84]/xTB[85] for quantum mechanics, Multiwfn[86] for wavefunction analysis, Rosetta[87] for biophysics, etc. and thus inherits the metrics-support-boundary of these software) Overall, we envision MutexaGPT can be applied to a wide range of enzyme engineering tasks by leveraging the existing metrics and adding new metrics in the future."

In Supporting Information, we added Table S1 "Metrics MutexaGPT currently supports".

5. The analysis of the tools' performance with different prompt designs needs some clarification on the how it was evaluated. L216-218 states that the performance was tested across three runs. To what does "run" refer here? Was the agent prompted three times with the same question and evaluated three

VU Station B#351822
2301 Vanderbilt Place
Nashville, Tennessee 37235-1822
Email zhongyue.yang@vanderbilt.edu
Phone 615.343.9849

times by the evaluator LLM? Or do runs refer to successfully executed MD tool calls? This section and the corresponding section in the supplementary material would benefit from a more detailed explanation.

Response: We thank the reviewer for the comment. In the agent performance evaluation, three "runs" refers to three independent executions of the agent with the exact same input and then evaluated by the same agentic evaluator. We have added more details explaining the agent performance evaluation in the revised manuscript and SI. We have included the entire test set for every agent in SI.zip as .csv files. Each line in these files records a test case with its corresponding test input, ground truth labels, the testee agent's output, the judge agent's output, the final pass/failure.

Changes to text:

In Page 15, Section 2.2, we added

> "See SI.zip for spreadsheets that contain the test input, ground truth label, agent's output, the judge agent's output, and the pass/failure of every test case of each agent in MutexaGPT."

In SI.zip, we added `agent_evaluation` which contains full performance evaluation details of every agent.

In Page 14, Section 2.2, we added

> "For MetricsPlanner, the test-set curator generates inputs (that matches the format of the QuestionAnalyzer's final output; 100 cases asking about properties that can map to one of our supported metrics, and 100 cases asking about properties that are not) from ground-truth metric/MD-model labels, and a case passes only when the agent's recommended candidates contain the labeled metric-model pair or inform user when none of the supported metrics can correspond to the inquiry. For MutantPlanner, we used a set of manually curated inputs. It contains a balanced set of unrestricted, pocket, protein-protein interface, distal (relative to the pocket) mutational libraries, and designated site mutations (10 for each, 50 in total). The correctness is judged by whether the generated EnzyHTP mutation pattern is semantically equivalent to the labeled library (i.e., they are parsed by EnzyHTP and directly compared for equivalence). For ResultExplainer, a set of JSON payloads are curated by a curator agent (containing "scientific question", "metrics", "results", "downloadables", and "metadata"; 100 in total). The outputs of ResultExplainer are evaluated by two judge agents, one assessing how well the output aligns to the scientific question from the input and the other assessing interpretation-boundary discipline (i.e., avoidance of overclaiming beyond the provided data), and overall pass requires satisfying both criteria. Full benchmark inputs, pass definitions, and per-run outputs are provided in Text S8 and SI.zip/agent_evaluation.
>
> The results show that the prompt design for each agent is highly effective in their respective roles: for WorkPlanningBoard, MetricsPlanner on average passes 91% of its test cases, MutantPlanner achieves a 95% success rate; ResultExplainer met all evaluation criteria in 99% of the test cases. While not directly comparable, these performance levels are strong in the broader context of LLM benchmarks for scientific tasks (for reference, recent scientific-discovery benchmarks[65] typically report only 0.60–0.75 question-level accuracy across domains, while the best model in a question-clarification benchmark[66] reaches a maximum success rate of 60.1%). See Text S9 for error analysis of each agent. See SI.zip for spreadsheets that contain the test input, ground truth label, agent's output, the judge agent's output, and the pass/failure of every test case of each agent in MutexaGPT."

In Supporting Information, Page S20, Text S8 "The evaluation system of LLM-agents.", we added three sections under the existing example of QuestionAnalyzer, for each other agents:

> "The detailed evaluation design of all other agents besides QuestionAnalyzer mentioned above are summarized below:
>
> *MetricsPlanner.* To evaluate MetricsPlanner, we use a …
>
> *MutantPlanner.* MutantPlanner is evaluated with ...
>
> *ResultExplainer.* For ResultExplainer, test inputs are curated … "

6. It should be pointed out clearly that using the tool requires a paid OpenAI API key

Response: We thank the reviewer for the suggestions. We have revised the manuscript accordingly to point this out.
Changes to text: In Page 34, Section 5a, we added "When using the OpenAI models, users need to provide their own OpenAI API keys."

Minor Concerns:
7. The survey results (Figure 1A, Figure S3) are likely not representative and should be removed. I think readers of this paper will be aware of the steep learning curve MD simulations come along with, even without the results of the survey. I trust the main conclusions of the survey, but I fear the survey results could be cited in the future, even though the survey design might not reflect the state of the art and might not be representative.

Response: We thank the reviewer for the suggestions. We agree that the survey is likely not representative. We have removed all survey-related content as suggested by other reviewers.
Changes to text:
In Section 1, we removed the sentences about the survey.
In Section 1, we removed Figure 1A.
In Supporting Information, we removed Figure S3.

8. I recommend to cite Ai.zymes (https://doi.org/10.1002/anie.202507031) as another example of an automated pipeline that relies on MD-based metrics for user-friendly enzyme engineering, and DynaMate as an AI agent for preparing and running MD simulations for protein-ligand systems (https://doi.org/10.48550/arXiv.2512.10034)

Response: We thank the reviewer for the suggestions. We have added the citations in the revised manuscript.
Changes to text:
In Page 4, Section 1, Introduction, we added

> "and AI.zymes[51] integrated MD screening into a multi-objective design workflow, using metrics such as electric field strength, ligand flexibility, etc. to prioritize variants for improving the promiscuous Kemp eliminase activity in a ketosteroid isomerase."
> And
> "… LLM-agentic molecular simulation tools (e.g., MDCrow[52], NAMD-agents[53], DynaMate[54], and ProtAgents[55])"

9. For the cavity engineering campaign, I believe another important comparison to understand the necessity of MD simulations would be co-folding of the mutated sequences and ligands with AlphaFold3 or Boltz2 and measuring the change in cavity volume in the predicted structures.

Response: Thank you for this thoughtful suggestion. As shown in Figure R2, we performed Boltz-2 prediction on *acl*-MT variants (the AlphaFold server does not support our the ligand SAH in our enzyme), we found that its predicted structure is not sensitive to the point mutations (e.g., 2-5 mutations). In our top-candidate with the largest predicted cavity, L39H V11F, Boltz-2 give a very similar structure to its prediction on the wild-type (RMSD = 0.16). The shape of the cavity is almost identical in the predicted mutant and wild-type (Figure R2, blue). This observation is consistent with recent reports that current co-folding foundation models are relatively insensitive to subtle mutation-induced active-site changes. Specifically, the benchmarks indicate that AlphaFold3, RosettaFold, Boltz-1, and Chai-1 can retain bias toward the original pocket geometry even after binding-site mutations[5], and

VU Station B#351822
2301 Vanderbilt Place
Nashville, Tennessee 37235-1822
Email zhongyue.yang@vanderbilt.edu
Phone 615.343.9849

Boltz2-based predictions are insufficiently responsive to key binding-site substitutions in a recent benchmark[6]. In contrast, the shape of the cavity from a representative MD substantially deviates from the static structure due to its equilibration in water at ambient conditions. Although a larger scale investigation on the cavity prediction capability by AF3/Bolt-2 could be an interesting direction, we consider the analysis goes beyond the scope of the present study that focuses on demonstrating the practical utility of MutexaGPT for guiding enzyme engineering campaigns. A rigorous AF3/Boltz2 comparison would require a dedicated workflow, additional controls, and separate benchmarking criteria, and is therefore an interesting topic for a follow-up investigation.

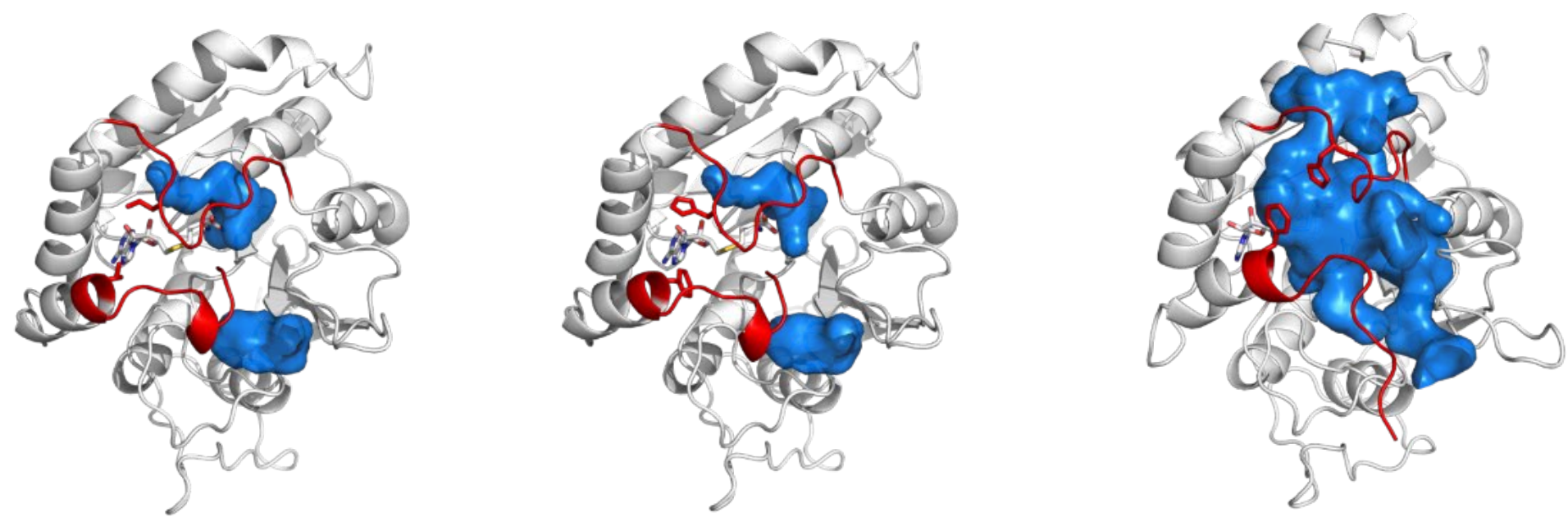

Figure R2. Boltz-2 predicted structures and a representative structure from the MD simulation of *acl*-MT variants. The mutated residues are shown as sticks, red. The key active site loop is shown as cartoon, red. The active site cavity is shown as surface, blue. The representative MD structure is collected from the distribution center of the cavity volume distribution in the conformational ensemble sampled from the MD.

10. For the experimental data of the cold adaptation campaign, I recommend including a plot that shows the unnormalised activity of the wildtype and tested variants in the supplementary information

Response: We thank the reviewer for the suggestions. We have a table (Table S4) in the Supporting Information reporting the un-normalized activity of the wildtype psA and tested variants (psAt5, psAt6).

11. I appreciate the option to run the computationally heavy simulations through the webapp, while also providing the option to download scripts for running simulations locally. I was wondering if there are any limitations on the number of simulations or the simulation length. If so, it would be useful to state them in the manuscript and the webapp.

Response: We thank the reviewer for the comment. Currently, there is no limitation on the number of simulations nor simulation length. (There was a limitation of 6 mutants in the development test stage. Now this limitation has been lifted.)

12. I could not find any information on how the data the user uploads to the webapp is processed with regard to privacy. I would recommend to include information on this in the webapp

Response: We thank the reviewer for the suggestion. Our privacy policy is listed at https://enzyhtp.app.vanderbilt.edu/docs.html?doc=privacy.

13. The code repository is missing a licence that defines if and under which

```
conditions someone is allowed to use the software. Any type of licence should
be added to avoid confusion.
```

Response: We thank the reviewer for the suggestion. We have added a license file to the repository.

```
14. The experimental results mentioned in L346-L350 miss a citation from which
they are derived.
```

Response: We thank the reviewer for the comment. We have fixed the citation.

```
15. I recommend making the dataset from the prompt evaluation experiment
publicly available to allow for custom prompt engineering of users with
automated methods (e.g. GEPA)
```

Response: We thank the reviewer for the suggestions. We have added the full dataset in the revised SI.zip.
Changes to text: In SI.zip, we added `agent_evaluation` which contains full performance evaluation dataset.

```
16. The webapp has many Icons corresponding to features under construction.
Will these actually be implemented in the future?
```

Response: We thank the reviewer for the comment. Yes, we have a team working on them.

References

1 Chodera, J. D. A Simple Method for Automated Equilibration Detection in Molecular Simulations. *J. Chem. Theory Comput.* **12**, 1799-1805 (2016). https://doi.org:10.1021/acs.jctc.5b00784
2 Song, Z. *et al.* Evaluating Large Language Models in Scientific Discovery. arXiv:2512.15567 (2025). <https://ui.adsabs.harvard.edu/abs/2025arXiv251215567S>.
3 Gan, Y. *et al.* ClarQ4LLM: A Benchmark for Models Clarifying and Requesting Information in Task-Oriented Dialog. *IEEE Transactions on Audio, Speech and Language Processing* **34**, 1441-1456 (2026). https://doi.org:10.1109/taslpro.2026.3661250
4 Tang, Q. *et al.* Directed Evolution of a Halide Methyltransferase Enables Biocatalytic Synthesis of Diverse SAM Analogs. *Angew. Chem. Int. Ed.* **60**, 1524-1527 (2021). https://doi.org:10.1002/anie.202013871
5 Masters, M. R., Mahmoud, A. H. & Lill, M. A. Investigating whether deep learning models for co-folding learn the physics of protein-ligand interactions. *Nat. Commun.* **16** (2025). https://doi.org:10.1038/s41467-025-63947-5
6 Bret, G., Sindt, F. & Rognan, D. Assessing Boltz-2 Performance for the Binding Classification of Docking Hits. *J. Chem. Inf. Model.* **66**, 1511-1521 (2026). https://doi.org:10.1021/acs.jcim.5c02630

VU Station B#351822
2301 Vanderbilt Place
Nashville, Tennessee 37235-1822
Email zhongyue.yang@vanderbilt.edu
Phone 615.343.9849

VANDERBILT UNIVERSITY
COLLEGE OF ARTS AND SCIENCE

Department of Chemistry

July 7th, 2026

Kaitlin McCardle, PhD
Senior Editor
Nature Computational Science
Nature portfolio

Dear Dr. McCardle:

Thank you and the reviewers for the constructive comments and evaluation of our manuscript entitled "MutexaGPT: An Intuition-to-Design Translator for Physics-based Enzyme Engineering" (manuscript ID **NATCOMPUTSCI-25-4365A**). We have now addressed the comments and requests from the reviewers and submitted our revised version. The original reviewers' comments are provided in full on this letter with a response to each comment labeled "Response" alongside a reproduction of corresponding changes to the text with associated page numbers. The revised manuscript with marked edits is labeled as *mss_marked.pdf*.

Here is a brief summary of the main findings of the paper (within 250 characters):

MutexaGPT turns plain-language enzyme-engineering intuitions into lead mutation designs through AI agents-orchestrated high-throughput molecular modeling, yielding experimentally validated improvements in enzyme specificity and cold activity.

I am the submitting author and my contact information is given below. All authors have seen and approved the submission of this manuscript, and it is being submitted exclusively to *Nature Computational Science.* If there are any questions, please do not hesitate to contact me at the e-mail address or phone number listed below.

Sincerely,

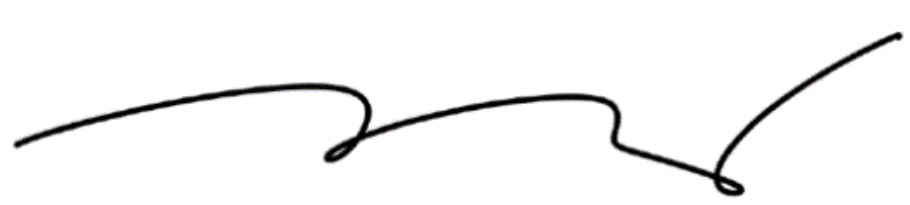

Prof. Zhongyue (John) Yang
Department of Chemistry
Data Science Institute
Email: zhongyue.yang@vanderbilt.edu
Phone: (615) 343-9849

VU Station B#351822
2301 Vanderbilt Place
Nashville, Tennessee 37235-1822
Email zhongyue.yang@vanderbilt.edu
Phone 615.343.9849

Reviewer #1:
Remarks to the Author:
All raised points have been satisfactorily addressed. No further comments.

Reviewer #2:
Remarks to the Author:
All raised comments were satisfactorily addressed. No further comments.

Reviewer #3:
Remarks to the Author:
The authors have thoroughly addressed the majority of my previous concerns. A few minor comments remain:

Response: We thank the reviewer for the comments. The point-to-point response is provided below.

1) MutexaGPT runs several software
"interface modules to common software such as Amber83 for trajectory analysis, Gaussian84/xTB85 for quantum mechanics, Multiwfn86 for wavefunction analysis, Rosetta87 for biophysics, etc." some of which require a license to run. Are the authors certain they are not violating terms of any of these by freely enabling others to run their programs through MutexaGPT? Do individuals need to confirm they have their own license, or will the program simply not run and output scripts in certain cases?

Response: We thank the reviewer for raising this important point.
MutexaGPT supports two distinct modes of use: 1) executing the simulation in our backend or 2) exporting scripts for execution in user's own environment.
In the first case, backend execution is available only to users who provide evidence of their own license. They will be asked to upload a proof before the job can run in the backend.
In the latter case, MutexaGPT does not run or redistribute licensed third-party software; users execute the prepared files in their own licensed computing environments.
We have revised the manuscript to make this distinction explicit.

Changes to text: On Page 18, under the Method section, we added
"MutexaGPT does not redistribute proprietary third-party software; backend execution of license-restricted software is available only to users who provide evidence of appropriate authorization, whereas otherwise MutexaGPT outputs scripts and input files for execution in the user's own licensed environment."

2) If input scripts are output rather than using the server, this could be a challenge for non-computationalists. Different servers have different queue's / syntax / installed software. It is not clear how this function will actually be utilized.

Response: We thank the reviewer for the comment. When users choose to export input scripts rather than run jobs through the MutexaGPT server, they can adapt the submission syntax of their local HPC environment by modifying a simple configuration file, md_cli_config.json, included in the script package exported by MutexaGPT. This file allows users to specify local HPC syntax such as the submit, status, and cancel commands, as well as the job script_template. MutexaGPT then uses this configuration file to generate the submission scripts for each job. The generated scripts are also plain-text files, so users can examine and modify them before submission if their local queue syntax or software environment requires additional changes. We agree that this mode may be challenging for non-computationalists without HPC experience. Therefore, the exported-script workflow is intended primarily for

users who have some familiarity with using local HPC systems and access to such computing resources.

Changes to text: On Page 23, Section 4c, we added:

"The platform also provides users with the option to download a complete folder of prepared input files for all required simulations, together with the exact EnzyHTP Python script and a lightweight configuration file, md_cli_config.json, for adapting the workflow to the syntax of their local HPC environment. Through this configuration file, users can specify site-specific job-management commands, including job submission, status checking, cancellation, and the job-script template required by their local queueing system. MutexaGPT then uses this information to generate plain-text submission scripts for each simulation job, which users can inspect and modify before submission if their local queue syntax, module system, or software environment requires additional customization. The resulting simulations can be executed using the users' local computing resources, and the outputs can be uploaded back to MutexaGPT for downstream analysis if needed. To further support reproducibility, particularly when users wish to publish data generated through MutexaGPT, the workflow review page allows users to export the exact EnzyHTP Python script executed by the platform. This script records the full simulation setup, parameters, and workflow logic, and can therefore be directly rerun, archived, or cited in the Methods or Supplementary Information sections."

3) The authors should state more clearly that the first test case is a retrospective validation while only the second is prospectively experimentally validated.

Response: We thank the reviewer for the suggestion. We have revised the manuscript accordingly.

Changes to text: In Page 6, Section 2, Results, we added:

"First, a cavity-engineering task … the resulting mutants were then test retrospectively against previously reported experimental screening results. Second, a cold-adaptation engineering task … the resulting design were subsequently evaluated by our experimental activity assays."

4) If significant portions of the code were written by AI coding assistants, the authors should disclose this.

Response: We thank the reviewer for the suggestions. We have revised the manuscript accordingly.

Changes to text: On Page 30, Data and code availability, we added:

"(OpenAI Codex was used as an AI coding assistant during the development of MutexaGPT, and all final code was reviewed and curated by the authors.)"

Reviewer #4:

Remarks to the Author:

I highly appreciate the extensive additions and evaluations in the revised manuscript. The authors have fully addressed my previous concerns regarding long-term reproducibility and framing the tool's capabilities. I believe MutexaGPT will be a valuable tool for non-computational researchers, making MD-guided protein engineering more accessible. I have no further concerns and recommend accepting the manuscript for publication.

VU Station B#351822
2301 Vanderbilt Place
Nashville, Tennessee 37235-1822
Email zhongyue.yang@vanderbilt.edu
Phone 615.343.9849
